# Molecular organization and phylogenetic analysis of 5S rDNA in crustaceans of the genus *Pollicipes *reveal birth-and-death evolution and strong purifying selection

**DOI:** 10.1186/1471-2148-11-304

**Published:** 2011-10-17

**Authors:** Alejandra Perina, David Seoane, Ana M González-Tizón, Fernanda Rodríguez-Fariña, Andrés Martínez-Lage

**Affiliations:** 1 Department of Cell and Molecular Biology. Evolutionary Biology Group (GIBE), Universidade da Coruña, A Fraga 10, E-15008 A Coruña, Spain

## Abstract

**Background:**

The 5S ribosomal DNA (5S rDNA) is organized in tandem arrays with repeat units that consist of a transcribing region (5S) and a variable nontranscribed spacer (NTS), in higher eukaryotes. Until recently the 5S rDNA was thought to be subject to concerted evolution, however, in several taxa, sequence divergence levels between the 5S and the NTS were found higher than expected under this model. So, many studies have shown that birth-and-death processes and selection can drive the evolution of 5S rDNA. In analyses of 5S rDNA evolution is found several 5S rDNA types in the genome, with low levels of nucleotide variation in the 5S and a spacer region highly divergent. Molecular organization and nucleotide sequence of the 5S ribosomal DNA multigene family (5S rDNA) were investigated in three *Pollicipes *species in an evolutionary context.

**Results:**

The nucleotide sequence variation revealed that several 5S rDNA variants occur in *Pollicipes *genomes. They are clustered in up to seven different types based on differences in their nontranscribed spacers (NTS). Five different units of 5S rDNA were characterized in *P. pollicipes *and two different units in *P. elegans *and *P. polymerus*. Analysis of these sequences showed that identical types were shared among species and that two pseudogenes were present. We predicted the secondary structure and characterized the upstream and downstream conserved elements. Phylogenetic analysis showed an among-species clustering pattern of 5S rDNA types.

**Conclusions:**

These results suggest that the evolution of *Pollicipes *5S rDNA is driven by birth-and-death processes with strong purifying selection.

## Background

In higher eukaryotes, nuclear ribosomal DNA (rDNA) genes are usually organized in two multigene families, each composed of hundreds to thousands of copies. A major family encodes for 28S, 5.8S, and 18S rRNA, and a minor family contains only 5S rRNA genes. The 5S rDNA consists of a conserved transcribing region of 120 bp (hereafter 5S) with a variable intergenic spacer usually referred to as the nontranscribed spacer (NTS). The 5S region is highly conserved in length and sequence even among unrelated species, although there is a high rate of heterogeneity within the NTS region among closely related species. This variation of NTS is due to insertions, deletions, mini-repeats, base-substitutions and pseudogenes and has been used for evolutionary studies and as a source of species-specific or population specific markers [1-3].

The evolution of ribosomal gene families recently became controversial after it was analyzed in several taxa. The evolution of 5S rDNA units has classically been explained by the concerted evolution model, which suggests that molecular mechanisms such as gene conversion and unequal crossing-over play an important role in the homogenization of repeated units. These mechanisms maintain a high sequence similarity between copies and prevent the independent evolution of each member of a multigenic family [4]. However, several cases have been reported in which sequence divergence levels between ribosomal genes or spacers seem to be much higher than would be expected under a strict concerted evolution scenario [5]. So, many studies have shown that birth-and-death processes and selection can drive the evolution of 5S rDNA in distantly related taxa [3,5-9]. Under the birth-and-death evolution model, new variants are created by gene duplication and can remain as functional genes in the genome or become pseudogenes. In this way, transcribing region conservation could be explained by purifying selection, as suggested by Nei and Rooney [10].

The genus *Pollicipes *consists of four species: *P. pollicipes*, *P. elegans*, *P. polymerus *and *P. caboverdensis*. These stalked barnacles are sessile pedunculate cirripede occurring in dense aggregations exposed to heavy swell on rocky intertidal sites. Distribution of *P. pollicipes *(Gmelin 1789) is on the northeastern Atlantic coast, from Dakar in Senegal to the north coast of Brittany in France [11,12]. *P. elegans (*Lesson 1831) is found on the west coast of South America from Mexico to Peru, and *P. polymerus *(Sowerby 1833) is common in the intertidal region of more exposed parts of the west coast of North America [11]. *P. polymerus *overlaps *P. elegans *at its southern limit, and *P. caboverdensis *[13] occurs off the Cape Verde Islands.

Studies focused on 5S rDNA have been performed on a small number of crustacean species and, different genomic organization types found. So, in some crustaceans 5S rDNA genes are linked to the major ribosomal genes [14,15] whereas in *Artemia salina *and *Asellus aquaticus *they are linked to the tandem repeats of the histone genes [16,17]. 5S rDNA genes are also linked to U1 small nuclear DNA (snDNA) in *A. aquaticus *[18], whereas they are unlinked to other multigene families in *Proasellus coxalis *[19].

In the present study, the nucleotide sequences, molecular organization and secondary structure of the 5S rDNA were investigated in three species of the genus *Pollicipes *(*P. pollicipes, P. elegans*, and *P. polymerus*) to know the evolution of these genes in this group of crustaceans.

## Results

### Nucleotide sequence analysis of 5S rDNA

A total of 116 5S rDNA sequences from the genomes of *P. pollicipes*, *P. elegans *and *P. polymerus *was obtained experimentally to study the molecular evolution of 5S rDNA. PCR amplification generated different fragments with different size of 5S rDNA units, as among the different species as within the same species.

A different electrophoretic pattern was obtained in each of the three species. Six bands of approximately 200 bp, 280 bp, 350 bp, 400 bp, 440 bp, and 600 bp were observed in *P. pollicipes*. Four bands were found in two other species: 350 bp, 550 bp, 700 bp and 900 bp in *P. elegans*, and 440 bp, 600 bp 870 bp and 1040 bp in *P. polymerus*. Sequence-similarity searches showed that all sequences matched other 5S rDNA, and BLASTN analysis of the NTS region did not detect any significant similarity with sequences from any other organisms.

Primers were designed in such a way that only tandemly arranged 5S rDNA units could yield amplification products. Most of them corresponded to monomers formed by the last portion of the 5S (88 bp), the NTS, and the first portion of the contiguous 5S (32 pb). To maintain the similarity with other 5S rDNA sequences from the international nucleotide sequence databases, the 3' end of the 5S was transferred to the 5' end. In all species analyzed here, we obtained several dimer sequences and a trimer in *P. elegans *(906 bp), formed by two and three contiguous monomers respectively (see Additional File 1, Figure S1). Sequences were named with the letters a, b, and c when they were the first, second, or third unit of the array. The b and c sequences had a complete 5S + NTS units, respectively. From these sequences we designed a more specific primer (5S-Poll-R2) which differs by only 2 nt from 5S-Poll-R. By using the 5S-Poll-R2 primer, we amplified 82 out of 116 sequences obtained in this work. Almost all dimers were homogeneous (both monomers were identical or almost identical), but in one clone of *P. polymerus *we detected a 5S rDNA unit of 1041 bp which consisted of two monomers of 605 and 436 bp. Therefore, we have discovered the linkage of two different units. The trimer was also composed of different monomers. Two of them consisted of the same repeat, but the other had a completely different nucleotide sequence in its NTS. In *P. pollicipes *only two dimer sequences (407 bp) were formed by divergent monomers, the shortest of which consisted of 123 bp (see Additional File 2, Table S1). These sequences were considered truncated pseudogenes because they lacked seven nucleotides in the 5S region and the spacer. These sequences were not included in the subsequent analyses.

The 5S region showed a high GC content (59.2%) and was 120 bp long in all sequences except one, Py02Oly03 (119 bp). It displayed 27 polymorphic sites which were analysed excluding the primer-annealing regions in the a sequences whereas these regions were studied in the b and c sequences because they present a complete 5S + NTS units, respectively.

Regarding NTS analyses, the initial alignment showed that the NTSs of genus *Pollicipes *were highly divergent, revealing the existence of different types (see Additional File 3, Figure S2). The TGI Clustering Tools showed seven types of NTS that we named using letters from A to G. The putative pseudogenes were not classified. A local BLAST allowed us to confirm the types and to classify two doubtful sequences into their corresponding types through the E-value. We also carried out a BLAST among sequences that belonged to different types. In most cases there was no similarity among them when we used megablast except in sequences of F and G types, in which case the E value was 10 ^-100 ^or less. F and G types have the same nucleotide sequence, with some fixed nucleotide substitutions and large insertions that increase the length of the sequence from 436-448 (F type) to 604-605 bp (G type).

The NTS showed a high degree of variation produced by several insertion-deletion polymorphisms (indels) and nucleotide substitutions. The size of the NTS region was highly variable, ranging from 78 to 489 bp. In *P. pollicipes*, the lengths of the 5S rDNA units ranged between 605-609 (A type), 203-207 (B type), 284 (C type), 353 (E type) and 436 bp (F type); in *P. elegans *they were 198 to 351-357 bp (D and E types respectively); and in *P. polymerus*, they were 436-448 bp for the F type sequences and 604-605 bp for the G type ones.

This length disparity in the F type of *P. polymerus *(436-448 bp) is due to an insertion of 12 bp in the 324 position. The NTS minimum average size was 83 bp (78-87bp). The number of polymorphic sites is given in Table 1.

**Table 1:** 5S rDNA polymorphism by species within types

	region	n	s	h	π
**A type**	5S rDNA		69	34	0.018
*P. pollicipes*	120 pb	35	5	5	0.004
	nts		64	31	0.021

**B type**	5S rDNA		11	2	0.036
*P. pollicipes*	120 pb	3	1	2	0.006
	nts		10	2	0.080

**C type**	5S rDNA		23	2	0.054
*P. pollicipes*	120 pb	3	6	2	0.033
	nts		17	2	0.069

**D type**	5S rDNA		4	2	0.020
*P. elegans*	120 pb	2	4	2	0.033
	nts		0	1	0.000

**E type**	5S rDNA		16	3	0.025
*P. pollicipes*	120 pb	4	2	2	0.008
	nts		14	3	0.034
	
**E type**	5S rDNA		66	37	0.037
*P. elegans*	120 pb	43	9	9	0.015
	nts		57	29	0.048

**F type**	5S rDNA		7	4	0.009
*P. pollicipes*	120 pb	4	2	2	0.011
	nts		5	3	0.008
	
**F type**	5S rDNA		35	1	0.017
*P. polymerus*	120 pb	17	5	5	0.009
	nts		3	14	0.020

**G type**	5S rDNA		2	2	0.002
*P. polymerus*	120 pb	3	1	2	0.006
	nts		1	2	0.001

n: sample size; s: number of segregating (polymorphic) sites; h: number of haplotypes; π: nucleotide diversity

Sequence divergence was examined separately for the 5S and NTS regions. Values of nucleotide diversity for the different 5S rDNA types for each species were higher in the spacer region than in the 5S (Table 1), except for the D and G types and for sequences of *P. pollicipes *F type. Estimates of evolutionary mean distances within types were relatively small (0.004-0.039) (Table 2), emphasising the low value of the sequences of the A type with respect to those of E and F types. These three types are not as biased by sample size as the others.

**Table 2:** Estimates of average evolutionary divergence over sequence pairs within types

Type	d	S.E.
A	0.009	0.003
B	0.031	0.011
C	0.039	0.013
D	0.023	0.012
E	0.034	0.010
F	0.015	0.005
G	0.004	0.004

d: distance within type; S.E: standard error

### Phylogenetic analysis

**Figure 1:**
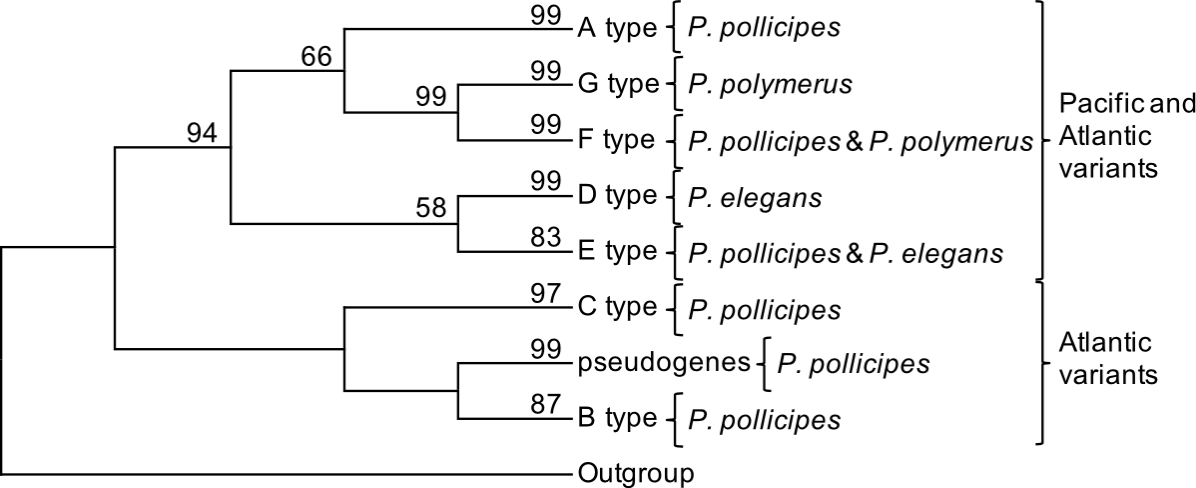
**A maximun parsimony (MP) tree**. Numbers on the tree correspond to nonparametric bootstrap supports (1000 replicates) and they are reported only for nodes with values ≥50. Outgroup species, *Artemia salina*, *Asellus aquaticus *and *Lepas anatifera *correspond to accession numbers: Y00128; X14815; AJ243001; FR832613; FR832614.

**Figure 2:**
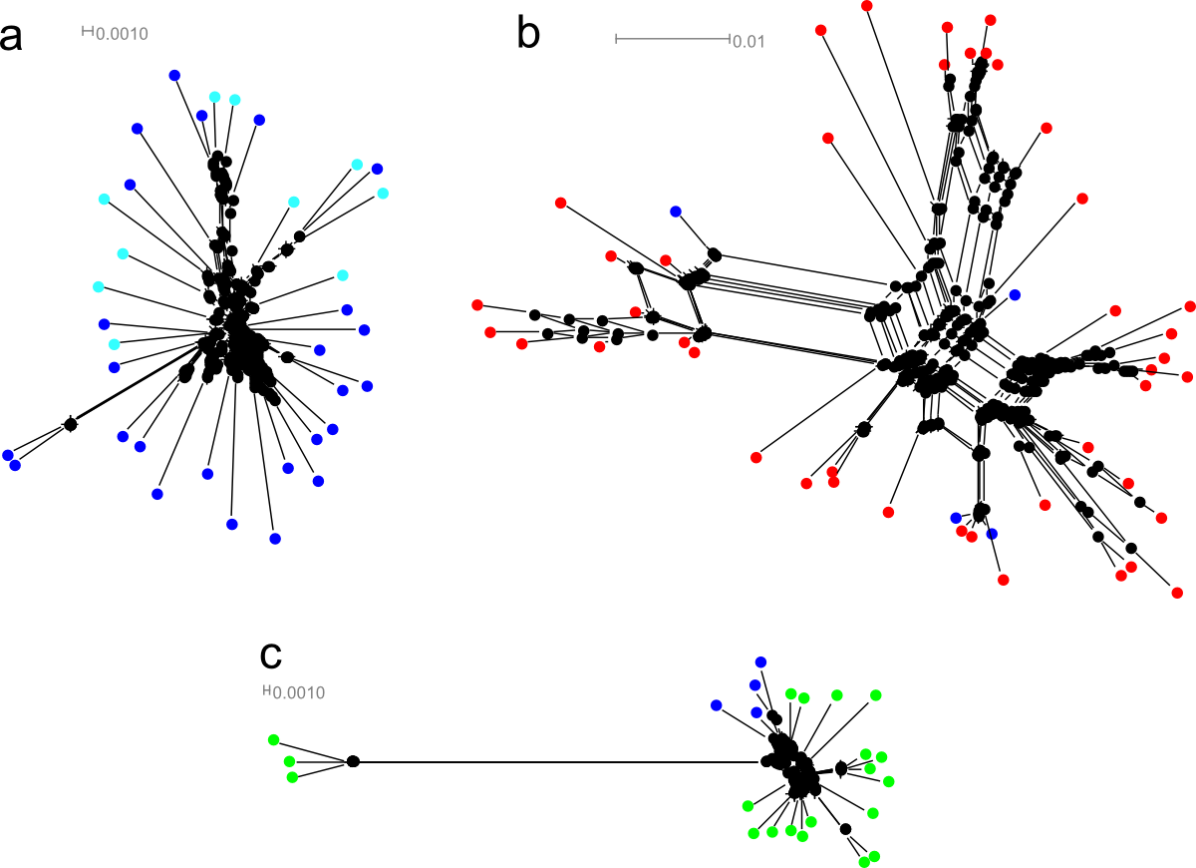
**Phylogenetic networks of 5S rDNA constructed using the neighbor net algorithm**. *Pollicipes *species are shown in different colors: *P. pollicipes *in blue, *P. elegans *in red and *P. polymerus *in green; (a) Network of A type: sequences of Galician localities are displayed in dark blue and Morocco in light blue; (b) Network of E type; (c) Network of F and G types.

Despite the length variation of the sequences, we were able to perform a blastn among them since there were some regions of similarity (see Additional File 3, Table S2). An MP tree (Figure 1) was calculated implementing the "using all sites" option in order to show the evolution of different variants. On the other hand, the networks created for each of the most frequent types of sequence (A, E and, F-G) (Figure 2) did not show a clear clustering by species. The network for A type sequences did not detect any association between different localities. Similarly, the network for the E type did not reveal any pattern of clustering for the two species that belong to this group of sequences. The E type included 47 sequences: 43 sequences of *P. elegans *and 4 sequences of *P. pollicipes*. The F type also included two species: 17 sequences of *P. polymerus *and 4 sequences of *P. pollicipes*. Sequences of F and G types could be aligned because of their similarity and in this way a network linking both them could be built. The advantage of network methods is that give easy-to grasp representation of the considerable noise in the data.

The resulting topology of the maximum likelihood trees using 5S + NTS sequences of the three main variants showed an among-species gene clustering pattern supported by high bootstraps (see Additional File 4, Figure S3). Phylogenetic analysis of the same data set with the NJ algorithm gave essentially the same topology as that obtained from the ML tree (data not shown).

### Upstream and downstream elements

As the spacer regions contain some conserved elements that may be involved in 5S transcription, the 78 nt upstream from the transcription start site of 5S rDNA of *Pollicipes *species were arranged together. These regions formed the terminal region of each NTS. A search of upstream sequences revealed a conserved AT rich region at about -25 nt from the 5S rDNA transcription start site (see Additional File 4, Figure S4) in three groups of sequences: B, C and F. Another conserved region (CGGCCACCGGC) was identified at positions -24 to -14 nt from the 5S rDNA transcription start site. All sequence types except B, C and F displayed this region. These were the same groups in which the AT-rich region was found. Finally, a TTC stretch located at -7 nt was also identified.

Another clear disparity between the 5S rDNA types was the number of thymidine residues located in the T-rich region, five in the A, B, C, F and G types and four in the D and E types. These repeated sequences corresponded to transcriptional terminators [20,21].

### Internal regulatory regions

**Figure 3:**
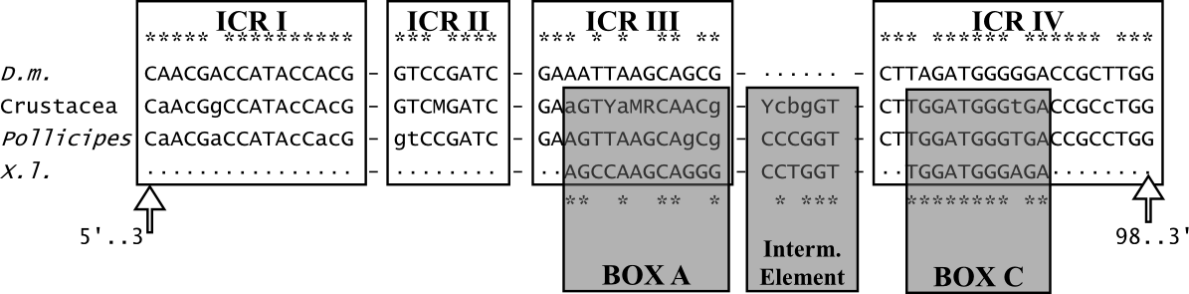
**Schematic comparison of the control elements involved in the transcription of the 5S rDNA**. Sequences up and down represent the internal control regions (ICRs) and sequence elements of *D. melanogaster *(*D. m*) and *X. laevis *(*X. l*), while sequences in the middle represent the consensus crustacea and G. *Pollicipes *orthologues. Similarities respect to the consensus sequences are denoted by asterisk (*).

The 5S internal control regions (ICRs) were compared to those of *Drosophila melanogaster *described by Sharp and Garcia [22]. As some ICRs coincided with the primer annealing regions, only sequences classified as b or c were included in the *Pollicipes *ICRs analysis, in addition to other sequences from other crustaceans available from EMBL/GenBank/DDBJ: *Parhyale hawaiensis *[FN434137]; *Proasellus coxalis *[Y14281]; *Asellus aquaticus *[AJ243001] *Calanus finmarchicus *[X06056] and *Artemia sp*. [X14815; V00086; M16191; Y00128; X14816; X14817]. The consensus internal regulatory regions of *Pollicipes *and the other crustaceans are shown in Figure 3. The four ICRs involved in the transcription of 5S rDNA [22] were identified in the 5S sequences. Thus in *Pollicipes *consensus positions 3-18, 37-44, 48-61, and 78-98 were very similar to their orthologs in *D. melanogaster *(16/16; 8/8; 13/14 and 18/21 matches respectively). We also identified the sequence elements described in *Xenopus laevis *[23] that are functionally equivalent to the ICRs: positions 50-61 (box A), 67-72 (intermediate element), and 80-90 (box C) which also displayed a high degree of similarity (9/12; 5/6; 10/11 matches respectively).

### 5S predicted secondary structures

**Figure 4:**
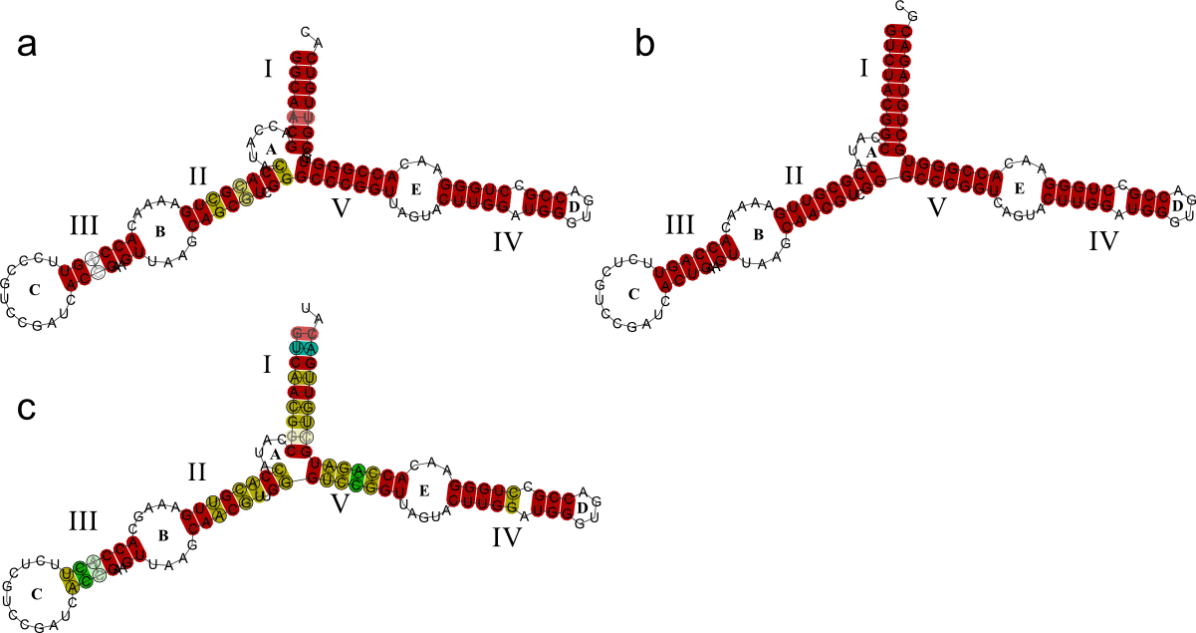
**Predicted consensus secondary structures of the 5S rRNA**. Helices are named with roman numbers, and loops with letters, following Barciszenwska *et al.*, (2000). Red indicates one type of base pair, ochre two types of base pairs, and pale colors indicate pairs that cannot be formed by all sequences. (a) Type I *Pollicipes*. (b) Type II *Pollicipes *(c) Consensus of crustacea.

Sequences included in the secondary structure prediction were those classified as b or c. All sequences were folded (see Additional File 4, Figure S5). We also obtained the consensus secondary structures, two putative types of structures for *Pollicipes *and another for other crustaceans used in this study (Figure 4). In agreement with Delihas and Andersen [24] the 5' ends were purines whereas the 3' ends were pirimidines. Lengths of helix I were 7 nt (type I) and 9 nt (type II and Crustacea). Helix II has a length of eight nt and a looped-out residue at position 63 that is a C (characteristic of metazoans); the two base-pairs that follow this residue are C-G in the general structure, consistent with metazoan and plant 5S rRNA. The two positions 49 and 50 are also flanked by G-C base-pairs on both sides. In the loop bound by helix III there are twelve nucleotides, and the purine at position 37 is a G in metazoans. The alternative structure can be observed in helix IV (as shown in [24]). This helical model includes a C-A mispairing and an increased content of non-canonical base-pairs, G-U. The C - loop is formed by 12 bp, the hairpin E - loop displays the AGUA motif and the terminal loop contains the conserved G-U-G-A motif.

## Discussion

Most of the sequences of *Pollicipes *analyzed in the present study, except the pseudogenes found in *P. pollicipes*, might be functional genes because they possess the necessary elements for gene expression, viz the presence of control elements in the NTS, the poly-T tail at the 3' end of the transcribing region, and conserved ICRs that function as internal promoters of the gene. Until recently it was thought that the NTS had no function, but studies of deletion mutants have shown that upstream control elements are required for the expression of 5S rDNA genes [4]. The NTS minimun average size was 83 bp. This size agrees with Martins and Galetti [25], who proposed that an NTS of 60-80 bp can represent the minimum size for the organization of this rDNA in the genome. Although in general 5S displays a high degree of conservation among species and variants, we found some nucleotide substitutions in *Pollicipes *spp. In the comparisons with *D. melanogaster*, the ICR I and ICR II regions were the most conserved. Furthermore, the proportion of conserved nucleotide positions in *Pollicipes *spp. is higher than those obtained for razor clams [8] and mussels [26] which is not surprising since *Pollicipes *barnacles and *D. melanogaster *belong to the Arthropoda. The degree of conservation of internal control elements in the 27 crustacean sequences was 10/16 matches within ICR I, 6/8 matches within ICR II, 8/14 matches within ICR III, and 19/21 matches within ICR IV. Many nucleotide substitutions in ICR III were unique for *Artemia *spp. The highest degree of conservation was in *Pollicipes *spp. (11/16, 6/8, 12/14 and 21/21 matches respectively) (see Additional File 4, Figure S6). The poly-T tail transcription termination signal of 5S rDNA has been studied in several organisms and seems to be quite conserved. It is part of a transcribed 15-16 nucleotide segment specific to the 5S rRNA precursor. The 135-nucleotide primary transcript was identified in *D. melanogaster *by in vitro transcription and 3'-processed to yield the approximately 126-nucleotide pre' 5S species and the 120-nucleotide mature-size 5S rRNA [22]. The analysis of upstream sequences of 5S from genus *Pollicipes *revealed a putative regulatory region, a TATA - like control element, located around positions -30 to -25 as observed in several fish species [21] and in razor clams [8]. This region, together with RNA pol II-like transcriptional factors, may be involved in RNA pol III transcription [27]. The high degree of conservation of TATA-like sequence positions in all organisms examined to date (e.g.. elasmobranch fishes, [21]) suggests a shared structural pattern. However, in our case, many sequences did not show the TATA-like motif. The fact that certain conserved regions are associated with a specific variant could be related to a differential expression throughout development as seen in *Xenopus *[28]. Furthermore, we found a TTC sequence, as previously observed in the silkworm *Bombyx mori *[29].

The predicted secondary structure of all 5S sequences analysed in this work consists of five helices, two hairpin loops, two internal loops and a hinge region. This structure is consistent with the general eukaryotic 5S rRNA structure [24,30] and with that obtained for *A. salina *[31]. According to Smirnov [32], helix I is potentially important for RNA-protein recognition and helix III seems to be associated with the integration of 5S rRNA into the large ribosome subunit. Helix IV and the terminal loop are responsible for the interaction of 5S rRNA with 23S rRNA and are involved in the integration of the large subunit RNA component. Helix IV was conserved in all the predicted consensus secondary structures. However, although helix II was conserved in the comparison with the one from *A. salina*, a nucleotide substitution (G/A) in position 61 was found in some sequences. The ability of the sequence to adopt a correct consensus secondary structure can be used to discriminate between genes and pseudogenes [33]. In this way, the putative pseudogenes of 123 bp did not fold.

In recent studies of molecular organization and evolution of 5S rDNA, several classes of 5S rDNA have been described, for example, in several species of fish [21], in razor shells [5], and in mussel species [34]. The number of these different classes of 5S rDNA is low compared with the eight different types (per species) found in filamentous fungi [6]. In this study we obtained several different classes of 5S rDNA which cluster into seven types (this number being the maximum of different variants found in animals). In these studies cited above, the main difference between classes of 5S rDNA is the length of the NTS types. In some cases the nucleotide sequence of the transcribing region also varies. In the case of *Pollicipes *there is no association between NTS and 5S.

In related species there is greater similarity among repeat units within the same cluster than among repeat units of different clusters; furthermore, similarity among repeat units within the same clusters from different related species is higher than among different clusters in the same species. According to this, some authors as Martins and Galetti [35] have suggested that different 5S rDNA loci evolve independently. We have found sequences that show a greater similarity in 5S rDNA units within a specific type between two species than between two types in the same species. Other studies have reported that the two types of 5S rDNA are not in separate clusters, since different variants have been found in tandem in the same clone [25,36]. Similarily, we found that sequences belonging to different types were organized in tandem. We sequenced 9 dimers E-E, 2 dimers F-F, one dimer D-E, another dimer F-G, one trimer E-D-E and another 2 dimers that consisted of C type monomers linked to putative pseudogenes. By gene conversion, dimers and trimers, i.e tandem repeat units, should be composed of the same variants. However, we observed that this is not always the case. This tandem organization might therefore be caused by one or both of two reasons: 1) a variant may have recently been transposed, or 2) the unit of homogenization consists of dimers or trimers. Although these variants are in tandem, the repeats could be dispersed throughout the genome. At this moment, the attempts to locate these loci on metaphase chromosomes, by fluorescent in situ hybridization (FISH), have been unsuccessful.

The evolution of ribosomal gene families has traditionally been explained by the model of concerted evolution, which proposes that all members of a gene family are assumed to evolve in a concerted manner rather than independently, and a mutation occurring in a repeat spreads through all the member genes by repeated occurrence of unequal crossover or gene conversion [10]. Therefore, sequence similarity is greater within a species than among related species [21]. However, previous studies have shown that multigene families could be evolving under the birth-and-death model. Under this model new genes are created by gene duplication, and some duplicated genes are maintained in the genome for a long time, whereas others are deleted or become non-functional through deleterious mutations [10]. Thus, according to the data (Figure 1) B and C variants could have originated after the colonization of the Atlantic ocean by *P. pollicipes*, whereas variants of the A, D, E, F, and G types are maintained in the three species so that their origin variant may have been present in the species' common ancestor. The case of ribosomal DNA could be more complex and involve a combined effect of concerted and birth-and-death evolution [3,34]. Our data did not reveal a clustering by species. There were no fixed differences among species and low levels of nucleotide variation within the 5S region. However, divergence was observed among NTSs from different units. Taken together, these observations highlight the importance of purifying selection over the functional regions.

We found two putative pseudogenes in *P. pollicipes*. The presence of 5S rDNA truncated pseudogenes has also been described in other species, including humans [37], fishes [38], and filamentous fungi [6]. As pointed out by Rooney and Ward [6], the truncated sequences are believed to be pseudogenes because their lack of an intact transcribing sequence effectively destroys the secondary structure of the 5S rRNA molecule that they would have otherwise encoded. The presence of pseudogenes in a multigene family strongly suggests that the family evolves under a birth-and-death process [39-41]. According to this model, a multigene family can expand as a consequence of gene duplication and contract because of gene loss (e.g. as a result of unequal crossover). Eventually, distinct gene copies accumulate differences, leading some of them to degenerate into pseudogenes [6]. Under a birth-and-death process, the 5S rDNA multigene family is expected to show several variants, and the phylogenetic analyses of the genes of several closely related species will not show a within-species clustering pattern, but they should cluster according to their sequence similarities [3,5,7]. This agrees with the pattern obtained in the phylogenies and networks where sequences of 5S rDNA belonging to different *Pollicipes *species clustered together. In some species, 5S rDNA are dispersed throughout the genome, as in *Schizosaccharomyces pombe *[42]. The dispersed gene organization apparently facilitates birth-and-death evolution wherein rRNA genes diverge from one another, some being unique to a given species, others shared among species [41].

## Conclusions

Although more experimental work is needed to reveal the number 5S rDNA variants within a genome, our study has provided new and interesting insights into the genome organization of 5S rDNA in barnacles, and is the first to demonstrate that crustaceans can posses different size variants of 5S rDNA arrays carrying a distinct NTS spacer. We found up to seven different types of 5S rDNA based on the analysis of the NTS region. Five different units of 5S rDNA were characterized in *P. pollicipes *and two in *P. elegans *and *P. polymerus*. Our results demonstrate that the 5S rDNA of the genus *Pollicipes *is organized in tandem repeats of different sizes, although dispersed units can be present in the genome. In short, we found (1) up to seven 5S rDNA types in *Pollicipes *spp., (2) an interspecies clustering of *Pollicipes *5S rDNA variants, (3) identical variants shared among species and unique variants that are species specific, (4) a lack of homogenization between spacer sequences of different types, and (5) two pseudogenes.

We conclude that *Pollicipes *5S rDNA is subjected to birth-and-death evolution with strong purifying selection that explains the low levels of variation found in the 5S and the extant variation of NTS sequences. This evolutionary mechanism described in fungi and bivalve molluscs appears to be applicable to other organisms. Moreover, further studies on crustacean species are needed to improve our knowledge of 5S rDNA organization and evolution in this group of organisms.

## Methods

Genetic analyses were conducted on three species of the genus *Pollicipes*. Samples of the Atlantic species *P. pollicipes *were obtained from different localities of Galicia (Spain) and a Morocco market (Table 3). *P. elegans *specimens were collected in northern Peru, and *P. polymerus *in Olympic National Park (Washington, USA). Accession numbers are [EMBL/GenBank/DDBJ: FR831801-FR831899] (see Additional File 2,Table S1). Pieces of foot muscle were excised and preserved in absolute ethanol.

**Table 3:** Samples of barnacles used in this study

Species	Locality	Coordinates	no	Species code
	Ortigueira (Galician, Spain)	43° 44' 30" N 7° 56' 58" W	3	Po-Ort
	Golfo Ártabro (Galician, Spain)	43° 24' 5" N 8° 19' 53" W	6	Po-Art
*P. pollicipes*	Bens (Galician, Spain)	43° 21' 38" N 8° 27' 29" W	4	Po-Ben
	Balcobo (Galician, Spain)	43° 19' 3" N 8° 31' 49" W	6	Po-Bal
	Local market (Morocco)	32° 32' 22" N 9° 17' 15" W	3	Po-Mar

	Lobos de Afuera island (Peru)	6° 58' 12" S 80° 42' 2" W	3	El-Afu
*P. elegans*	Lobos de Tierra island (Peru)	6° 26' 14" S 80° 50' 55" W	7	El-Tie

*P. polymerus*	Olympic National Park (Washington, USA)	48° 23' 13" N 124° 44' 4" W	6	Py-Oly

no, number of individuals

The NucleoSpin Tissue kit (Macherey-Nagel and Co.) was used to extract genomic DNA from foot tissue. The 5S rDNA sequences from each genomic DNA were amplified by PCR. Three pairs of primers were used for this analysis. The first one pair was 5S-Univ-F and 5S-Univ-R [5]. Two new and more specific pairs were designed from *P. pollicipes *5S sequences, available after amplification with 5S-Univ-F and 5S-Univ-R. These new pairs of primers were 5S-Poll-F (5'-TCC GAT CAC CGA AGT TAA GC-3') and 5S-Poll-R (5'-ACC GGT GTT TTC AAC GTG AT-3'), and 5S-Poll-F and 5S-Poll-R2 (5'-ACT GGT GTT TTC AAC GTG GT-3'). These designed primers have opposite orientations, are separated by 5 bp and anneal at positions 13-32 y 38-57 of the 5S transcribing region. They were designed for the amplification of one unit of any tandemly arranged 5S rDNA in the genus *Pollicipes*.

PCRs were carried out in a BIORAD My Cycler tm thermocycler using a reaction volume of 25 μl containing ~ 25 ng genomic DNA, 200 μM each dNTP (Roche Diagnostics), 0.5 μM each primer, 0.625 U Taq DNA polymerase (Roche Diagnostics), the buffer recommended by the polymerase supplier and 2.5 mM MgCl_2_. Thermocycling conditions were 5 min at 95°C; 35 cycles of 30 s at 95°C, 30 s at 50°C, and 30 s at 72°C; and a final 5 min extension at 72°C. A negative control was also included to test for any contamination. The PCR products were resolved in 1.5% agarose gel, and visualized after ethidium bromide staining via ultraviolet trans-illumination.

The PCR-generated 5S rDNA fragments were cloned in the pSC-A-amp/kan PCR Cloning Vector (StrataGene), and used to transform *E. coli *competent cells. A subset of transformant colonies from each cloning reaction was analyzed by PCR in order to check the insert size. A QiaPrep Spin Miniprep Kit (Qiagen) was used to purify the plasmids. Sequencing reactions were carried out using both M13 Forward and M13 Reverse primers in a capillary DNA sequencer (3130xl Genetic Analysis System, Applied Biosystems).

### Bioinformatics analysis

The quality of the electropherograms was checked in BioEdit 7.0.9.0. [43]. The BLAST 2 Sequence Tool [44] was used to compare the ends of both forward and reverse sequences obtained from each clone. These sequences were overlapped by hand. For sequence alignment we used ClustalX [45]. Because length variation is a problem when performing alignments, sequences had to be grouped separately, according to similarity. Clustering was performed with the TGI Clustering Tools developed at TIGR (http://compbio.dfci.harvard.edu/tgi/software/). We also performed a statistical evaluation of the local similarities among spacer types or assembled clusters in a local BLAST in BioEdit 7.0.9.0. [43]. The BLAST 2 Sequences Tool was employed to evaluate the local similarities between pairs of sequences that belong to different types.

The number of polymorphic sites, the number of haplotypes and the nucleotide diversity were calculated from DnaSP 5.00.04 [46]. All nucleotide sequence divergence analyses were conducted using the Maximum Composite Likelihood method in MEGA 4.0.2. [47]. All positions containing gaps and missing data were eliminated from the dataset after selecting the complete deletion option. Standard errors were calculated by the bootstrap option with 1000 replicates.

In order to search for putative regulatory conserved elements, we analysed sequences upstream and downstream of the 5S region. Searches were performed within the first 78-120 nt upstream and downstream of the DNA transcribing region. Some putative 5S rDNA transcriptional regulatory motifs were identified by the TOUCAN workbench [48] establishing a comparison with reference sequences from the *Drosophila *Eukaryotic Promoter (EPD) and JASPAR database, and others were manually compared with published regulatory elements. The 5S sequences were folded into the RNA alifold web server [49] applying constraints (see Additional File 5) to obtain the predicted consensus secondary structures.

*Pollicipes *5S rDNA sequences were subjected to a neighbor-net analysis [50] implemented in the SplitsTree 4 package [51] using GTR distances [52]. A maximun parsimony (MP) tree was built using MEGA 4.0.2 [47], selecting the "use all sites" option. Bootstrap resampling was applied to assess support for individual nodes using 1000 replicates. Additionally, maximum likelihood (ML) phylogenetic relationships among 5S rDNA sequences were established using the PALM web server [53]. The reliability of the topologies was tested by the bootstrap procedure [54] with 100 replicates. Modeltest 3.7 software [55] was employed to determine the best-fit model of nucleotide substitution, applying the Akaike information criterion (AIC).

## Authors' contributions

AP obtained the sequence data, carried out the genetic analyses and drafted the manuscript. DS and FRF helped in the DNA cloning and sequencing. AMGT and AML coordinated the study and helped to draft the manuscript. All authors read and approved the final version of manuscript.

## Supplementary Material

Additional file 1 **Figure S1: Different 5S rDNA tandem arrangements**. Drawings are done to scale. Scheme is as follows: a) last portion of the 5S (88 bp), NTS, 5S (120 pb), NTS, 5S (120 pb), NTS, and the first portion of the contiguous 5S (32 pb) for the trimer; b-g) last portion of the 5S (88 bp), NTS, 5S (120 pb), NTS, and the first portion of the contiguous 5S (32 pb) for dimers. Different colors show different types of NTS. Dotted arrows indicate putative pseudogenes. 5S, 5S rDNA gene; NTS, nontranscribed spacer. Click here for file

Additional file 2 **Table S1: Accession numbers and details of barnacle sequences used in this study**. Click here for file

Additional file 3 **Figure S2: Alignments of different types**. F and G types are aligned together. Putative pseudogenes (red) are aligned with C type sequences. Table S2: Values of similarity among NTS types. Click here for file

Additional file 4 **Figure S3: Phylogenetic relationships of 5S rDNA for the three main variants in *Pollicipes *species reconstructed by means of a maximun likelihood trees**. Numbers on nodes represent bootstrap values based on 100 replicates. (a) Phylogeny of A type reconstruted by K81uf + I + G model. (b) Phylogeny of E type reconstruted by SYM + I + G model. (c) Phylogeny of F and G types reconstruted by HKY + I + G model. In (b) and (c), asterisks indicate *P. pollicipes *sequences. Figure S4: Identified regions (not aligned) of 78 nucleotides upstream the transcriptional start site of 5S ribosomal DNA. Three conserved regions were identified. Nucleotides shaded in blue share the motif CGGCCACCGGC, those shaded in red correspond to an AT rich region that it was located about -25 bp, and the TTC sequence (shared with *Bombyx mori *silkworms) is shaded in green color. Figure S5: 5S ribosomal RNA predicted secondary structures of barnacles. Structures correspond to the b and c types sequences that excluding the primer-annealing regions. (a-h) Sequences used in the predicted consensus secondary structures type I *Pollicipes *and (i) type II *Pollicipes*. ((a) *El04Tie09b*;(b) *El03Afu02b*; (c) *Py02Oly04b*; (d) *El04Afu04b*, *El03Afu19b*, *El05Afu04b*, *El03Afu02c*, *El01Tie02b*, *El01Tie01b*; (e) *El01Tie03b*; (f) *Py08Oly03b*, *Py03Oly01b*; (g) *El03Afu07b*, *El04Tie06b*; (h) *El03Afu16b*; (i) *Po06Bal01b*, *Po06Bal02b*. Figure S6: 5S alignment. Upper line is the consensus 5S rDNA gene. White boxes represent the internal control regions (ICRs) involved in the transcription of the *D. melanogaster *5S rDNA, and grey shading areas correspond to the three sequence elements that regulate transcription activity of *X. laevis *5S rDNA (box A, intermediate element, and box C, from left to right). The b and c types sequences represent the second and third unit of the array. Click here for file

Additional file 5 **Constraints applied in RNA alifold**. Click here for file
