# Supplementary material for: Molecular organization and phylogenetic analysis of 5S rDNA in crustaceans of the genus Pollicipes reveal birth-and-death evolution and strong purifying selection
Source: BMC Evol Biol. 2011 Oct 17;11:304. doi: 10.1186/1471-2148-11-304 (PMC3215682; doi:10.1186/1471-2148-11-304)

Additional File 1, Figure S1: Different 5S rDNA tandem arrangements. Drawings are done to scale. Scheme is as follows: a) last portion of the 5S (88 bp), NTS, 5S (120 pb), NTS, 5S (120 pb), NTS, and the first portion of the contiguous 5S (32 pb) for the trimer; b-g) last portion of the 5S (88 bp), NTS, 5S (120 pb), NTS, and the first portion of the contiguous 5S (32 pb) for dimers. Different colors show different types of NTS. Dotted arrows indicate putative pseudogenes. 5S, 5S rDNA gene; NTS, nontranscribed spacer.

a)

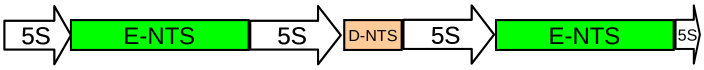

b)

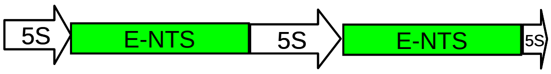

c)

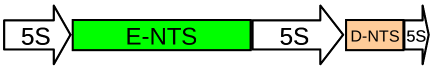

d)

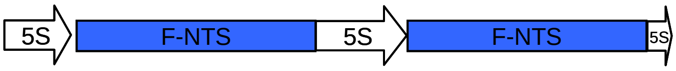

f)

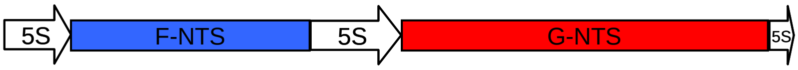

g)

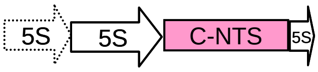

Supplement: Additional file 1 — Figure S1: Different 5S rDNA tandem arrangements. Drawings are done to scale. Scheme is as follows: a) last portion of the 5S (88 bp), NTS, 5S (120 pb), NTS, 5S (120 pb), NTS, and the first portion of the contiguous 5S (32 pb) for the trimer; b-g) last portion of the 5S (88 bp), NTS, 5S (120 pb), NTS, and the first portion of the contiguous 5S (32 pb) for dimers. Different colors show different types of NTS. Dotted arrows indicate putative pseudogenes. 5S, 5S rDNA gene; NTS, nontranscribed spacer. [file 1471-2148-11-304-S1.PDF]
