# Supplementary material for: Molecular organization and phylogenetic analysis of 5S rDNA in crustaceans of the genus Pollicipes reveal birth-and-death evolution and strong purifying selection
Source: BMC Evol Biol. 2011 Oct 17;11:304. doi: 10.1186/1471-2148-11-304 (PMC3215682; doi:10.1186/1471-2148-11-304)
Supplement: Additional file 3 — Figure S2: Alignments of different types. F and G types are aligned together. Putative pseudogenes (red) are aligned with C type sequences. Table S2: Values of similarity among NTS types. [file 1471-2148-11-304-S3.PDF]

Additional File 3, Figure S2: Alignments of different types. F and G types are aligned together. Putative pseudogenes (red) are aligned with C type sequences.

A type

```
                           ....|....| ....|....| ....|....| ....|....| ....|....| ....|....| ....|....| ....|....| ....|....| ....|....| ....|....| ....|....|
                                  10         20         30         40         50         60         70         80         90        100        110
120
Po12Bal02|FR831833         GGCAACGACC ATACCACGTT GAAAACACCA GTTCCCGTCC GATCACCGAA GTTAAGCAGC ATCGGGCCCG GTTAGTACTT GGATGGGTGA CCGCCTGGGA ACACCGGGTG CCGTTGTCAC
Po36Ort01|FR831805         GGCAACGACC ATACCACGTT GAAAACACCA GTTCCCGTCC GATCACCGAA GTTAAGCAGC ATCGGGCCCG GTTAGTACTT GGATGGGTGA CCGCCTGGGA ACACCGGGTG CCGTTGTCAC
Po06Ben02|FR831823         GGCAACGACC ATATCACGTT GAAAACACCG GTTCCCGTCC GATCACCGAA GTTAAGCAGC ATCGGGCCCG GTTAGTACTT GGATGGGTGA CCGCCTGGGA ACACCGGGTG CCGTTGTCAC
Po06Ben20|FR831824         GGCAGCGACC ATATCACGTT GAAAACACCG GTTCCCGTCC GATCACCGAA GTTAAGCAGC ATCGGGCCCG GTTAGTACTT GGATGGGTGA CCGCCTGGGA ACACCGGGTG CCGTTGTCAC
Po16Ort05|FR831804         GGCAACGACC ATACCACGTT GAAAACACCA GTTCCCGTCC GATCACCGAA GTTAAGCAGC ATCGGGCCCG GTTAGTACTT GGATGGGTGA CCGCCTGGGA ACACCGGGTG CCGTTGTCAC
Po19Art02|FR831814         GGCAACGACC ATACCACGTT GAAAACACCA GTTCCCGTCC GATCACCGAA GTTAAGCAGC ATCGGGCCCG GTTAGTACTT GGATGGGTGA CCGCCTGGGA ACACCGGGTG CCGTTGTCAC
Po19Art04|FR831815         GGCAACGACC ATACCACGTT GAAAACACCA GTTCCCGTCC GATCACCGAA GTTAAGCAGC ATCGGGCCCG GTTAGTACTT GGATGGGTGA CCGCCTGGGA ACACCGGGTG CCGTTGTCAC
Po36Ort05|FR831806         GGCAACGACC ATACCACGTT GAAAACACCA GTTCCCGTCC GATCACCGAA GTTAAGCAGC ATCGGGCCCG GTTAGTACTT GGATGGGTGA CCGCCTGGGA ACACCGGGTG CCGTTGTCAC
Po11Art01|FR831811         GGCAACGACC ATACCACGTT GAAAACACCA GTTCCCGTCC GATCACCGAA GTTAAGCAGC ATCGGGCCCG GTTAGTACTT GGATGGGTGA CCGCCTGGGA ACACTGGGTG CCGTTGTCAC
Po08Art01|FR831810         GGCAACGACC ATACCACGTT GAAAACACCA GTTCCCGTCC GATCACCGAA GTTAAGCAGC ATCGGGCCCG GTTAGTACTT GGATGGGTGA CCGCCTGGGA ACACCGGGTG CCGTTGTCAC
Po06Mar06|FR831843         GGCAACGACC ATACCACGTT GAAAACACCA GTTCCCGTCC GATCACCGAA GTTAAGCAGC ATCGGGCCCG GTTAGTACTT GGATGGGTGA CCGCCTGGGA ACACCGGGTG CCGTTGTCAC
Po07Ben04|FR831826         GGCAACGACC ATACCACGTT GAAAACACCA GTTCCCGTCC GATCACCGAA GTTAAGCAGC ATCGGGCCCG GTTAGTACTT GGATGGGTGA CCGCCTGGGA ACACCGGGTG CCGTTGTCAC
Po19Art05|FR831816         GGCAACGACC ATACCACGTT GAAAACACCA GTTCCCGTCC GATCACCGAA GTTAAGCAGC ATCGGGCCCG GTTAGTACTT GGATGGGTGA CCGCCTGGGA ACACCGGGTG CCGTTGTCAC
Po03Ort02|FR831801         GGCAACGACC ATACCACGTT GAAAACACCA GTTCCCGTCC GATCACCGAA GTTAAGCAGC ATCGGGTCCG GTTAGTACTT GGATGGGTGA CCGCCTGGGA ACACCGGGTG CCGTTGTCAC
Po07Ben02|FR831825         GGCAACGACC ATACCACGTT GAAAACACCA GTTCCCGTCC GATCACCGAA GTTAAGCAGC ATCGGGCCCG GTTAGTACTT GGATGGGTGA CCGCCTGGGA ACACCGGGTG CCGTTGTCAC
Po16Ort04|FR831803         GGCAACGACC ATACCACGTT GAAAACACCA GTTCCCGTCC GATCACCGAA GTTAAGCAGC ATCGGGCCCG GTTAGTACTT GGATGGGTGA CCGCCTGGGA ACACCGGGTG CCGTTGTCAC
Po06Ben01|FR831822         GGCAACGACC ATATCACGTT GAAAACACCG GTTCCCGTCC GATCACCGAA GTTAAGCAGC ATCGGGCCCG GTTAGTACTT GGATGGGTGA CCGCCTGGGA ACACCGGGTG CCGTTGTCAC
Po16Ort01|FR831802         GGCAACGACC ATACCACGTT GAAAACACCA GTTCCCGTCC GATCACCGAA GTTAAGCAGC ATCGGGCCCG GTTAGTACTT GGATGGGTGA CCGCCTGGGA ACACCGGGTG CCGTTGTCAC
Po01Ben02|FR831818         GGCAACGACC ATACCACGTT GAAAACACCA GTTCCCGTCC GATCACCGAA GTTAAGCAGC ATCGGGCCCG GTTAGTACTT GGATGGGTGA CCGCCTGGGA ACACCGGGTG CCGTTGTCAC
Po01Ben01|FR831817         GGCAACGACC ATACCACGTT GAAAACACCA GTTCCCGTCC GATCACCGAA GTTAAGCAGC ATCGGGCCCG GTTAGTACTT GGATGGGTGA CCGCCTGGGA ACACCGGGTG CCGTTGTCAC
Po09Mar20|FR831849         GGCAACGACC ATACCACGTT GAAAACACCA GTTCCCGTCC GATCACCGAA GTTAAGCAGC ATCGGGCCCG GTTAGTACTT GGATGGGTGA CCGCCTGGGA ACACCGGGTG CCGTTGTCAC
Po08Mar05|FR831845         GGCAACGACC ATACCACGTT GAAAACACCA GTTCCCGTCC GATCACCGAA GTTAAGCAGC ATCGGGCCCG GTTAGTACTT GGATGGGTGA CCGCCTGGGA ACACCGGGTG CCGTTGTCAC
Po09Mar02|FR831848         GGCAACGACC ATACCACGTT GAAAACACCA GTTCCCGTCC GATCACCGAA GTTAAGCAGC ATCGGGCCCG GTTAGTACTT GGATGGGTGA CCGCCTGGGA ACACCGGGTG CCGTTGTCAC
Po06Mar04|FR831841         GGCAACGACC ATACCACGTT GAAAACACCA GTTCCCGTCC GATCACCGAA GTTAAGCAGC ATCGGGCCCG GTTAGTACTT GGATGGGTGA CCGCCTGGGA ACACCGGGTG CCGTTGTCAC
Po14Bal02|FR831835         GGCAACGACC ATACCACGTT GAAAACACCA GTTCCCGTCC GATCACCGAA GTTAAGCAGC ATCGGGCCCG GTTAGTACTT GGATGGGTGA CCGCCTGGGA ACACCGGGTG CCGTTGTCAC
Po08Mar06|FR831846         GGCAACGACC ATACCACGTT GAAAACACCA GTTCCCGTCC GATCACCGAA GTTAAGCAGC ATCGGGCCCG GTTAGTACTT GGATGGGTGA CCGCCTGGGA ACACCGGGTG CCGTTGTCAC
Po08Mar07|FR831847         GGCAACGACC ATACCACGTT GAAAACACCA GTTCCCGTCC GATCACCGAA GTTAAGCAGC ATCGGGCCCG GTTAGTACTT GGATGGGTGA CCGCCTGGGA ACACCGGGTG CCGTTGTCAC
Po08Mar04|FR831844         GGCAACGACC ATACCACGTT GAAAACACCA GTTCCCGTCC GATCACCGAA GTTAAGCAGC ATCGGGCCCG GTTAGTACTT GGATGGGTGA CCGCCTGGGA ACACCGGGTG CCGTTGTCAC
Po11Bal01|FR831831         GGCAACGACC ATACCACGTT GAAAACACCA GTTCCCGTCC GATCACCGAA GTTAAGCAGC ATCGGGCCCG GTTAGTACTT GGATGGGTGA CCGCCTGGGA ACACCGGGTG CCGTTGTCAC
Po11Bal02|FR831832         GGCAACGACC ATACCACGTT GAAAACACCA GTTCCCGTCC GATCACCGAA GTTAAGCAGC ATCGGGCCCG GTTAGTACTT GGATGGGTGA CCGCCTGGGA ACACCGGGTG CCGTTGTCAC
Po06Mar02|FR831840         GGCAACGACC ATACCACGTT GAAAACACCA GTTCCCGTCC GATCACCGAA GTTAAGCAGC ATCGGGCCCG GTTAGTACTT GGATGGGTGA CCGCCTGGGA ACACCGGGTG CCGTTGTCAC
Po06Mar05|FR831842         GGCAACGACC ATACCACGTT GAAAACACCA GTTCCCGTCC GATCACCGAA GTTAAGCAGC ATCGGGCCCG GTTAGTACTT GGATGGGTGA CCGCCTGGGA ACACCGGGTG CCGTTGTCAC
Po36Ort07|FR831807         GGCAACGACC ATACCACGTT GAAAACACCA GTTCCCGTCC GATCACCGAA GTTAAGCAGC ATCGGGCCCG GTTAGTACTT GGATGGGTGA CCGCCTGGGA ACACCGGGTG CCGTTGTCAC
Po19Art01|FR831813         GGCAACGACC ATACCACGTT GAAAACACCA GTTCCCGTCC GATCACCGAA GTTAAGCAGC ATCGGGCCCG GTTAGTACTT GGATGGGTGA CCGCCTGGGA ACACCGGGTG CCGTTGTCAC
Po07Ben05|FR831827         GGCAACGACC ATACCACGTT GAAAACACCA GTTCCCGTCC GATCACCGAA GTTAAGCAGC ATCGGGCCCG GTTAGTACTT GGATGGGTGA CCGCCTGGGA ACACCGGGTG CCGTTGTCAC
Clustal Consensus          **** ***** *** ****** *********  ********** ********** ********** ****** *** ********** ********** ********** **** ***** **********

                           ....|....| ....|....| ....|....| ....|....| ....|....| ....|....| ....|....| ....|....| ....|....| ....|....| ....|....| ....|....|
                                 130        140        150        160        170        180        190        200        210        220        230
240
Po12Bal02|FR831833         TTTTTCCGCC AGGTGATTTT TTT--CTTTT TGCCCCGACG ACCGTAAGTC ATAGCCGGGG CTGGGGGAAC TTACTTTTGG CCTAAGGCCA GGCAAAAGGC TACGTTTAGC CGGCTTGTGC
Po36Ort01|FR831805         TTTTTCCGCC AGGTGATTTT TTTT-CTTTT TGCCCCGGCG ACCGTAAGTC ATAGCCGGGG CTGGGGGAAC TTACTTTTGG CCTAAGGCCA GGCAAAAGGC TACGCTAAGC CGGCTTGTGC
Po06Ben02|FR831823         TTTTTCCGCC AGGTGATTTT TTT--CTTTT TGCCCCGGCG ACCGTAAGTC ATAGCCGGGG CTGGGGGAAC TTACTTTTGG CCTAAGGCCA GGCAAAAGGC TACGTTAAGC CGGCTTGTGC
Po06Ben20|FR831824         TTTTTCCGCC AGGTGATTTT TTT--CTTTT TGCCCCGGCG ACCGTAAGTC ATAGCCGGGG CTGGGGGAAC TTACTTTTGG CCTAAGGCCA GGCAAAAGGC TACGTTAAGC CGGCTTGTGC
Po16Ort05|FR831804         TTTTTCCGCC AGGTGATTTT TTT--CTTTT TGCCCCGGCG ACCGTAAGTC ATAGCCGGGG CTGGGGGAAC TTACTTTTGG CCTAAGGCCA GGCAAAAGGC TACGTTAAGC CGGCTTGTGC
Po19Art02|FR831814         TTTTTCCGCC AGGTGATTTT TTT--CTTTT TGCCCCGGCG ACCGTAAGTC ATAGCCGGGG CTGGGGGAAC TTACTTTTGG CCTAAGGCCG GGCAAAAGGC TACGTTAAGC CGGCTTGTGC
Po19Art04|FR831815         TTTTTCCGCC AGGTGATTTT TTT--CTTTT TGCCCCGGCG ACCGTAAGTC ATAGCCGGGG CTGGGGGAAC TTACTTTTGG CCTAAGGCCG GGCAAAAGGC TACGTTAAGC CGGCTTGTGC
Po36Ort05|FR831806         TTTTTCCGCC AGGTGATTTT TTT--CTTTT TGCCCCGGCG ACCGTAAGTC ATAGCCGGGG CTGGGGGAAC TTACTTTTGG CCTAAGGCCG GGCAAAAGGC TACGTTAAGC CGGCTTGTGC
Po11Art01|FR831811         TTTTTCCGCC AGGTGATTTT TTT--CTTTT TGCCCCGGCG ACCGTAAGTC ATAGCCGGGG CTGGGGGAAC TTACTTTTGG CCTAAGGCCG GGCAAAAGGC TACGTTAAGC CGGCTTGTGC
```

```
Po08Art01|FR831810    TTTTTCCGCC AGGTGATTTG TTT--CTTTT TGCCCCGGCG ACCGTAAGTC ATAGCCGGGG CTGGGGGAAC TTACTTTTGG CCTAAGGCCG GGCAAAAGGC TACGTTAAGC CGGCTTGTGC
Po06Mar06|FR831843    TTTTTCCGCC AGGTGATTTT TTT--CTTTT TGCCCCGGCG ACCGTAAGTC ATAGCCGGGG CTGGGGGAAC TTACTTTTGG CCTAAGGCCA GGCAAAAGGC TACGTTAAGC CGGCTTGTGC
Po07Ben04|FR831826    TTTTTCCGCC AGGTGATTTT TTT--CTTTT TGCCCCGGCG ACCGTAAGTC ATAGCCGGGG CTGGGGGAAC TTACTTTTGG CCTAAGGCCA GGCAAAAGGC TACGTTAAGC CGGCTTGTGC
Po19Art05|FR831816    TTTTTCCGTC AGGTGATTTT TTT--CTTTT TGCCCCGGCG ACCGTAAGTC CTAGCCGGGG CTGGGGGAGC TTACTTTTGG CCTAAGGCCA GGCAAAAGGC TACGTTAAGC CGGCTTGCGC
Po03Ort02|FR831801    TTTTTCCGCC AGGTGATTTT TTT--CTTTT TGCCCCGGCG ACCGTAAGTC ATAGCCGGGG CTGGGGGAAC TTACTTTTGG CCTAAGGCCA GGCAAAAGGC TACGTTAAGC CGGCTTGTGC
Po07Ben02|FR831825    TTTTTCCGCC AGGTGATTTT TTT--CTTTT TGCCCCGGCG ACCGTAAGTC ATAGCCGGGG CTGGGGGAAC TTACTTTTGG CCTAAGGCCG GGCAAAAGGC TACGTTAAGC CGGCTTGTGC
Po16Ort04|FR831803    TTTT-CCGCC AGGTGATTTT TT---CTTTT TGCCCCGGCG ACCGTAAGTC ATAGCCGGGG CTGGGGGAAC TTACTTTTGG CCTAAGGCCA GGCAAAAGGC TACGTTAAGC CGGCTTGCGC
Po06Ben01|FR831822    TTTTTCCGCC AGGTGATTTT TTT--CTTTT TGCCCCGGCG ACCGTAAGTC ATAGCCGGGG CTGGGGGAAC TTACTTTTGG CCTAAGGCCA GGCAAAAGGC TACGTTAAGC CGGCTTGCGC
Po16Ort01|FR831802    TTTTTCCGCC AGGTGATTTT TTT--CTTTT TGCCCCGGCG ACCGTAAGTC ATAGCCGGGG CTGGGGGAAC TTACTTTTGG CCTAAGGCCA GGCAAAAGGC TACGTTAAGC CGGCTTGCGC
Po01Ben02|FR831818    TTTTTCCGCC AGGTGATTTT TTTTTCTTTT TGCCCCGGCG ACCGTAAGTC ATAGCCGGGG CTGGGGGAAC TTACTTTTGG CCTAAGGCCA GGCAAAAGGC TACGTTAAGC CGGCTTGCGC
Po01Ben01|FR831817    TTTTTCCGCC AGGTGATTTT TTT--CTTTT TGCCCCGGCG ACCGTAAGTC ATAGCCGGGA CTGGGGGAAC CTACTTTTGG CCTAAGGCCA GGCAAAAGGC TACGTTAAGC CGGCTTGCGC
Po09Mar20|FR831849    TTTTTCCGCC AGGTGATTTT TTT--CTTTT TGCCCCGGCG ACCGTAAGTC ATAGCCGGGG CTGGGGGAGC TTACTTTTGG CCTAAGGCCA GGCAAAAGGC TACGTTAGGC CGGCTTGTGC
Po08Mar05|FR831845    TTTTTCCGCC AGGTGATTTT TTTT-CTTTT TGCCCCGGCG ACCGTAAGTC ATAGCCGGGG CTGGGGGAAC TTACTTTTGG CCTAAGGCCA GGCAAAAGGC TACGTTAAGC CGGCTTGTGC
Po09Mar02|FR831848    TTTTTCCGCC AGGTGATTTT TTT--CTTTT TGCCCCGGCG ACCGTAAGTC ATAGCCGGGG CTGGGGGAAC TTACTTTTGG CCTATGGCCA GGCAAAAGGC TACGTTAAGC CGGCTTGTGC
Po06Mar04|FR831841    TTTTTCCGCC AGGTGATTTT TTTT-CTTTT TGCCCCGGCG ACCGTAAGTC ATAGCCGGGG CTGGGGGAAC TTACTTTTGG CCTATGGCCA GGCAAAAGGC TACGTTAAGC CGGCTTGTGC
Po14Bal02|FR831835    TTTTTCCGCC AGGTGATTTT TTTT-CTTTT TGCCCCGGCG ACCGTAAGTC ATAGCCGGGG CTGGGGGAAC TTACTTTTGG CCTAAGGCCA GGCAAAAGGC TACGTTAAGC CGGCTTGTGC
Po08Mar06|FR831846    TTTTTCCGCC AGGTGATTTT TTTT-CTTTT TGCCCCGGCG ACCGTAAGTC ATAGCCGGGG CTGGGGGAAC TTACTTTTGG CCTAAGGCCA GGCAAAAGGC TACGTTAAGC CGGCTTGTGC
Po08Mar07|FR831847    TTTTTCCGCC AGGTGATTTT TTTT-CTTTT TGCCCCGGCG ACCGTAAGTC ATAGCCGGGG CTGGGGGAAC TTACTTTTGG CCTAAGGCCA GGCAAAAGGC TACGTTAAGC CGGCTTGTGC
Po08Mar04|FR831844    TTTTTCCGCC AGGTGATTTT TTT--CTTTT TGCCCCGGCG ACCGTAAGTC ATAGCCGGGG CTGGGGGAAC TTACTTTTGG CCTAAGGCCA GGCAAAAGGC TACGTTAAGC CGGCTTGTGC
Po11Bal01|FR831831    TTTTTCCGCC AGGTGATTTT TTTT-CTTTT TGCCCCGGCG ACCGTAAGTC ATAGCCGGGG CTGGGGGAAC TTACTTTTGG CCTATGGCCA GGCAAAAGGC TACGTTAAGC CGGCTTGTGC
Po11Bal02|FR831832    TTTTTCCGCC AGGTGATTTT TTTT-CTTTT TGCCCCGGCG ACCGTAAGTC ATAGCCGGGG CTGGGGGAAC TTACTTTTGG CCTATGGCCA GGCAAAAGGC TACGTTAAGC CGGCTTGTGC
Po06Mar02|FR831840    TTTTTCCGCC AGGTGATTTT TTTT-CTTTT TGCCCCGGCG ACCGTAAGTC ATAGCCGGGG CTGGGGGAAC TTACTTTTGG CCTATGGCCA GGCAAAAGGC TACGTTAAGC CGGCTTGTGC
Po06Mar05|FR831842    TTTTTCCGCC AGGTGATTTT TTTT-CTTTT TGCCCCGGCG ACCGTAAGTC ATAGCCGGGG CTGGGGGAAC TTACTTTTGG CCTAAGGCCG GGCAAAAGGC TACGTTAAGC CGGCTTGTGC
Po36Ort07|FR831807    TTTTTCCGCC AGGTGATTTT TTT--CTTTT TGCCCCGGCG ACCGTAAGTC ATAGCCGGGG CTGGGGGAAC TTACTTTTGG CCTAAGGCCG GGCAAAAGGC TACGTTAAGC CGGCTTGTGC
Po19Art01|FR831813    TTTTTCCGCC AGGTGATTTT TTT--CTTTT TGCCCCGGCG ACCGTAAGTC CTAGCCGGGG CTGGGGGAGC TTACTTTTGG CCTAAGGCCA GGCAAAAGGC TACGTTAAGC CGGCTTGCGC
Po07Ben05|FR831827    TTTTTCCGCC AGGTGATTTT TTT--CTTTT TGCCCCGGCG ACCGTAAGTC ATAGCCGGGG CTGGGGGAGC TTACTTTTGG CCTATGGCCA GGCAAAAGGC TACGTTAAGC CGGCTTGCGC
Clustal Consensus     **** *** * *********  **   ***** ******* ** **********  ********  ******** *  ********* **** ****  ********** **** *  ** ******* **


                      ....|....| ....|....| ....|....| ....|....| ....|....| ....|....| ....|....| ....|....| ....|....| ....|....| ....|....| ....|....|
                              250        260        270        280        290        300        310        320        330        340        350
360
Po12Bal02|FR831833    TATCCGACGC AAAGCAGGGG ACAGTGGTAG ACTACTTTAC GTTAAACCGG CTAACGCTGT CCGGTATCAA TGCAAGAAAA CGCGCTCGAC ACTGCGTTAA ACCGGCTAAT TTCGTGCGGT
Po36Ort01|FR831805    TATCCGACGC AAAGCAGGGG ACAGTGGTAG ACTACTTTAC GTTAAACCGG CTAACGCTGT CCGGTATCAA TGCAAGAAAA CGCGCTCGAC ACTGCGTTAA ACCGGCTAAT TTCGTGCGGT
Po06Ben02|FR831823    TATCCGACGC AAAGCAGGGG ACAGTGGTAG ACTACTTTAC GTTAAACCGG CTAACGCTGT CCGGTATCAA TGCAAGAAAA CGCGCTCGAC ACTGCGTTAA ACCGGCTAAT TTCGTGCGGT
Po06Ben20|FR831824    TATCCGACGC AAAGCAGGGG ACAGTGGTAG ACTACTTTAC GTTAAACCGG CTAACGCTGT CCGGTATCAA TGCAAGAAAA CGCGCTCGAC ACTGCGTTAA ACCGGCTAAT TTCGTGCGGT
Po16Ort05|FR831804    TATCCGACGC AAAGCAGGGG ACAGTGGTAG ACTACTTTAC GTTAAACCGG CTAACGCTGT CCGGTATCAA TGCAAGAAAA CGCGCTCGAC ACTGCGTTAA ACCGGCTAAT TTCGTGCGGT
Po19Art02|FR831814    TATCCGACGC AAAGCAGGGG ACAGTGGTAG ACTACCTTAC GTTAAACCGG CTAACGCTGT CCGGTATCAA TGCAAGAAAA CGCGCTCGAC ACTGCGTTAA ACCGGCTAAT TTCGTGCGGT
Po19Art04|FR831815    TATCCGACGC AAAGCAGGGG ACAGTGGTAG ACTACCTTAC GTTAAACCGG CTAACGCTGT CCGGTATCAA TGCAAGAAAA CGCGCTCGAC ACTGCGTTAA ACCGGCTAAT TTCGTGCGGT
Po36Ort05|FR831806    TATCCGACGC AAAGCAGGGG ACAGTGGTAG ACTACCTTAC GTTAAACCGG CTAACGCTGT CCGGTATCAA TGCAAGAAAA CGCGCTCGAC ACTGCGTTAA ACCGGCTAAT TTCGTGCGGT
Po11Art01|FR831811    TATCCGACGC AAAGCAGGGG ACAGTGGTAG ACTACCTTAC GTTAAACCGG CTAACGCTGT CCGGTATCAA TGCAAGAAAA CGCGCTCGAC ACTGCGTTAA ACCGGCTAAT TTCGTGCGGT
Po08Art01|FR831810    TATCCGACGC AAAGCAGGGG ACAGTGGTAG ACTACCTTAC GTTAAACCGG CTAACGCTGT CCGGTATCAA TGCAAGAAAA CGCGCTCGAC ACTGCGTTAA ACCGGCTAAT TTCGTGCGGT
Po06Mar06|FR831843    TATCTGACGC AAAGCAGGGG ACAGTGGTAG ACTACCTTAC GTTAAACCGG CTAACGCTGT CCGGTATCAA TGCAAGAAAA CGCGCTCGAC ACTGCGTTAA ACCGGCTAAT TTCGTGCGGT
Po07Ben04|FR831826    TATCCGACGC AAAGCAGGGG ACAGTGGTAG ACTACCTTAC GTTAAACCGG CTAACGCTGT CCGGCATCAA TGCAAGAAAA CGCGCTCGAC ACTGCGTTAA ACCGGCTAAT TTCGTGCGGT
Po19Art05|FR831816    TATCCGACGC AAAGCAGGGG ACAGTGGTAG ACTACTTTAC GTTAAACCGG CTAACGCTGT CTGGTATCAA TGCAAGAAAA CGCGCTCGAC ACTGCGTTAA ACCGGCCAAT TTCGTGCGGT
Po03Ort02|FR831801    TATCCGACGC AAAGCAGGGG ACAGTGGTAG ACTACTTTAC GTTAAACCGG CTAACGCTGT CCGGTATCAA TGCAAGAAAA CGCGCTCGAC ACTGCGCTAA ACCGGCCAAT TTCGTGCGGT
Po07Ben02|FR831825    TATCCGACGC AAAGCAGGGG ACAGTGGTAG ACTACCTTAC GTTAAACCGG CTAACGCTGT CCGGCATCAA TGCAAGAAAA CGCGCTCGAC ACTGCGTTAA ACCGGCCAAT TTCGTGCGGT
Po16Ort04|FR831803    TATCCGACGC AAAGCAGGGG ACAGTGGTAG ACTACTTTAC GTTAAACCGG CTAACGCTGT CCGGTATCAA TGCAAGAAAA CGCGCTCGAC ACTGCGTTAA ACCGGCCAAT TTCGTGCGGT
Po06Ben01|FR831822    TATCCGACGC AAAGCAGGGG ACAGTGGTAG ACTACTTTAC GTTAAACCGG CTAACGCTGT CCGGTATCAA TGCAAGAAAA CGCGCTCGAC ACTGCGTTAA ACCGGCCAAT TTCGTGCGGT
Po16Ort01|FR831802    TATCCGACGC AAAGCAGGGG ACAGTGGTAG ACTACTTTAC GTTAAACCGG CTAACGCTGT CCGGTATCAA TGCAAGAAAA CGCGCTCGAC ACTGCGTTAA ACCGGCCAAT TTCGTGCGGT
Po01Ben02|FR831818    TATCCGACGC AAAGCAGGGG ACAGTGGTAG ACTGCTTTAC GTTAAACCGG CTAACGCTGT CCGGTATCAA TGCAAGAAAA CGCGCTCGAC ACTGCGTTAA ACCGGCCAAT TTCGTGCGGT
Po01Ben01|FR831817    TATCCGACGC AAAGCAGGGG ACAGTGGTAG ACTACTTTAC GTTAAACCGG CTAGCGCTGT CCGGTATCAA TGCAAGAAAA CGCGCTCGAC ACTGCGCTAA ACCGGCCAAT TTCGTGCGGT
Po09Mar20|FR831849    TATCCGACGC AAAGCAGGGG ACAGTGGTAG ACTACTTTAC GTTAAACCGG CTAACGCTGT CCGGTATCAA TGCAAGAAAA CGCGCTCGAC ACTGCGTTAA ACCGGCTAAT TTCGTGCGGT
Po08Mar05|FR831845    TATCCGACGC AAAGCAGGGG ACAGTGGTAG ACTACTTTAC GTTAAACCGG CTAACGCTGT CCGGTATCAA TGCGAGAAAA CGCGCTCGAC ACTGCGTTAA ACCGGCTAAT TTCGTCCGGT
Po09Mar02|FR831848    TATCCGACGC AAAGCAGGGC ACAGTGGTAG ACTACTTTAC GTTAAACCGG CTAACGCTGT CCGGTATCAA TGCAAGAAAA CGCGCTCGAC ACTGCGTTAA ACCGGCTAAT TTCGTCCGGT
Po06Mar04|FR831841    TATCCGACGC AAAGCAGGGC ACAGTGGTAG ACTACTTTAC GTTAAACCGG CTAACGCTGT CCGGTATCAA TGCAGAAAAA CGCGCTCGAC ACTGCGTTAA ACCGGCTAAT TTCGTCCGGT
Po14Bal02|FR831835    TATCTGACGC AAAGCAGGGG ACAGTGGTAG ACTACTTTAC GTTAAACCGG CTAACGCTGT CCGGTATCAA TGCGAGAAAA CGCGCTCGAC ACTGCGTTAA ACCGGCTAAT TTCGTCCGGT
Po08Mar06|FR831846    TATCCGACGC AAAGCAGGGG ACAGTGGTAG ACTACTTTAC GTTAAACCGG CTAACGCTGT CCGGTATCAA TGCGAGAAAA CGCGCTCGAC ACTGCGTTAA ACCGGCTAAT TTCGTCCGGT
Po08Mar07|FR831847    TATCCGACGC AAAGCAGGGG ACAGTGGTAG ACTACTTTAC GTTAAACCGG CTAGCGCTGT CCGGTATCAA TGCAAGAAAA CGCGCTCGAC ACTGCGTTAA ACCGGCTAAT TTCGTCCGGT
Po08Mar04|FR831844    TATCCGACGC AAAGCAGGGG ACAGTGGTAG ACTACTTTAC GTTAAACCGG CTAACGCTGT CCGGTATCAA TGCAAGAAAA CGCGCTCGAC ACTGCGTTAA ACCGGCTAAT TTCGTCCGGT
Po11Bal01|FR831831    TATCCGACGC AAAGCAGGGC ACAGTGGTAG ACTACTTTAC GTTAAACCGG CTAACGCTGT CCGGTATCAA TGCAAGAAAA CGCGCTCGAC ACTGCGTTAA ACCGGCTAAT TTCGTCCGGT
```

```
Po11Bal02|FR831832    TATCCGACGC AAAGCAGGGC ACAGTGGTAG ACTACTTTAC GTTAAACCGG CTAACGCTGT CCGGTATCAA TGCAAGAAAA CGCGCTCGAC ACTGCGTTAA ACCGGCTAAT TTCGTCCGGT
Po06Mar02|FR831840    TATCCGACGC AAAGCAGGGC ACAGTGGTAG ACTACTTTAC GTTAAACCGG CTAACGCTGT CCGGTATCAA TGCAAGAAAA CGCGCTCGAC ACTGCGTTAA ACCGGCTAAT TTCGTCCGGT
Po06Mar05|FR831842    TATCCGACGC AAAGCAGGGC ACAGTGGTAG ACTACTTTAC GTTAAACCGG CTAACGCTGT CCGGTATCAA TGCAAGAAAA CGCGCTCGAC ACTGCGTTAA ACCGGCTAAT TTCGTCCGGT
Po36Ort07|FR831807    TATCCGACGC AAAGCAGGGG ACAGTGGTAG ACTACCTTAC GTTAAACCGG CTAACGCTGT CCGGTATCAA TGCAAGAAAA CGCGCTCGAC ACTGCGTTAA ACCGGCTAAT TTCGTCCGGT
Po19Art01|FR831813    TATCCGACGC AAAGCAGGGG ACAGTGGTAG ACTACTTTAC GTTAAACCGG CTAACGCTGT CCGGTATCAA TGCAAGAAAA CGCGCTCGAC ACTGCGTTAA ACCGGCCAAT TTCGTGCGGT
Po07Ben05|FR831827    TATCCGACGC AAAGCAGGGG ACAGTGGTGG ACTACTTTAC GTTAAACCGG CTAACGCTGT CCGGTATCAA TGCAAGAAAA CGCGCTCGAC ACTGCGTTAA ACCGGCTAAT TTCGTCCGGT
Clustal Consensus     **** ***** *********  ******** * *** * **** ********** *** ****** * ** ***** *** ****** ********** ****** *** ****** *** ***** ****


                      ....|....| ....|....| ....|....| ....|....| ....|....| ....|....| ....|....| ....|....| ....|....| ....|....| ....|....| ....|....|
                              370        380        390        400        410        420        430        440        450        460        470
480
Po12Bal02|FR831833    ATCAATGCAA GAAAACGCGC TCGACCGCCG CTACGGCACC GAGAAGCACA ACCGGCGCGG CATGAGCTAT GCGCATGCTT GGCTGCGTGC GCGCGCCCCT GGCGGTGGGT TCGGCTGTGT
Po36Ort01|FR831805    ATCAATGCAA GAAAACGCGC TCGACCGCCG CTACGGCACC GAGAAGCACA ACCGGCGCGG CATGAGCTAT GCGCATGCTT GGCTGCGTGC GCGCGCCCCT GGCGGTGGGT TCGGCTGTGT
Po06Ben02|FR831823    ATCAATGCAA GAAAACGCGC TCGACCGCCG CTACGGCACC GAGAAGCACA ACCGGCGCGG CATGAGCTAT GCGCATGCTT GGCTGCGTAC GCGCGCCCCT GGCGGTGGGT TCGGCTGTGT
Po06Ben20|FR831824    ATCAATGCAA GAAAACGCGC TCGACCGCCG CTACGGCACC GAGAAGCACA ACCGGCGCGG CATGAGCTAT GCGCATGCTT GGCTGCGTAC GCGCGCCCCT GGCGGTGGGT TCGGCTGTGT
Po16Ort05|FR831804    ATCAATGCAA GAAAACGCGC TCGACCGCCG CTACGGCACC GAGAAGCACA ACCGGCGCGG CATGAGCTAT GCGCATGCTT GGCTGCGTAC GCGCGCCCCT GGCGGTGGGT TCGGCTGTGT
Po19Art02|FR831814    ATCAATGCAA GAAAACGCGC TCGACCGCCG CTACGGCACC GAGAAGCACA ACCGGCGCGG CATGAGCTAT GCGCATGCTT GGCTGCGTGC GCGCGCCCCT GGCGGTGGGT TCGGCTGTGT
Po19Art04|FR831815    ATCAATGCAA GAAAACGCGC TCGACCGCCG CTACGGCACC GAGAAGCACA ACCGGCGCGG CATGAGCTAT GCGCATGCTT GGCTGCGTGC GCGCGCCCCT GGCGGTGGGT TCGGCTGTGT
Po36Ort05|FR831806    ATCAATGCAA GAAAACGCGC TCGACCGCCG CTACGGCACC GAGAAGCACA ACCGGCGCGG CATGAGCTAT GCGCATGCTT GGCTGCGTGC GCGCGCCCCT GGCGGTGGGT TCGGCTGTGT
Po11Art01|FR831811    ATCAATGCAA GAAAACGCGC TCGACCGCCG CTACGGCACC GAGAAGCACA ACCGGCGCGG CATGAGCTAT GCGCATGCTT GGCTGCGTGC GCGCGCCCCT GGCGGTGGGT TCGGCTGTGT
Po08Art01|FR831810    ATCAATGCAA GAAAACGCGC TCGACCGCCG CTACGGCACC GAGAAGCACA ACCGGCGCGG CATGAGCTAT GCGCATGCTT GGCTGCGTGC GCGCGCCCCT GGCGGTGGGT TCGGCTGTGT
Po06Mar06|FR831843    ATCAATGCAA GAAAACGCGC TCGACCGCCG CTACGGCACC GAGAAGCACA ACCGGCGCGG CATGAGCTAT GCGCATGCTT GGCTGCGTGC GCGCACCCCT GGCGGTGGGT TCGGCTGTGT
Po07Ben04|FR831826    ATCAATGCAA GAAAACGCGC TCGACCGCCG CTACGGCACC GAGAAGCACA ACCGGCGCGG CATGAGCTAT GCGCATGCTT GGCTGCGTGC GCGCGCCCCT GGCGGTGGGT TCGGCTGTGT
Po19Art05|FR831816    ATCAATGCAA GAAAACGCGC TCGACCGCCG CTACGGCACC GAGAAGCACA ACCGGCGCGG CATGAGCTAT GCGCATGCTT GGCTGCGTGC GCGCGCCCCT GGCGGTGGGT TCGGCTGTGT
Po03Ort02|FR831801    ATCAATGCAA GAAAACGCGC TCGACCGCCG CTACGGCACC GAGAAGCACA ACCGGCGCGG CATGAGCTAT GCGCATGCTT GGCTGCGTGC GCGCGCCCCT GGCGGTGGGT TCGGCTGTGT
Po07Ben02|FR831825    ATCAATGCAA GAAAACGCGC TCGACCGCCG CTACGGCACC GAGAAGCACA ACCGGCGCGG CATGAGCTAT GCGCATGCTT GGCTGCGTGC GCGCGCCCCT GGCGGTGGGT TCGGCTGTGT
Po16Ort04|FR831803    ATCAATGCAA GAAAACGCGC TCGACGGCCG CTACGGCACC GAGAAGCACA ACCGGCGCGG CATGAGCTAT GCGCATGCTT GGCTGCGTGC GCGCGCCCCT GGCGGTGGGT TCGGCTGTAT
Po06Ben01|FR831822    ATCAATGCAA GAAAACGCGC TCGACGGCCG CTACGGCACC GAGAAGCACA ACCGGCGCGG CATGAGCTAT GCGCATGCTT GGCTGCGTGC GCGCGCCCCT GGCGGTGGGT TCGGCTGTAT
Po16Ort01|FR831802    ATCAATGCAA GAAAACGCGC TCGACGGCCG CTACGGCACC GAGAAGCACA ACCGGCGCGG CATGAGCTAT GCGCATGCTT GGCTGCGTAC GCGCGCCCCT GGCGGTGGGT TCGGCTGTGT
Po01Ben02|FR831818    ATCAATGCAA GAAAACGCGC TCGACCGCCG CTACGGCACC GAGAAGCACA ACCGGCGCGG CATGAGCTAT GCGCATGCTT GGCTGCGTAC GCGCGCCCCT GGCGGTGGGT TCGGCTGTGT
Po01Ben01|FR831817    ATCAATGCAA GAAAACGCGC TCGACCGCCG CTACGGCACC GAGAAGCACA ACCGGCGCGG CATGAGCTAT GCGCATGCTT GGCTGCGTGC GCGCGCCCCT GGCGGTGGGT TCGGCTGTAT
Po09Mar20|FR831849    ATCAATGCAA GAAAACGCGC TCGACCGCCG CTACGGCACC GAGAAGCACA ACCGGCGCGA CATGAGCTAT GCGCATGCTT GGCTGCGTGC GCGCGCCTCT GGCGGTGGGT TCGGCTGTGT
Po08Mar05|FR831845    ATCAATGCAA GAAAACGCGC TCGACCGCCG CTACGGCACC GAGAAGCACA ACCGGCGCGG CATGAGCTAT GCGCATGCTT GGCCGCGTGC GCGCGCCCCT GGCGGTGGGT TCGGCTGTGT
Po09Mar02|FR831848    ATCAATGCAA GGAAAGGCGC TCGACCGCCG CTACGGCACC GAGAAACACA ACCGGCGCGA CATGAGCTAT GCGCATGCTT GGCTGCGTGC GCGCGCCTCT GGCGGTGGGT TCGGCTGTGT
Po06Mar04|FR831841    ATCAATGCAA GAAAACGCGC TCGACCGCCG CTACGGCACC GAGAAGCACA ACCGGCGCGG CATGAGCTAT GCGCATGCTT GGCTGCGTGC GCGCGCCCCT GGCGGTGGGT TCGGCTGTGT
Po14Bal02|FR831835    ATCAATGCAA GAAAACGCGC TCGACCGCCG CTACGGCATC GAGAAACACA ACCGGCGCGG CATGAGCTAT GCGCATGCCT GGCTGCGTGC GCGCGCCCCT GGCGGTGGGT TCGGCTGTGC
Po08Mar06|FR831846    ATCAATGCAA GAAAACGCGC TCGACCGCCG CTACGGCACC GAGAAACACA ACCGGCGCGG CATGAGCTAT GCGCATGCCT GGCTGCGTGC GCGCGCCCCT GGCGGTGGGT TCGGCTGTGC
Po08Mar07|FR831847    ATCAATGCAA GAAAACGCGC TCGACCGCCG CTACGGCACC GAGAAACACA ACCGGCGCGG CGTGAGCTAT GCGCATGCCT GGCTGCGTGC GCGCGCCCCT GGCGGTGGGT TCGGCTGTGC
Po08Mar04|FR831844    ATCAATGCAA GAAAACGCGC TCGACCGCCG CTACGGCACC GAGAAACACA ACCGGCGCGG CATGAGCTAT GCGCATGCCT GGCTGCGTGC GCGCGCCCCT GGCGGTGGGT TCGGCTGTGT
Po11Bal01|FR831831    ATCAATGCAA GAAAAGGCGC TGGACCGCCG CTACGGCACC GAGAAACACA ACCGGCGCGG CATGAGCAAT GCGCATGCTT GGCTGCGTGC GCGCGCCCCT GGCGGTGGGT TCGGCTGTGT
Po11Bal02|FR831832    ATCAATGCAA GAAAAGGCGC TGGACCGCCG CTACGGCACC GAGAAACACA ACCGGCGCGG CATGAGCAAT GCGCATGCTT GGCTGCGTGC GCGCGCCCCT GGCGGTGGGT TCGGCTGTGT
Po06Mar02|FR831840    ATCAATGCAA GAAAAGGCGC TGGACCGCCG CTACGGCACC GAGAAACACA ACCGGCGCGG CATGAGCAAT GCGCATGCTT GGCTGCGTGC GCGCGCCCCT GGCGGTGGGT TCGGCTGTGT
Po06Mar05|FR831842    ATCAATGCAA GAAAAGGCGC TGGACCGCCG CTACGGCACC GAGAAACACA ACCGGCGCGG CATGAGCAAT GCGCATGCTT GGCTGCGTGC GCGCGCCCCT GGCGGTGGGT TCGGCTGTGT
Po36Ort07|FR831807    ATCAATGCAA GAAAAGGCGC TGGACCGCCG CTACGGCACC GAGAAACACA ACCGGCGCGG CATGAGCAAT GCGCATGCTT GGCTGCGTGC GCGCGCCCCT GGCGGTGGGT TCGGCTGTGT
Po19Art01|FR831813    ATCAATGCAA GAAAACGCGC TCGACCGCCG CTACGGCACC GAGAAACACA ACCGGCGCGG CATGAGCAAT GCGCATGCTT GGCTGCGTGC GCGCGCCCCT GGCGGTGGGT TCGGCTGTGT
Po07Ben05|FR831827    ATCAATGCAA GAAAACGCGC TCGACCGCCG CTACGGCACC GAGAAGCACA ACCGGCGCGG CATGAGCTAT GCGCATGCTT GGCTGCGTGC GCGCGCCCCT GGCGGTGGGT TCGGCTGTGT
Clustal Consensus     ********** * *** **** * *** **** ******** * ***** **** *********  * ***** ** ******** * *** **** * **** ** ** ********** ********


                      ....|....| ....|....| ....|....| ....|....| ....|....| ....|....| ....|....| ....|....| ....|....| ....|....| ....|....| ....|....|
                              490        500        510        520        530        540        550        560        570        580        590
600
Po12Bal02|FR831833    TGCATAGAGC GCAGGGGAAA TGCGCAAGAG TCTTTTTCAC GCGTGCGAAC AGATATATGC GCAGCGCAGG CCGGTTGTCA TGCGCTGACG GCGGACGCAT TCGCGCGGCC ACCGGCAACG
Po36Ort01|FR831805    TGCATAGAGC GCAGGGGAAA TGCGCAAGAG TCTTTTTCAC GCGTGCGAAC AAATATGTGC GCAGCGCAGG CCGGTTGTCA TGCGCTGACG GCGGACGCAT TCGCGCGGCC ACCGGCAACG
Po06Ben02|FR831823    TGCATAGAGC GCAGGGGAAA TGCGCAAGAG TCTTTTTCAC GCGTGCGAAC AAATATATGC GCAGCGCAGG CCGGTTGTCA TGCGCTGACG GCGGACGCAT TCGCGCGGCC ACCGGCAACG
Po06Ben20|FR831824    TGCATAGAGC GCAGGGGAAA TGCGCAAGAG TCTTTTTCAC GCGTGCGAAC AAATATATGC GCAGCGCAGG CCGGTTGTCA TGCGCTGACG GCGGACGCAT TCGCGCGGCC ACCGGCAACG
Po16Ort05|FR831804    TGCATAGAGC GCAGGGGAAA TGCGCAAGAG TCTTTTTCAC GCGTGCGAAC AAATATATGC GCAGCGCAGG CCGGTTGTCA TGCGCTGACG GCGGACGCAT TCGCGCGGCC ACCGGCAACG
Po19Art02|FR831814    TGCATAGAGC GCAGGGGAAA TGCGCAAGAG TATTTTTCAC ACGTGCGAAC AAATATATGC GCAGCGCAGG CCGCTCGTCA TGCGCTGATG GCGGTCGCAT TCGCACGGCC ACCGGCAACG
Po19Art04|FR831815    TGCATAGAGC GCAGGGGAAA TGCGCAAGAG TATTTTTCAC ACGTGCGAAC AAATATATGC GCAGCGCAGG CCGCTCGTCA TGCGCTGATG GCGGTCGCAT TCGCACGGCC ACCGGCAACG
Po36Ort05|FR831806    TGCATAGAGC GCAGGGGAAA TGCGCAAGAG TCTTTTTCAC GCGTGCGAAC AAATATGTGC GCAGCGCAGG CCGGTTGTCA TGCGCTGACG GCGGACGCAT TCGCGCGGCC ACCGGCAACG
```

```
Po11Art01|FR831811    TGCATAGAGC GCAGGGGAAA TGCGCAAGAG TCTTTTTCAC GCGTGCCAAC AAATATATGC GCAGCGCAGG ACGGTTGTCA TGCGCTGACG GCGGACGCAT TCGCGCGGCC ACCGGCAACA
Po08Art01|FR831810    TGCATAGAGC GCAGGGGAAA TGCGCAAGAG TCTTTTTCAC GCGTGCGAAC AAATATATGC GCAGCGCAGG ACGGTTGTCA TGCGCTGACG GCGGACGCAT TCGCGCGGCC ACCGGCAACA
Po06Mar06|FR831843    TGCATAGAGC GCAGGGGAAA TGCGCAAGAG TCTTTTTCAC GCGTGCGAAC AAATATATGC GCAGCGCAGG CCGGTTGTCA TGCGCTGACG GCGGACGCAT TCGCGCGGCC ACCGGCAACG
Po07Ben04|FR831826    TGCATAGAGC GCAGGGGAAA TGCGCAAGAG TCTTTTTCAC GCGTGCGAAC AAATATATGC GCAGCGCTGG CCGGTTGTCA TGCGCTGACG GCGGACGCAT TCGCGCGGCC ACCGGCAACG
Po19Art05|FR831816    TGCGTAGAGC GCAGGGGAAA TGCGCAAGAG TCTTTTTCAC GCGTGCCAAC AAATATATGC GCAGCGCAGG CCGGTTGTCA TGCGCTGACG GCGGACGCAT TCGCGCGGCC ACCGGCAACG
Po03Ort02|FR831801    TGCGTAGAGC GCAGGGGAAA TGCGCAAGAG TCTTTTTCAC GCGTGCCAAC AAATATATGC GCAGCGCAGG CCGGTTGTCA TGCGCTGACG GCGGACGCAT TCGCGCGGCC ACCGGCAACG
Po07Ben02|FR831825    TGCGTAGAGC GCAGGGGAAA TGCGCAAGAG TCCTTTTCAC GCGTGCCAAC AAATATATGC GCAGCGCAGG CCGGTTGTCA TGCGCTGACG GCGGACGCAT TCGCGCGGCC ACCGGCAACG
Po16Ort04|FR831803    TGCATAGAGC GCAGGGGAAA TGCGCAAGAG TCTTTTTCAC GCGTGCGAAC AAATATATGC GCAGCGCAGG CCGGTTGTCA TGCGCTGACG GCGGACGCAT TCGCGCGGCC ACCGGCAACG
Po06Ben01|FR831822    TGCATAGAGC GCAGGGGAAA TGCGCAAGAG TCTTTTTCAC GCGTGCGAAC AAATATATGC GCAGCGCAGG CCGGTTGTCA TGCGCTGACG GCGGACGCAT TCGCGCGGCC ACCGGCAACG
Po16Ort01|FR831802    TGCATAGAGC GCAGGGGAAA TGCGCAAGAG TCTTTTTCAC GCGTGCGAAC AAATATATGC GCAGCGCAGG CCGGTTGTCA TGCGCTGACG GCGGACGCAT TCGCGCGGCC ACCGGCAACG
Po01Ben02|FR831818    TGCATAGAGC GCAGGGGAAA TGCGCAAGAG TCTTTTTCAC GCGTGCGAAC AAATATATGC GCAGCGCAGG CCGGTTGTCA TGCGCTGACG GCGGACGCAT TCGCGCGGCC ACCGGCAACG
Po01Ben01|FR831817    TGCATAGAGC GCAGGG-AAA TGCGCAAGAG TCTTTTTCAC GCGTGCGAAC AAATATATGC GCAGCGTAGG CCGGTTGTCA TGCGCTGACG GCGGACGCAT TCGCGCGGCC ACCGGCAACG
Po09Mar20|FR831849    TGCATAGAGC GCAGGGGAAA TGCGCAAGAG TCTTTTTCAC GCGTGCGAAC AAATATATGC GCAGCGCAGG CCGGTTGTCA TGCGCTGACG GCGGACGCAT TCGCGCGGCC ACCGGCAACG
Po08Mar05|FR831845    TGCATAGAGC GCAGGGGAAA TGCGCAAGAG TCTTTTTCAC GCGTGCGAAC AAATATATGC GCAGCGCAGG CCGGTTGTCA TGCGCTGACG GCGGACGCAT TCGCGCGGCC ACCGGCAACG
Po09Mar02|FR831848    TGCATAGAGC GCAGGGGAAA TGCGCAAGAG TCTTTTTCAC GCGTGCGAAC AAATATATGC GCAGCGCAGG CCGGTTGTCA TGCGCTGACG GCGGACGCAT TCGCGCGGCC ACCGGCAACG
Po06Mar04|FR831841    TGCATAGAGC GCAGGGGAAA TGCGCAAGAG TCTTTTTCAC GCGTGCGAAC AAATATATGC GCAGCGCAGG ACGGTTGTCA TGCGCTGACG GCGGACGCAT TCGCGCGGCC ACCGGCAACA
Po14Bal02|FR831835    TGCATAGAGC GCAGGGGAAA TGCGCAAGAG TCTTTTTCAC GCGTGCCAAC AAATATATGC GCAGCGCAGG CCGGTTGTCA TGCGCTGACG GCGGACGCAT TCGCGCGGCC ACCGGCAACG
Po08Mar06|FR831846    TGCATAGAGC GCAGGGGAAA TGCGCAGGAG TCTTTTTCAC GCGTGCCAAC AAATATATGC GCAGCGCAGG CCGGTTGTCA TGCGCTGACG GCGGACGCAT TCGCGCGGCC ACCGGCAACG
Po08Mar07|FR831847    TGCATAGAGC GCAGGGGAAA TGCGCAAGAG TCTTTTTCAC GCGTGCCAAC AAATATATGC GCAGCGCAGG CCGGTTGTCA TGCGCTGACG GCGGACGCAT TCGCGCGGCC ACCGGCAACG
Po08Mar04|FR831844    TGCATAGAGC GCAGGGGAAA TGCGCAAGAG TCTTTTTCAC GCGTGCCAAC AAATATATGC GCAGCGCAAG CCGGTTGTCA TGCGCTGACG GCGGACGCAT TCGCGCGGCC ACCGGCAACG
Po11Bal01|FR831831    TGCATAGAGC GCAGGGGAAA TGCGCAAGAG TCTTTTTCAC GCGTGCCAAC AAATATATGC GCAGCGCA-G CCGGTTGTTA TGCGCTGACG GCGGACGCAT TCGCGCGGCC ACCGGCAACG
Po11Bal02|FR831832    TGCATAGAGC GCAGGGGAAA TGCGCAAGAG TCTTTTTCAC ACGTGCCAAC AAATATATGC GCAGTGCACG CCGGTTGTTA TGCGCTGACG GCGGACGCAT TCGCGCGGCC ACCGGCAACG
Po06Mar02|FR831840    TGCATAGAGC GCAGGGGAAA TGCGCAAGAG TCTTTTTCAC GCGTGCCAAC AAATATATGC GCAGCGCAGG CCGGTTGTTA TGCGCTGACG GCGAACGCAT TCGCGCGGCC ACCGGCAACG
Po06Mar05|FR831842    TGCATAGAGC GCAGGGGAAA TGCGCAAGAG TCTTTTTCAC GCGTGCCAAC AAATATATGC GCAGCGCAGG CCGGTTGTTA TGCGCTGACG GCGAACGCAT TCGCGCGGCC ACCGGCAACG
Po36Ort07|FR831807    TGCATAGAGC GCAGGGGAAA TGCGCAAGAG TCTTTTTCAC GCGTGCCAAC AAATATATGC GCAGCGCAGG CCGGTTGTTA TGCGCTGACG GCGGACGCAT TCGCGCGGCC ACCGGCAACG
Po19Art01|FR831813    TGCATAGAGC GCAGGGGAAA TGCGCAAGAG TCTTTTTCAC GCGTGCCAAC AAATATATGC GCAGCGCAGG CCGGTTGTTA TGCGCTGACG GCGAACGCAT TCGCGCGGCC ACCGGCAACG
Po07Ben05|FR831827    TGCATAGAGC GCAGGGGAAA TGCGCAAGAG TCTTTTTCAC GCGTGCCAAC AAATATATGC GCAGCGCAGG CCGGTTGTCA TGCGCTGACG GCGGACGCAT TCGCGCGGCC ACCGGCAACG
Clustal Consensus     *** ****** ****** *** ****** *** *  *******  ***** *** * **** *** **** *   *  ** * ** * ******** * ***  ***** **** ***** *********


                      ....|....

Po12Bal02|FR831833    AATTCGCCC
Po36Ort01|FR831805    AATTCGCCC
Po06Ben02|FR831823    AATTCGCCC
Po06Ben20|FR831824    AATTCGCCC
Po16Ort05|FR831804    AATTCGCCC
Po19Art02|FR831814    GATCCGCCC
Po19Art04|FR831815    GATCCGCCC
Po36Ort05|FR831806    AATTCGCCC
Po11Art01|FR831811    AATTCGCCC
Po08Art01|FR831810    AATTCGCCC
Po06Mar06|FR831843    AATTCGCCC
Po07Ben04|FR831826    AATTCGCCC
Po19Art05|FR831816    AATTCGCCC
Po03Ort02|FR831801    AATTCGCCC
Po07Ben02|FR831825    AATTCGCCC
Po16Ort04|FR831803    AATTCGCCC
Po06Ben01|FR831822    AATTCGCCC
Po16Ort01|FR831802    AATTCGCCC
Po01Ben02|FR831818    AATTCGCCC
Po01Ben01|FR831817    AATTCGCCC
Po09Mar20|FR831849    AATTCGCCC
Po08Mar05|FR831845    AATTCGCCC
Po09Mar02|FR831848    AATTCGCCC
Po06Mar04|FR831841    AATTCGCCC
Po14Bal02|FR831835    AACTCGCCC
Po08Mar06|FR831846    AACTCGCCC
Po08Mar07|FR831847    AACTCGCCC
Po08Mar04|FR831844    AATTCGCCC
Po11Bal01|FR831831    GATTCGCCC
```

```
Po11Bal02|FR831832   GATTCGCCC
Po06Mar02|FR831840   GATTCGCCC
Po06Mar05|FR831842   GATTCGCCC
Po36Ort07|FR831807   GATTCGCCC
Po19Art01|FR831813   GATTCGCCC
Po07Ben05|FR831827   GATTCGCCC
Clustal Consensus     *  *****
```

B type

```
                     ....|....| ....|....| ....|....| ....|....| ....|....| ....|....| ....|....| ....|....| ....|....| ....|....| ....|....| ....|....|
                             10         20         30         40         50         60         70         80         90        100        110
120
Po02Art01|FR831809   GTCTACGGCC ACATCACGTT GAAAACACCG GTTCCCGTCC GATCACCGAA GTTAAGCAAC GTCGGGCCCG GTCAGTACTT GGATGGGTGA CCGCCTGGGA ACACCGGGTG CTGTAGACGC
Po17Art01|FR831812   GTCTACGGCC ACATCACGTT GAAAACACCG GTTCCCGTCC GATCACCGAA GTTAAGCAAC GTCGGGCCCG GTCAGTACTT GGATGGGTGA CCGCCTGGGA ACACCGGGTG CTGTAGACGC
Po01Art01|FR831808   GTCTACGGCC ACATCACGTT GAAAACACCG GTTCTCGTCC GATCACCGAA GTTAAGCAAC GTCGGGCCCG GTCAGTACTT GGATGGGTGA CCGCCTGGGA ACACCGGGTG CTGTAGACGC
Clustal Consensus    ********** ********** ********** **** ***** ********** ********** ********** ********** ********** ********** ********** **********

                     ....|....| ....|....| ....|....| ....|....| ....|....| ....|....| ....|....| ....|....| ....|..
                            130        140        150        160        170        180        190        200
Po02Art01|FR831809   CTTTTTAAAA ACGACAAAAA CCAAAAAAAA ATCAATGAGC GAGTCAATTC AGGTAAGTAT AACTACGGGT TCTGTTCACC TTCTCTC
Po17Art01|FR831812   CTTTTTAAAA ACGACAAAAA CCAAAAAAAA ATCAATGAGC GAGTCAATTC AGGTAAGTAT AACTACGGGT TCTGTTCACC TTCTCTC
Po01Art01|FR831808   CTTTTTAAAA ACAAAACAAA --AAAAAAAA ATCAATAAGC GGGTCAATAC AG--TAGTAT AACTACGGGT TTTGTCCGCC TTCTCTC
Clustal Consensus    ********** ** * * ***   ******** ****** *** * ****** * **   ***** ********** * *** * ** *******
```

C type and pseudogenes

```
                     ....|....| ....|....| ....|....| ....|....| ....|....| ....|....| ....|....| ....|....| ....|....| ....|....| ....|....| ....|....|
                             10         20         30         40         50         60         70         80         90        100        110
120
Po06Bal01b|FR831828  GTCTACGGCC ATACCGCGTT GAAAACACCA GTTCTCGTCC GATCACTGAA GTTAAGCAAC GTCGGGCCCG GTCAGTACTT GGATGGGTGA CCGCCTGGGA ACACCGGGTG CTGTAGACGC
Po06Bal02b|FR831829  GTCTACGGCC ATACCGCGTT GAAAACACCA GTTCTCGTCC GATCACTGAA GTTAAGCAAC GTCGGGCCCG GTCAGTACTT GGATGGGTGA CCGCCTGGGA ACACCGGGTG CTGTAGACGC
Po06Bal04|FR831830   GTCTACGACC ATATCACGTT GAAAACACCG GTTCTCGTCC GATCACCGAA GTTAAGCAAC GTCGGGCCCG GTTAGTACTT GGATGGGTGA CCGCCTGGGA ACACCGGGTG CTGTAGACGC
Po06Bal01a|FR831828  GTCTACGGCC ACATCACGTT GAAAACACCG GTTCCCGTCC GATCACCGAA GTTAAGCAAC GTCGGGCCCG GTCAGTACTT G-------GA CCGTCTGGGA ACACTGGGTG CTGTTTGTTC
Po06Bal02a|FR831829  GTCTACGGCC ACATCACGTT GAAAACACCG GTTCCCGTCC GATCACCGAA GTTAAGCAAC GTCGGGCCCG GTCAGTACTT G-------GA CCGTCTGGGA ACACTGGGTG CTGTTTGTTC
Clustal Consensus    ******* ** * * * **** *********  **** ***** ****** *** ********** ********** ** ******* *       ** *** ****** **** ***** ****     *

                     ....|....| ....|....| ....|....| ....|....| ....|....| ....|....| ....|....| ....|....| ....|....| ....|....| ....|....| ....|....|
                            130        140        150        160        170        180        190        200        210        220        230
240
Po06Bal01b|FR831828  CTTTTTGCCC TGATTCTTCG TGAAAAAATC AATTTTCCGC CAATCACACC ACAGCGCGCA CTCTTTTGCA CCACTCCAGC TCTGCGCTGG GCCCGTCTCC CCAGGGAATA AGACACAAAA
Po06Bal02b|FR831829  CTTTTTGCCC TGATTCTTCG TGAAAAAATC AATTTTCCGC CAATCACACC ACAGCGCGCA CTCTTTTGCA CCACTCCAGC TCTGCGCTGG GCCCGTCTCC CCAGGGAATA AGACACAAAA
Po06Bal04|FR831830   CTTTTTGCCC TGATTCTTCG TGAAAAAATC AATTTTCCGC CCATCAGGCC ACAGCGCGCA CTCTTTTGCA CCACTCCACC GCTCCGCTCG GCTCGTTTCC CGAGAGAAAA AGACACAAAA
Po06Bal01a|FR831828  ACCTTCTCTC ---------- ---------- ---------- ---------- ---------- ---------- ---------- ---------- ---------- ---------- ----------
Po06Bal02a|FR831829  ACCTTCTCTC ---------- ---------- ---------- ---------- ---------- ---------- ---------- ---------- ---------- ---------- ----------
Clustal Consensus       **  * *

                     ....|....| ....|....| ....|....| ....|....| ....
                            250        260        270        280
Po06Bal01b|FR831828  AGAATTTGAG TAAGCACAAG TATGGGTTCT GTCCACCTTC TCTC
```

```
Po06Bal02b|FR831829   AGAATTTGAG TAAGCACAAG TATGGGTTCT GTCCACCTTC TCTC
Po06Bal04|FR831830    AGAATTTGAG TAGGTATAAC TACGGGTTCT GTCCACCTTC TCTC
Po06Bal01a|FR831828   ---------- ---------- ---------- ---------- ----
Po06Bal02a|FR831829   ---------- ---------- ---------- ---------- ----
Clustal Consensus
```

D type

```
                      ....|....| ....|....| ....|....| ....|....| ....|....| ....|....| ....|....| ....|....| ....|....| ....|....| ....|....| ....|....|
                              10         20         30         40         50         60         70         80         90        100        110
120
El03Afu02b|FR831850   GGCAACGACC ATATCACGTT GAAAACACCG GTTCCCGTCC GATCACCGAA GTTAAGCAGC GTCGGGCCCG GTTAGTACTT GGATGGGTGA CCGCCTGGGA ACACCGGGTG CCGTTGTCAC
El03Afu19b|FR831854   GGCAACGACC ATACCACGCT GAAAACACCA GTTCCCGTCC GATCACTGAA GTTAAGCAGC GTCGGGCCCG GTTAGTACTT GGATGGGTGA CCGCCTGGGA ACACCGGGTG CCGTTGTCAC
Clustal Consensus     ********** *** **** * *********  ********** ****** *** ********** ********** ********** ********** ********** ********** **********

                      ....|....| ....|....| ....|....| ....|....| ....|....| ....|....| ....|....| ....|...
                             130        140        150        160        170        180        190
El03Afu02b|FR831850   ATTTTGCGTG CCACATTTTG CGTAAATGTG CCACGTATTG TTTGCCAAGT TGGTCGGCCA CCGGCAATGC ACGCTCTC
El03Afu19b|FR831854   ATTTTGCGTG CCACATTTTG CGTAAATGTG CCACGTATTG TTTGCCAAGT TGGTCGGCCA CCGGCAATGC ACGCTCTC
Clustal Consensus     ********** ********** ********** ********** ********** ********** ********** ********
```

E type

```
                      ....|....| ....|....| ....|....| ....|....| ....|....| ....|....| ....|....| ....|....| ....|....| ....|....| ....|....| ....|....|
                              10         20         30         40         50         60         70         80         90        100        110
120
El04Tie01|FR831870    GGCAACGACC ATATCACGTT GAAAACACCG GTTCCCGTCC GATCACCGAA GTTAAGCAGC GTCGGGCCCG GTTAGTACTT GGATGGGTGA CCGCCTGGGA ACACCGGGTG CCGTTGTCAC
El04Tie06a|FR831872   GGCAACGACC ATATCACGTT GAAAACACCG GTTCCCGTCC GATCACCGAA GTTAAGCAGC GTCGGGCCCG GTTAGTACTT GGATGGGCGA CCGCCTGGGA ACACCGGGTG CCGTTGTCAC
El04Tie03|FR831871    GGCAACGACC ATATCACGTT GAAAACACCG GTTCCCGTCC GATCACCGAA GTTAAGCAGC GTCGGGCCCG GTTAGTACTT GGATGGGTGA CCGCCTGGGA ACACCGGGTG CCGTTGTCAC
El04Tie09b|FR831873   GGCAACGACC ATATCACGTT GAAAACACCA GTTCCCCTCC GATCACCGAA GTTAAGCAGC GTCGGGCCCG GTTAGTACTT GGATGGGTGA CCGCCTGGGA ACACCGGGTG CCGTTGTCAC
El04Tie09a|FR831873   GGCAACGACC ATATCACGTT GAAAACACCG GTTCCCGTCC GATCACCGAA GTTAAGCAGC GTCGGGCCCG GTTAGTACTT GGATGGGTGA CCGCCTGGGA ACACCGGGTG CCGTTGTCAC
El05Afu03|FR831860    GGCAACGACC ATACCACGCT GAAAACACCA GTTCCCGTCC GATCACCGAA GTTAAGCAGC GTCGGGCCCG GTTAGTACTT GGATGGGTGA CCGCCTGGGA ACACCGGGTG CCGTTGTCAC
El04Tie06b|FR831872   GGCAACGACC ATACCACGCT GAAAACACCA GTTCCCGTCC GATCACCGAA GTTAAGCAGC GTCGGGCCCG GTTAGTACTT GGATGGGTGA CCGCCTGGGA ACACCGGGTG CCGTTGTCAC
El07Tie01|FR831875    GGCAACGACC ATACCACGTT GAAAACACCA GTTCCCGTCC GATCACCGAA GTTAAGCAGC GTCGGGCCCG GTTAGTACTT GGATGGGTGA CCGCCTGGGA ACACCGGGTG CCGTTGTCAC
El03Afu15|FR831852    GGCAACGACC ATATCACGTT GAAAACACCG GTTCCCGTCC GATCACCGAA GTTAAGCAGC GTCGGGCCCG GTTAGTACTT GGATGGGTGA CCGCCTGGGA ACACCGGGTG CCGTTGTCAC
El03Afu31|FR831856    GGCAACGACC ATATCACGTT GAAAACACCG GTTCCCGTCC GATCACCGAA GTTAAGCAGC GTCGGGCCCG GTTAGTACTT GGATGGGTGA CCGCCTGGGA ACACCGGGTG CCGTTGTCAC
El03Afu02a|FR831850   GGCAACGACC ATATCACGTT GAAAACACCG GTTCCCGTCC GATCACCGAA GTTAAGCAGC GTCGGGCCCG GTTAGTACTT GGATGGGTGA CCGCCTGGGA ACACCGGGTG CCGTTGTCAC
El03Afu07a|FR831851   GGCAACGACC ATATCACGTT GAAAACACCG GTTCCCGTCC GATCACCGAA GTTAAGCAGC GTCGGGCCCG GTTAGTACTT GGATGGGTGA CCGCCTGGGA ACACCGGGTG CCGTTGTCAC
El03Afu07b|FR831851   GGCAACGACC ATACCACGCT GAAAACACCA GTTCCCGTCC GATCACCGAA GTTAAGCAGC GTCGGGCCCG GTTAGTACTT GGATGGGTGA CCGCCTGGGA ACACCGGGTG CCGTTGTCAC
El03Afu16b|FR831853   GGCAACGACC ATACCACGCT GAAAACACCA GTTCCCGTCC GATCACCGAA GTTAAGCAGC GTCGGGCCCG GTTAGTACTT GGATGGGTGA CCGCCTGGGA ACACCGGGTG CCGCTGTCAC
El09Tie06|FR831878    GGCAACGACC ATATCACGTT GAAAACACCG GTTCCCGTCC GATCACCGAA GTTAAGCAGC GTCGGGCCCG GTTAGTACTT GGATGGGTGA CCGCCTGGGA ACACCGGGTG CCGTTGTCAC
El02Tie01|FR831869    GGCAACGACC ATACCACGTT GAAAACACCA GTTCCCGTCC GATCACCGAA GTTAAGCAGC GTCGGGCCCG GTTAGTACTT GGATGGGTGA CCGCCTGGGA ACACCGGGTG CCGTTGTCAC
El07Tie02|FR831876    GGCAACGACC ATACCACGTT GAAAACACCA GTTCCCGTCC GATCACCGAA GTTAAGCAGC GTCGGGCCCG GTTAGTACTT GGATGGGTGA CCGCCTGGGA ACACCGGGTG CCGTTGTCAC
El05Afu04a|FR831861   GGCAACGACC ATACCACGTT GAAAACACCA GTTCCCGTCC GATCACCGAA GTTAAGCAGC GTCGGGCCCG GTTAGTACTT GGATGGGTGA CCGCCTGGGA ACACCGGGTG CCGTTGTCAC
El05Afu04b|FR831861   GGCAACGACC ATACCACGCT GAAAACACCA GTTCCCGTCC GATCACTGAA GTTAAGCAGC GTCGGGCCCG GTTAGTACTT GGATGGGTGA CCGCCTGGGA ACACCGGGTG CCGTTGTCAC
El04Afu05|FR831858    GGCAACGACC ATACCACGTT GAAAACACCA GTTCCCGTCC GATCACCGAA GTTAAGCAGC GTCGGGCCCG GTTAGTACTT GGATGGGTGA CCGCCTGGGA ACACCGGGTG CCGTTGTCAC
El03Afu16a|FR831853   GGCAACGACC ATATCACGTT GAAAACACCG GTTCCCGTCC GATCACCGAA GTTAAGCAGC GTCGGGCCCG GTTAGTACTT GGATGGGTGA CCGCCTGGGA ACACCGGGTG CCGTTGTCAC
El03Afu19a|FR831854   GGCAACGACC ATATCACGTT GAAAACACCG GTTCCCGTCC GATCACCGAA GTTAAGCAGC GTCGGGCCCG GTTAGTACTT GGATGGGTGA CCGCCTGGGA ACACCGGGTG CCGTTGTCAC
El03Afu02c|FR831850   GGCAACGACC ATACCACGCT GAAAACACCA GTTCCCGTCC GATCACTGAA GTTAAGCAGC GTCGGGCCCG GTTAGTACTT GGATGGGTGA CCGCCTGGGA ACACCGGGTG CCGTTGTCAC
```

```
El09Tie01|FR831877    GGCAACGACC ATATCACGTT GAAAACACCG GTTCCCGTCC GATCACCGAA GTTAAGCAGC GTCGGGCCCG GTTAGTACTT GGATGGGTGA CCGCCTGGGA ACACCGGGTG CCGTTGTCAC
El01Tie10|FR831866    GGCAACGACC ATACCACGTT GAAAACACCA GTTCCCGTCC GATCACCGAA GTTAAGCAGC GTCGGGCCCG GTTAGTACTT GGATGGGTGA CCGCCTGGGA ACACCGGGTG CCGTTGTCAC
El01Tie02a|FR831863   GGCAACGACC ATACCACGTT GAAAACACCA GTTCCCGTCC GATCACCGAA GTTAAGCAGC GTCGGGCCCG GTTAGTACTT GGATGGGTGA CCGCCTGGGA ACACCGGGTG CCGTTGTCAC
Po02Ben02|FR831820    GGCAACGACC ATACCACGTT GAAAACACCA GTTCCCGTCC GATCACCGAA GTTAAGCAGC GTCGGGCCCG GTTAGTACTT GGATGGGTGA CCGCCTGGGA ACACCGGGTG CCGTTGTCAC
Po02Ben03|FR831821    GGCAACGACC ATACCACGTT GAAAACACCA GTTCCCGTCC GATCACCGAA GTTAAGCAGC GTCGGGCCCG GTTAGTACTT GGATGGGTGA CCGCCTGGGA ACACCGGGTG CCGTTGTCAC
El01Tie02b|FR831863   GGCAACGACC ATACCACGCT GAAAACACCA GTTCCCGTCC GATCACTGAA GTTAAGCAGC GTCGGGCCCG GTTAGTACTT GGATGGGTGA CCGCCTGGGA ACACCGGGTG CCGTTGTCAC
El12Tie07|FR831879    GGCAACGACC ATACCACGTT GAAAACACCA GTTCCCGTCC GATCACCGAA GTTAAGCAGC GTCGGGCCCG GTTAGTACTT GGATGGGTGA CCGCCTGGGA ACACCGGGTG CCGTTGTCAC
El12Tie12|FR831882    GGCAACGACC ATACCACGTT GAAAACACCA GTTCCCGTCC GATCACCGAA GTTAAGCAGC GTCGGGCCCG GTTAGTACTT GGATGGGTGA CCGCCTGGGA ACACCGGGTG CCGTTGTCAC
El12Tie08|FR831880    GGCAACGACC ATACCACGCT GAAAACACCA GTTCCCGTCC GATCACCGAA GTTAAGCAGC GTCGGGCCCG GTTAGTACTT GGATGGGTGA CCGCCTGGGA ACACCGGGTG CCGTTGTCAC
El06Tie07|FR831874    GGCAACGACC ATACCACGTT GAAAACACCA GTTCCCGTCC GATCACCGAA GTTAAGCAGC GTCGGGCCCG GTTAGTACTT GGATGGGTGA CCGCCTGGGA ACACCGGGTG CCGTTGTCAC
Po02Ben01|FR831819    GGCAACGACC ATACCACGTT GAAAACACCA GTTCCCGTCC GATCACCGAA GTTAAGCAGC GTCGGGCCCG GTTAGTACTT GGATGGGTGA CCGCCTGGGA ACACCGGGTG CCGTTGTCAC
El03Afu30|FR831855    GGCAACGACA ATATCACGTT GAAAACACCG GTTCCCGTCC GATCACCGAA GTTAAGCAGC GTCGGGCCCG GTTAGTACTT GGATGGGTGA CCGCCTGGGA ACACCGGGTG CCGTTGTCAC
El05Afu02|FR831859    GGCAACGACC ATACCACGTT GAAAACACCA GTTCCCGTCC GATCACCGAA GTTAAGCAGC GTCGGGCCCG GTTAGTACTT GGATGGGTGA CCGCCTGGGA ACACCGGGTG CCGTTGTCAC
El04Afu04a|FR831857   GGCAACGACC ATACCACGTT GAAAACACCA GTTCCCGTCC GATCACCGAA GTTAAGCAGC GTCGGGCCCG GTTAGTACTT GGATGGGTGA CCGCCTGGGA ACACCGGGTG CCGTTGTCAC
El12Tie09|FR831881    GGCAACGACC ATACCACGTT GAAAACACCA GTTCCCGTCC GATCACCGAA GTTAAGCAGC GTCGGGCCCG GTTAGTACTT GGATGGGTGA CCGCCTGGGA ACACCGGGTG CCGTTGTCAC
El04Afu04b|FR831857   GGCAACGACC ATACCACGCT GAAAACACCA GTTCCCGTCC GATCACTGAA GTTAAGCAGC GTCGGGCCCG GTTAGTACTT GGATGGGTGA CCGCCTGGGA ACACCGGGTG CCGTTGTCAC
Po15Bal17|FR831838    GGCAACGACC ATATCACGTT GAAAACACCG GTTCCCGTCC GATCACCGAA GTTAAGCAGC GTCGGGCCCG GTTAGTACTT GGATGGGTGA CCGCCTGGGA ACACCGGGTG CCGTTGTCAC
El01Tie01b|FR831862   GGCAACGACC ATACCACGCT GAAAACACCA GTTCCCGTCC GATCACTGAA GTTAAGCAGC GTCGGGCCCG GTTAGTACTT GGATGGGTGA CCGCCTGGGA ACACCGGGTG CCGTTGTCAC
El01Tie03a|FR831864   GGCAACGACC ATACCACGTT GAAAACACCA GTTCCCGTCC GATCACCGAA GTTAAGCAGC GTCGGGCCCG GTTAGTACTT GGATGGGTGA CCGCCTGGGA ACACCGGGTG CCGTTGTCAC
El01Tie01a|FR831862   GGCAACGACC ATACCACGTT GAAAACACCA GTTCCCGTCC GATCACCGAA GTTAAGCAGC GTCGGGCCCG GTTAGTACTT GGATGGGTGA CCGCCTGGGA ACACCGGGTG CCGTTGTCAC
El01Tie03b|FR831864   GGCAACGACC ATACCACGCT GAAAACACCA GTTCCCGCCC GATCACTGAA GTTAAGCAGC GTCGGGCCCG GTTAGTACTT GGATGGGTGA CCGCCTGGGA ACACCGGGTG CCGTTGTCAC
El01Tie12|FR831867    GGCAACGACC ATACCACGTT GAAAACACCA GTTCCCGTCC GATCACCGAA GTTAAGCAGC GTCGGGCCCG GTTAGTACTT GGATGGGTGA CCGCCTGGGA ACACCGGGTG CCGTTGTCAC
El01Tie21|FR831868    GGCAACGACC ATACCACGTT GAAAACACCA GTTCCCGTCC GATCACCGAA GTTAAGCAGC GTCGGGCCCG GTTAGTACTT GGATGGGTGA CCGCCTGGGA ACACCGGGTG CCGTTGTCAC
El01Tie06|FR831865    GGCAACGACC ATACCACGTT GAAAACACCA GTTCCCGTCC GATCACCGAA GTTAAGCAGC GTCGGGCCCG GTTAGTACTT GGATGGGTGA CCGCCTGGGA ACACCGGGTG CCGTTGTCAC
Clustal Consensus     *********  *** **** * *********  ******  ** ****** *** ********** ********** ********** ******* ** ********** ********** *** ******


                      ....|....| ....|....| ....|....| ....|....| ....|....| ....|....| ....|....| ....|....| ....|....| ....|....| ....|....| ....|....|
                              130        140        150        160        170        180        190        200        210        220        230
240
El04Tie01|FR831870    ATTTTGCGCG CTTTCCTTTC AGCGGC--CA CCAACGAGAG CCGGTG--CA CATCACGGCC GTCTAAATCG CGCATCGTTT GTAACCGGCG TTCTTCGTAA GTGGTAACGC TGCTGTGCTT
El04Tie06a|FR831872   ATTTTGCGCG CTTTCCTTTC AGCGGC--CA CCAACGAGAG CCGGTG--CA CATCACGGCC GTCTAAATCG CGCATCGTTT GTAACCGGCG TTCTTCGTAA GTGGTAACGC TGCTGTGCTT
El04Tie03|FR831871    ATTTTGCGCG CTTTCCTTTC AGCGGC--CA CCAACGAGAG CCGGTG--CA CATCACGGCC GTCTAAATCG CGCATCGTTT GTAACCGGCG TTCTTCGTAA GTGGTAACGC TGCTGTGCTT
El04Tie09b|FR831873   ATTTTGCGCG CTTTCCTTTC AGCGGC--CA CCAACGAGAG CCGGTG--CA CATCACGGCC GTCTAAATCG CGCATCGTTT GTAACCGGCG TTCTTCGTAA GTGGTAACGC TGCTGTGCTT
El04Tie09a|FR831873   ATTTTGCGCG CTTTCCTTTC AGCGGC--CA CCAACGAGAA GCGGTG--CA CATCACGGCC GTCCAAATCG TGTATCGTTT GTAACCGGCG TTCTTCGTAA GTGGTAACGC TGCTGTGCTT
El05Afu03|FR831860    ATTTTGCGCG CTTTCCTTCC AGCGGC--CA CCAACGAGAG CCGGTG--CA CATCACGGCC GTCTAAATCG CGCATCGTTT GTAACCGGCG CTCTTCGTAA GTGGTAACGC TGCTGTGCTT
El04Tie06b|FR831872   ATTTTGCGCG CTTTCCTTTC AGCGGC--CA CCAACGAGAG CCGGTG--CA CATCACGGCC GTCTAAATCG CGCATCGTTT GTAACCGGCG TTCTTCGTAA GTGGTAACGC TGCTGTGCTT
El07Tie01|FR831875    ATTTTGCGCG CTTGCCTTCC AGCGGC--CA CCAACGAGAG CCGGTG--CA CATCACGGCC GTCTAAATCG CGTATCGTTT GTAACCGGCG TTCTTCGTAA GTGGTAACGC TGCTGTGCTT
El03Afu15|FR831852    ATTTTGCGCG CTTTCCTTTC AGCGGC--CA CCAACGAGAA GCGGTG--CA CATCACGGCC GTCCAAATCG TGTATCGTTT GTAACCGGCG TTCTTCGTAA GTGGTAACGC TGCTGTGCTT
El03Afu31|FR831856    ATTTTGCGCG CTTTCCTTTC AGCGGC--CA CCAACGAGAA GCGGTG--CA CATCACGGCC GTCCAAATCG TGTATCGTTT GTAACCGGCG TTCTTCGTAA GTGGTAACGC TGCTGTGCTT
El03Afu02a|FR831850   ATTTTGCGCG CTTTCCTTTC AGCGGC--CA CCAACGAGAA GCGGTG--CA CATCACGGCC GTCCAAATCG TGTATCGTTT GTAACCGGCG TTCTTCGTAA GTGGTAACGC TGCTGTGCTT
El03Afu07a|FR831851   ATTTTGCGCG CTTTCCTTTC AGCGGC--CA CCAACGAGAA GCGGTG--CA CATCACGGCC GTCCAAATCG TGTATCGTTT GTAACCGGCG TTCTTCGTAA GTGGTAACGC TGCTGTGCTT
El03Afu07b|FR831851   ATTTTGCGCG CTTTCCTTTC AGCGGC--CA CCAACGAGAA GCGGTG--CA CATCACGGCC GTCCAAATCG TGTATCGTTT GTAACCGGCG TTCTTCGTAA GTGGTAACGC TGCTGTGCTT
El03Afu16b|FR831853   ATTTTGCGCG CTTTCCTTTC AGCGGC--CA CCAACGAGAA GCGGTG--CA CATCACGGCC GTCCAAATCG TGTATCGTTT GTAACCGGCG TTCTTCGTAA GTGGTAACGC TGCTGTGCTT
El09Tie06|FR831878    ATTTTGCGCG CTTTCCTTCC AGCGGC--CA CCAACGAGAG CCGGCCGTCA CATCACGGCC GTCTAAATCG TGTATCGTTT GTAACCGGCG TTCTTCGTAA GTGGTAACGC TGCTGTGCTT
El02Tie01|FR831869    ATTTTGCGCG CTCTCCTTTC AGCGGC--CA CCAACGAGAG CCGGTG--CA CATCACGGCC GTCTAAATCG TGTATCGTTT GTAACCGGCG TTCTTCGTAA GTGGTGGCGC TGCTGTGCTT
El07Tie02|FR831876    ATTTTGCGCG CTTGCCTTCC AGCGGC--CA CCAACGAGAG CCGGTG--CA CATCACGGCC GTCTAAATCG CGTATCGTTT GTAACCGGCG CTCTTCGTCA GTGGTAACGC TGCTGTGCTT
El05Afu04a|FR831861   ATTTTGCGCG CTTTCCTTGC AGCGTG--CA CCAACGAGAG CCGGTG--CA CATCACGGCC GTCTAAATCG CGCATCGTTT GTAACCGGCG CTCTTCGTAA GTGGTAGCGC TGCTGTGCTT
El05Afu04b|FR831861   ATTTTGCGCG CTTTCCTTGC AGCGTG--CA CCAACGAGAG CCGGTG--CA CATCACGGCC GTCTAAATCG CGCATCGTTT GTAACCGGCG CTCTTCGTAA GTGGTAGCGC TGCTGTGCTT
El04Afu05|FR831858    ATTTTGCGCG CTTTCCTTGC AGCGTG--CA CCAACGAGAG CCGGTG--CA CATCACGGCC GTCTAAATCG CGCATCGTTT GTAACCGGCG CTCTTCGTAA GTGGTAACGC TGCTGTGCTT
El03Afu16a|FR831853   ATTTTGCGCG CTCCCCTTCC AGCGTG--CA CCAACGAGAG CCGGTG--CA CATGACGGCC GTCTAAATCG CGTATCGTTT GTAACCGGCG CTCTTCGTAA GTGGTAACGC TGCTGTGCTT
El03Afu19a|FR831854   ATTTTGCGCG CTCCCCTTCC AGCGTG--CA CCAACGAGAG CCGGTG--CA CATGACGGCC GTCTAAATCG CGTATCGTTT GTAACCGGCG CTCTTCGTAA GTGGTAACGC TGCTGTGCTT
El03Afu02c|FR831850   ATTTTGCGCG CTCCCCTTCC AGCGTG--CA CCAACGAGAG CCGGTG--CA CATGACGGCC GTCTAAATTG CGTATCGTTT GTAACCGGCG CTCTTCGTAA GTGGTAACGC TGCTGTGCTT
El09Tie01|FR831877    ATTTTGCGCG CTTTCCTTGC AGCGTG--CA CCAACGAGAG CCGGTG--CA CATCACGGCC GTCTAAATCG CGTATCGTTT GTAACCGGCG TTCTTCGTAA GTGGTAACGC TGCTGTGCTT
El01Tie10|FR831866    ATTTTGCGCG CTTTCCTTCC AGCGTG--CA CCAACGAGAG CCGGTG--CA CATCACGGCC GTCTAAATCG CGTAGCGTTT GTAACCGGCG TTCTTCGTAA GTGGTAACGC TGCTGTGCTT
El01Tie02a|FR831863   ATTTTGCGCG CTTTCCTTCC AGCGTG--CA CCAACGAGAG CCGGTG--CA CATCACGGCC GTCTAAATCG CGTAGCGTTT GTAACCGGCG TTCTTCGTAA GTGGTAACGC TGCTGTGCTT
Po02Ben02|FR831820    ATTTTGCGCG CTTTCCTTCC AGCGTG--CA CCAACGAGAG CCGGTG--CA CATCACGGCC GTCTAAATCG CGTAGCGTTT GTAACCGGCG TTCTTCGTAA GTGGTAACGC TGCTGTGCTT
Po02Ben03|FR831821    ATTTTGCGCG CTTTCCTTCC AGCGTG--CA CCAACGAGAG CCGGTG--CA CATCACGGCC GTCTAAATCG CGTAGCGTTT GTAACCGGCG TTCTTCGTAA GTGGTAACGC TGCTGTGCTT
El01Tie02b|FR831863   ATTTTGCGCG CTTTCCTTCC AGCGTG--CA CCAACGAGAG CCGGTG--CA CATCACGGCC GTCTAAATCG CGTAGCGTTT GTAACCGGCG TTCTTCGTAA GTGGTAACGC TGCTGTGCTT
El12Tie07|FR831879    ATTTTGCGCG CTTTCCTTGC AGCGTG--CA CCAACGAGAG CCGGTG--CA CATCGCGGCC GTCTAAATCG TGTATCGTTT GTAACCGGCG TTCTTCGTAA GTGGTAACGC TGCTGTGCTT
El12Tie12|FR831882    ATTTTGCGCG CTTTCCTTGC AGCGTG--CA CCAACGAGAG CCGGTG--CA CATCGCGGCC GTCTAAATCG TGTATCGTTT GTAACCGGCG TTCTTCGTAA GTGGTAACGC TGCTGTGCTT
```

```
El12Tie08|FR831880    ATTTTGCGCG CTTTCCTTGC AGCGTG--CA CCAACGAGAG CCGGTG--CA CATCGCGGCC GTCTAAATCG TGTATCGTTT GTAACCGGCG TTCTTCGTAA GTGGTAACGC TGCTGTGCTT
El06Tie07|FR831874    ATTTTGCGCG CTTTCCTTGC AGCGTGTGCA CCAACGAGAG CCGCTG--CA CATCACGGCC GTCTAGATCG CGTATCGTTT GTAACCGGCG TTCTTCGTAA GTGGTAACGC TGCTGTGCTT
Po02Ben01|FR831819    ATTTTGCGCG CTTTCCTTGC AGCGTG--CA CCAACGAGAG CCGGTG--CA CATCACGGCC GTCTAAATCG CGTAGCGTTT GTAACCGGCG TTCTTCGTAA GTGGTAACGC TGCTGTGCTT
El03Afu30|FR831855    ATTTTGCGCG CTTTCCTTGC AGCGTG--CA CCAACGAGAG CCGGTG--CA CATCACGGCC GTCTAAATCG CGTATCGTTT GTAACCGGCG TTCTTCGTAA GTGGTAACGC TGCTGTGCTT
El05Afu02|FR831859    ATTTTGCGCG CTCTCCTTCC AGCGTG--CA CCGACGAGAG CCGGTG--CA CATCACGGCC GTGTAAATCG CGCATCGTTT GTAACCGGCG TTCTTCGTAA GTGGTAACGC TGCTGTGCTT
El04Afu04a|FR831857   ATTTTGCGCG CTTTCCTTGC AGCGTG--CA CCAACGAGAG CCGGTG--CA CATCACGGCC GTGTAACTCA --TATCGTTT GCGACCGGCG TTCTTTGTAA GTGGTAACGC TGCTGTGCTT
El12Tie09|FR831881    ATTTTGCGCG CTTTCCTTGC AGCATG--CA CCAACGAGAG CCGGTG--CA CATCACGGCC GTGTAACTCA --TATCGTTT GCGACCGGCG TTCTTTGTAA GTGGTAACGC TGCTGTGCTT
El04Afu04b|FR831857   ATTTTGCGCG CTTTCCTTGC AGCGTG--CA CCAACGAGAG CCGGTG--CA CATCACGGCC GTGTAACTCA --TATCGTTT GCGACCGGCG TTCTTTGTAA GTGGTAACGC TGCTGTGCTT
Po15Bal17|FR831838    ATTTTGCGCG CTTTCCTTGC AGCGTG--CA CCAACGAGAG CCGGTG--CA CATCACGGCC GTGTAACTCA --TATCGTTT GCGACCGGCG TTCTTTGTAA GTGGTAACGC TGCTGTGCTT
El01Tie01b|FR831862   ATTTTGCGCG CTTTCCTTGC AGCGTG--CA CCAACGAGAG CCGGTG--CA CATCACGGCC GTGTAACTCA --TATCGTTT GCGACCGGCG TTCTTTGTAA GTGGTAACGC TGCTGTGCTT
El01Tie03a|FR831864   ATTTTGCGCG CTTTCCTTGC AGCGTG--CA CCAACGAGAG CCGGTG--CA CATCACGGCC GTGTAACTCA --TATCGTTT GCGACCGGTG TTCTTTGTAA GTGGTAACGC TGCTGTGCTT
El01Tie01a|FR831862   ATTTTGCGCG CTTTCCTTGC AGCGTG--CA CCAACGAGAG CCGGTG--CC CATCACGGCC GTGTAACTCA --TATCGTTT GCGACCGGCG TTCTTTGTAA GTGGTAACGC TGCTGTGCTT
El01Tie03b|FR831864   ATTTTGCGCG CTTTCCTTGC AGCGTG--CA CCAACGAGAG CCGGTG--CA CATCACGGCC GTGTAACTCA --TATCGTTT GCGACCGGTG TTCTTTGTAA GTGGTAACGC TGCTGTGCTT
El01Tie12|FR831867    ATTTTGCGCG CTTTCCTTGC AGCGTG--CA CCAACGAGAG CCGGTG--CA CATCACGGCC GTGTAACTCA --TATCGTTT GCGACCGGCG TTCTTTGTAA GTGGTAACGC TGCTGTGCTT
El01Tie21|FR831868    ATTTTGCGCG CTTTCCTTGC AGCGTG--CA CCAACGAGAG CCGGTG--CA CATCACGGCC GTGTAACTCA --TATCGTTT GCGACCGGCG TTCTTTGTAA GTGGTAACGC TGCTGTGCTT
El01Tie06|FR831865    ATTTTGCGCG CTTTCCTTGC AGCGTG--CA CCAACGAGAG CCGGTG--CA CATCACGGCC GTGTAACTCA --TATCGTTT GCGACCGGCG TTCTTTGTAA GTGGTAACGC TGCTGTGCTT
Clustal Consensus     ********** **  **** * ***      ** ** ******    **      *  ***  ***** **  *  *       * ***** *  ***** *  **** ** * *****  *** **********

                      ....|....| ....|....| ....|....| ....|....| ....|....| ....|....| ....|....| ....|....| ....|....| ....|....| ....|....| ....|....
                              250        260        270        280        290        300        310        320        330        340        350
El04Tie01|FR831870    TTGCAAGTTG CATAGCCCGA CAATTTCTCT TTAGAGAATA GATAGACGAC TAAATGACAA GACGTGACGT CACTTGTTGC TTTCG--AAG TCGCTCGGCC ACCGGCAATG CACGCTCTC
El04Tie06a|FR831872   TTGCAAGTTG CATAGCCCGA CAATTTCTCT TTAGAGAATA GATAGACGAC TAAATGACAA GACGTGACGT CACTTGTTGC TTTCG--AAG TCGCTCGGCC ACCGGCAATG CACGCTCTC
El04Tie03|FR831871    TTGCAAGTTG CATAGCCCGA CAATTTCTCT TTAGAGAATA GATAGACGAC TAAATGACAA GACGTGACGT CACTTGTTGC TTTCG--AAG TCGCTCGGCC ACCGGCAATG CACGCTCTC
El04Tie09b|FR831873   TTGCAAGTTG CATAGCCCGA CAATTTCTCT TTAGAGAATA GATAGACGAC TAAATGACAA GACGTGACGT CACTTGTTGC TTTCG--AAG TCGCTCGGCC ACCGGCAATG CACGCTCTC
El04Tie09a|FR831873   TTGCAAGTTG CATAGCCCGA CAATTTCTCT TTAGAGAATA GATAGACGAC TAAATGACAA GACGTGACGT CACTTGTTGC TTTCG--AAG TCGCTCGGCC ACCGGCAATG CACGCTCTC
El05Afu03|FR831860    TTGCAAGTTG CATAGCCCGA CAATTTCTCT TTAGAGAATA GATGGACGAC TAAATGACAA GACGTGACGT CACTTGTTGC TTTCG--AAG TCGCTCGGCC ACCGGCAATG CACGCTCTC
El04Tie06b|FR831872   TTGCAAGTTG CATAGCCCGA CAATTTCTCT TTAGAGAATA GATAGACGAC TAAGTGACAA GACGTGACGT CACTTGTTGC TTTCG--AAG TCGCTCGGCC ACCGGCAATG CACGCTCTC
El07Tie01|FR831875    TTGCAACTTG CATAGCCCGA CAATTTCTCT TTAGAGAATA GATAGACGAC TAAATGACAA GACGTGACGT CACTTGTTGC TTTCG--AAG TCGCTCGGCC ACCGGCAATG CACGCTCTC
El03Afu15|FR831852    TTGCAAGTTG CATAGCCCGA CAATTTTTCT CTAGAGAATA GATAGAAGAC TAAATGACAA GACGTGACGT CACTTGTTGC TCTCGCCAAG TCGGTCGGCC ACCAGCAATG CACGCTCTC
El03Afu31|FR831856    TTGCAAGTTG CATAGCCCGA CAATTTTTCT CTAGAGAATA GATAGAAGAC TAAATGACAA GACGTGACGT CACTTGTTGC TCTCGCCAAG TCGGTCGGCC ACCGGCAATG CACGCTCTC
El03Afu02a|FR831850   TTGCAAGTTG CATAGCCCGA CAATTTTTCT CTAGAGAATA GATAGAAGAC TAAATGACAA GACGTGACGT CACTTGTTGC TCTCGCCAAG TCGGTCGGCC ACCGGCAATG CACGCTCTC
El03Afu07a|FR831851   TTGCAAGTTG CATAGCCCGA CAATTTTTCT CTAGAGAATA GATAGAAGAC TAAATGACAA GACGTGACGT CACTTGTTGC TCTCGCCAAG TCGGTCGGCC ACCGGCAATG CACGCTCTC
El03Afu07b|FR831851   TTGCAAGTTG CATAGCCCGA CAATTTTTCT CTAGAGAATA GATAGAAGAC TAAATGACAA GACGTGACGT CACTTGTTGC TCTCGCCAAG TCGGTCGGCC ACCGGCAATG CACGCTCTC
El03Afu16b|FR831853   TTGCAAGTTG CATAGCCCGA CAATTTTTCT CTAGAGAATA GATAGAAGAC TAAATGACAA GACGTGACGT CACTTGTTGC TCTCGCCAAG TCGGTCGGCC ACCGGCAATG CACGCTCTC
El09Tie06|FR831878    TTGCAACTTG CATAGCCCGA CAATTTTTCT CCAGAGAATA GATAGAAGAC TAAATGACAT GACGTGACGT CACTTGTTGC TCTCGCCAAG TCGGTCGGCC ACCGGCAATG CACGCTCTC
El02Tie01|FR831869    TTGCAAGTTG CATAGCCCGA CAATTTTTCT CTAGAGACAA GATAGAAGAC TAAATGACAA GACGTGACGT CACTTGTTGC TCTCGCCAAG TCGCTCGGCC ACCGGGAATG CACGCTCTC
El07Tie02|FR831876    TTGCAAGTTG CATAGCCCGA CAATTTTTCT CTAGAGAATA GATAGAAGAC TAAATGACAA GACGTGACGT CACTTGTTGC TTTCG--AAG TCGGTCGGCC ACCGGCAATG CACACTCTC
El05Afu04a|FR831861   TTGCAACTTG CATAGCCCGA CAATTTCTCT TTAGAGAATA GATAGACGAC TAAATGACAA GACGTGACGT CACTTGTTGC TTTCG--AAG TCGCTCGGCC ACCGGCAATG CACGCTCTC
El05Afu04b|FR831861   TTGCAACTTG CATAGCCCGA CAATTTCTCT TTAGAGAATA GATAGACGAC TAAATGACAA GACGTGACGT CACTTGTTGC TTTCG--AAG TCGCTCGGCC ACCGGCAATG CACGCTCTC
El04Afu05|FR831858    TTGCAAGTTG CATAGCCCGA CAATTTCTCT TTAGAGAATA GATAGACGAC TAAATGACAA GACGTGACGT CACTTGTTGC TTTCG--AAG TCGCTCGGCC ACCGGCAATG CACGCTCTC
El03Afu16a|FR831853   TTGCAAGTTG CATAGCCCGA CAATTTCTCT TTAGAGAATA GATAGACGAC TAAATGACAA GACGTGACGT CACTTGTTGC TTTCG--AAG TCGCTCGGCC ACCGGCAATG CACGCTCTC
El03Afu19a|FR831854   TTGCAAGTTG CATAGCCCGA CAATTTCTCT TTAGAGAATA GATAGACGAC TAAATGACAA GACGTGACGT CACTTGTTGC TTTCG--AAG TCGCTCGGCC ACCGGCAATG CACGCTCTC
El03Afu02c|FR831850   TTGCAAGTTG CATAGCCCGA CAATTTCTCT TTAGAGAATA GATAGACGAC TAAATGACAA GACGTGACGT CACTTGTTGC TTTCG--AAG TCGCTCGGCC ACCGGCAATG CACGCTCTC
El09Tie01|FR831877    TTGCAAGTTG CATAGCCCGA CAATTTCTCT TTAGAGAATA GATAGACGAC TAAATGACAA GACGTGACGT CACTTGTTGC TTTCG--AAG TCGCTCGGCC ACCGGCAACG CACGCTCTC
El01Tie10|FR831866    TTGCAAGTTG CATAGCCCGA CAATTTCTCT TTAGAGAATA GATAGAGCAC TAAATGACAA GACGTGACGT CACTTGTTGC TTTCG--AAG TCGCTCGGCC ACCGGCAATG CACGCTCTC
El01Tie02a|FR831863   TTGCAAGTTG CATAGCCCGA CAATTTCTCT TTAGAGAATA GATAGAGCAC TAAATGACAA GACGTGACGT CACTTGTTGC TTTCG--AAG TCGCTCGGCC ACCGGCAATG CACGCTCTC
Po02Ben02|FR831820    TTGCAAGTTG CATAGCCCGA CAATTTCTCT TTAGAGAATA GATAGAGCAC TAAATGACAA GACGTGACGT CACTTGTTGC TTTCG--AAG TCGCTCGGCC ACCGGCAATG CACGCTCTC
Po02Ben03|FR831821    TTGCAAGTTG CATAGCCCGA CAATTTCTCT TTAGAGAATA GATAGAGCAC TAAATGACAA GACGTGACGT CACTTGTTGC TTTCG--AAG TCGCTCGGCC ACCGGCAATG CACGCTCTC
El01Tie02b|FR831863   TTGCAAGTTG CATAGCCCGA CAATTTCTCT TTAGAGAATA GATAGAGCAC TAAATGACAA GACGTGACGT CACTTGTTGC TTTCG--AAG TCGCTCGGCC ACCGGCAATG CACGCTCTC
El12Tie07|FR831879    TTGCAACTTG CATAGCCCGA CAATTTCTCT CTAGAGAATA GATAGAAGAC TAAATGACAA GACGTGACGT CACTTGTTGT TTTCG--AAG TCGCTCGGCC ACCGGCAATG CACGCTCTC
El12Tie12|FR831882    TTGCAACTTG CATAGCCCGA CAATTTCTCT CTAGAGAATA GATAGAAGAC TAAATGACAA GACGTGACGT CACTTGTTGC TTTCG--AAG TCGCTCGGCC ACCGGCAATG CGCGCTCTC
El12Tie08|FR831880    TTGCAACTTG CATAGCCCGA CAATTTCTCT CTAGAGAATA GATAGAAGAC TAAATGACAA GACGTGACGT CACTTGTCGC TTTCG--AAG TCGCTCGGCC ACCGGCAATG CACGCTCTC
El06Tie07|FR831874    TTGCAAGTTG CATAGCCCGA CAATTTCTCT CTAGAGAATA GATAGAACAC TAAATGACAA GACGTGACGT CACTTGTTGC TTTCG--AAG TCGCTCGGCC ACCGGCAATG CACGCTCTC
Po02Ben01|FR831819    TTGCAAGTTG CATAGCCCGA CAATTTTTCT CTAGAGAATA GATAGAAGAC TAAATGACAA GACGTGACGT CACTTGTTGC TTTCG--AAG TCGCTCGGCC ACCGGCAATG CACGCTCTC
El03Afu30|FR831855    TTGCAAGTTG CATAGCCCGA CAATTTTTCT CTGGAGAATA GATAGAAGAC TAAATGACAA GACGTGATGT CACTTGTTGC TTTCG--AAG TCGGTCGGCC ACCGGCAATG CACGCTCTC
El05Afu02|FR831859    TTGCAACTTG CATAGCCCGA CAATTTTTCT CTAGAGAATA GATAGAAGAC TAAATGACAA GACGTGACGT CACTTGTTGC TTTCG--AAG TCGGTCGGCC ACCGGCAATG CACGCTCTC
El04Afu04a|FR831857   TTGCAAGTTG CATAGCCCGA CAATTTTTCT CTAGAGAATA GATAGAAGAC TAAATGACAA GACGTGACGT CACTTGTTGC TCTCGCCAAG TCGGTCGGCC ACCGGCAATG CACGCTCTC
El12Tie09|FR831881    TTGCAACTTG CATAGCCCGA CAATTTTTCT CTAGAGACAA GATAGAAGAC TAAATGACAA GACGTGACGT CACTTGTTGC TCTCGCCAAG TCGGTCGGCC ACCGGCAATG CACGCTCTC
El04Afu04b|FR831857   TTGCAAGTTG CATAGCCCGA CAATTTTTCT CTAGAGAATA GATAGAAGAC TAAATGACAA GACGTGACGT CACTTGTTGC TCTCGCCAAG TCGGTCGGCC ACCGGCAATG CACGCTCTC
Po15Bal17|FR831838    TTGCAAGTTG CATAGCCCGA CAATTTTTCT CTAGAGAATA GATAGAAGAC TAAATGACAA GACGTGACGT CACTTGTTGC TCTCGCCAAG TCGGTCGGCC ACCGGCAATG CACGCTCTC
```

```
El01Tie01b|FR831862   TTGCAAGTTG CATAGCCCGA CAATTTTTCT CTAGAGAATA GATAGAAGAC TAAATGACAA GACGTGACGT CACTTGTTGC TTTCG--AAG TCGCTCGGCC ACCGGCAATG CACGCTCTC
El01Tie03a|FR831864   TTGCAACTTG CATAGCCCGA CAATTTTTCT CTAGACGATA GATAGAAGAC TAAATGACAA CACGTGACGT CACCTGTTGC TTTCG--AAG TCGCTCGGCC ACCGGCAATG CACGCTCTC
El01Tie01a|FR831862   TTGCAACTTG CATAGCCCGA CAATTTTTCT CTAGACAATA GATAGAAGAC TAAATGACAA CACGTGACGT CACCTGTTGC TTTCG--AAG TCGCTCGGCC ACCGGCAATG CACGCTCTC
El01Tie03b|FR831864   TTGCAACTTG CATAGCCCGA CAATTTTTCT CTAGACAATA GATAGAAGAC TAAATGACAA GACGTGACGT CACCTGTTGC TTTCG--AAG TCGCTCGGCC ACCGGCAATG CACGCTCTC
El01Tie12|FR831867    TTGCAACTTG CATAGCCCGA CAATTTTTCT CTAGACAATA GATAGAAGAC TAAATGACAA CACGTGACGT CACTTGTTGC TTTCG--AAG TCGCTCGGCC ACCGGCAATG CACGCTCTC
El01Tie21|FR831868    TTGCAAGTTG CATAGCCCGA CAATTTTTCT CTAGAGAATA GATAGAAGAC TAAATGACAA GACGTGACGT CACTTGTTGC TTTCG--AAG TCGCTCGGCC ACCGGCAATG CACGCTCTC
El01Tie06|FR831865    TTGCAAGTTG CATAGCCCGA CAATTTTTCT CTAGAGAATA GATAGAAGAC TAAATGACAA GACGTGACGT CACTTGTTGC TTTCG--AAG TCGCTCGGCC ACCGGCAATG CACGCTCTC
Clustal Consensus     ****** *** ********** ****** ***    **    * *** **  ** *** *****   ****** ** *** *** *  * ***  *** *** ****** *** * ** * * * *****
```

F and G types

```
                      ....|....| ....|....| ....|....| ....|....| ....|....| ....|....| ....|....| ....|....| ....|....| ....|....| ....|....| ....|....|
                              10         20         30         40         50         60         70         80         90        100        110
120
Py02Oly04a|FR831885   GGCAACGACC ATACCACGTT GAAAACACCA GTTCCCGTCC GATCACCGAA GTTAAGCAGC ATCGGGCCCG GTTAGTACTT GGATGGGTGA CCGCCTGGGA ACACCGGGTG CCGTTGTCAC
Py02Oly20|FR831886    GGCAACGACC ATACCACGTT GAAAACACCA GTTCCCGTCC GATCACCGAA GTTAAGCAGC ATCGGGCCCG GTTAGTACTT GGATGGGTGA CCGCCTGGGA ACACCGGGTG CCGTTGTCGC
Po14Bal12|FR831837    GGCAACGACC ATATCACGTT GAAAACACCG GTTCCCGTCC GATCACCGAA GTTAAGCAGC ATCGGGCCCG GTTAGTACTT GGATGGGTGA CCGCCTGGGA ACACCGGGTG CCGTTGTCAC
Po22Bal02|FR831839    GGCAACGACC ATATCACGTT GAAAACACCG GTTCCCGTCC GATCACCGAA GTTAAGCAGC ATCGGGCCCG GTTAGTACTT GGATGGGTGA CCGCCTGGGA ACACCGGGTG CCGTTGTCAC
Po12Bal03|FR831834    GGCAACGACC ATACCACGTT GAAAACACCA GTTCCCGTCC GATCACCGAA GTTAAGCAGC ATCGGGCCCG GTTAGTACTT GGATGGGTGA CCGCCTGGGA ACACCGGGTG CCGTTGTCAC
Po14Bal04|FR831836    GGCAACGACC ATACCACGTT GAAAACACCA GTTCCCGTCC GATCACCGAA GTTAAGCAGC ATCGGGCCCG GTTAGTACTT GGATGGGTGA CCGCCTGGGA ACACCGGGTG CCGTTGTCAC
Py07Oly02|FR831891    GGCAACGACC ATACCACGTT GAAAACACCA GTTCCCGTCC GATCACTGAA GTTAAGCAGC ATCGGGCCCG GTTAGTACTT GGATGGGTGA CCGCCTGGGA ACACCGGGTG CCGTTGTCAC
Py07Oly17|FR831896    GGCAACGACC ATACCACGTT GAAAACACCA GTTCCCGTCC GATCACTGAA GTTAAGCAGC ATCGGGCCCG GTTAGTACTT GGATGGGTGA CCGCCTGGGA ACACCGGGTG CCGTTGTCAC
Py07Oly14|FR831894    GGCAACGACC ATACCACGTT GAAAACACCA GTTCCCGTCC GATCACTGAA GTTAAGCAGC ATCGGGCCCG GTTAGTACTT GGATGGGTGA CCGCCTGGGA ACACCGGGTG CCGTTGTCAC
Py03Oly01b|FR831887   GGCAACGACC ATACCATGCT GAAAACACCA GTTCCCGTCC GATCACTGAA GTTAAGCAGC ATCGGGCCCG GTTAGTACTT GGATGGGTGA CCGCCTGGGA ACACCGGGTG CCGTTGTCAC
Py08Oly03b|FR831899   GGCAACGACC ATACCATGCT GAAAACACCA GTTCCCGTCC GATCACTGAA GTTAAGCAGC ATCGGGCCCG GTTAGTACTT GGATGGGTGA CCGCCTGGGA ACACCGGGTG CCGTTGTCAC
Py08Oly01|FR831897    GGCAACGACC ATACCACGTT GAAAACACCA GTTCCCGTCC GATCACCGAA GTTAAGCAGC ATCGGGCCCG GTTAGTACTT GGATGGGTGA CCGCCTGGGA ACACCGGGTG CCGTTGTCAC
Py03Oly02|FR831888    GGCAACGACC ATACCACGTT GAAAACACCA GTTCCCGTCC GATCACCGAA GTTAAGCAGC ATCGGGCCCG GTTAGTACTT GGATGGGTGA CCGCCTGGGA ACACCGGGTG CCGTTGTCAC
Py03Oly01a|FR831887   GGCAACGACC ATACCACGTT GAAAACACCA GTTCCCGTCC GATCACCGAA GTTAAGCAGC ATCGGGCCCG GTTAGTACTT GGATGGGTGA CCGCCTGGGA ACACCGGGTG CCGTTGTCAC
Py08OLY03a|FR831899   GGCAACGACC ATACCACGTT GAAAACACCA GTTCCCGTCC GATCACCGAA GTTAAGCAGC ATCGGGCCCG GTTAGTACTT GGATGGGTGA CCGCCTGGGA ACACCGGGTG CCGTTGTCAC
Py07Oly10|FR831893    GGCAACGACC ATACCACGTT GAAAACACCA GTTCCCGTCC GATCACCGAA GTTAAGCAGC ATCGGGCCCG GTTAGTACTT GGATGGGTGA CCGCCTGGGA ACACCGGGTG CCGTTGTCAC
Py07Oly15|FR831895    GGCAACGACC ATACCACGTT GAAAACACCA GTTCCCGTCC GATCACCGAA GTTAAGCAGC ATCGGGCCCG GTTAGTACTT GGATGGGTGA CCGCCTGGGA ACACCGGGTG CCGTTGTCAC
py06Oly03|FR831890    GGCAACGACC ATACCACGTT GAAAACACCA GTTCCCGTCC GATCACCGAA GTTAAGCAGC ATCGGGCCCG GTTAGTACTT GGATGGGTGA CCGCCTGGGA ACACCGGGTG CCGTTGTCAC
py08Oly02|FR831898    GGCAACGACC ATACCACGTT GAAAACACCA GTTCCCGTCC GATCACCGAA GTTAAGCAGC ATCGGGCCCG GTTAGTACTT GGATGGGTGA CCGCCTGGGA ACACCGGGTG CCGTTGTCAC
Py07Oly09|FR831892    GGCAACGACC ATACCACGTT GAAAACACCA GCTCCCGTCC GATCACCGAA GTTAAGCAGC ATCGGGCCCG GTTAGTACTT GGATGGGTGA CCGCCTGGGA ACACCGGGTG CCGTTGTCAC
Py04Oly03|FR831889    GGCAACGACC ATACCACGTT GAAAACACCA GTTCCCGTCC GATCACCGAA GTTAAGCAGC ATCGGGCCCG GTTAGTACTT GGATGGGTGA CCGCCTGGGA ACACCGGGTG CCGTTGTCAC
Py02Oly03|FR831884    GGCAACGACC ATACCACGTT GAAAACACCA GTTCCCGTCC GATCACCGAA GTTAAGCAGC GTCGGGCCCG GTTAGTACT- GGATGGGTGA CCGCCTGGGA ACACCGGGTG CCGTTGTCAC
Py02Oly02|FR831883    GGCAACGACC ATACCACGTT GAAAACACCA GTTCCCGTCC GATCACCGAA GTTAAGCAGC ATCGGGCCCG GTTAGTACTT GGATGGGTGA CCGCCTGGGA ACACCGGGTG CCGTTGTCAC
Py02Oly04b|FR831885   GGCAACGACC ATACCACGTT GAAAACACCA GTTCCCGTCC GATCACCGAA GTTAAGCAGC ATCGGGCCCG GTTAGTACTT GGATGGGTGA CCGCCTGGGA ACACCGGGTG CCGTTGTCAC
Clustal Consensus     ********** *** ** * * *********  * ******** ****** *** **********  ********* *********  ********** ********** ********** ******** *

                      ....|....| ....|....| ....|....| ....|....| ....|....| ....|....| ....|....| ....|....| ....|....| ....|....| ....|....| ....|....|
                             130        140        150        160        170        180        190        200        210        220        230
240
Py02Oly04a|FR831885   TTTTTCGGCC CTGTGCCAAC GGTGTCCACC GACCAGAGAC ACACCCGGCC GTGACATGAG CCACACTCAC CAGCAATGCT CCATTGTGGC GTGACGCTGC AGTGAGCC-- -ATCGCCGCA
Py02Oly20|FR831886    TTTTTCGGCC CTGTGCCAAC GGCGTCCACC GACCAGAGAC ACACCCGGCC GTGACATGAG CCACACTCAC CAGCAATGCT CCATTGTGGC GTGACGCTGC AGTGAGCC-- -ATCGCCGCA
Po14Bal12|FR831837    TTTTTCGGCC CTGTGCCAAC GGCGTCCACC GACCAGAGAC ACACCCGGCC GCGACATGAG CCACACTCAC CAGCAATGCT CCATTGTGGC GTGACGCTGC AGTGAGCC-- -ATCGCCGCA
Po22Bal02|FR831839    TTTTTCGGCC CTGTGCCAAC GGCGTCCACC GACCAGAGAC ACACCCGGCC GCGACATGAG CCACACTCAC CAGCAATGCT CCATTGTGGC GTGACGCTGC AGTGAGCC-- -ATCGCCGCA
Po12Bal03|FR831834    TTTTTCGGCC CTGTGCCAAC GGCGTCCACC GACCAGAGAC ACACCCGGCC GCGACATGAG CCACACTCAC CAGCAATGCT CCATTGTGGC GTGACGCTGC AGTGAGCC-- -ATCGCCGCA
Po14Bal04|FR831836    TTTTTCGGCC CTGTGCCAAC GGCGCCCACC GACCAGAGAC ACACCCGGCC GCGACATGAG CCACGCTCAC CAGCAATGCT CCATTGTGGC GTGACGCTGC AGTGAGCC-- -ATCGCCGCA
Py07Oly02|FR831891    TTTTTCGGCC CTGTGCCAAC GGCGTCCACC GACCAGAGAC ACACCCGGCT GTGACGTGAG CCACACTCAC CAGCAATGCT CCATTGTGGC GTGACGCTGC AGTGAGCC-- -ATCGCCGCA
Py07Oly17|FR831896    TTTTTCGGCC CTGTGCCAAC GGCGTCCACC GACCAGAGAC ACACCCGGCT GTGACGTGAG CCACACTCAC CAGCAATGCT CCATTGTGGC GTGACGCTGC AGTGAGCC-- -ATCGCCGCA
Py07Oly14|FR831894    TTTTTCGGCC CTGTGCCAAC GGCGTCCACC GACCAGAGAC ACACCCGGCT GTGACGTGAG CCACACTCAC CAGCAATGCT CCATTGTGGC GTGACGCTGC AGTGAGCC-- -ATCGCCGCA
Py03Oly01b|FR831887   TTTTTCGGCC CTGTGCCAAC GGCGTCCACC GACCAGAGAC ACACCCGGCT GTGACGTGAG CCACACTCAC CAGCAATGCT CCATTGTGGC GTGACGCTGC AGTGAGCC-- -ATCGCCGCA
Py08Oly03b|FR831899   TTTTTCGGCC CTGTGCCAAC GGCGTCCGCC GACCAGAGAC ACACCTGGCC GTGACATGAG CCACACTCAC CAGCAATGCT CCATTGTGGC GTGACGCTGC AGTGAGCC-- -ATCGCCGCA
```

```
Py08Oly01|FR831897    TTTTTCGGCC CTGTGCCAAC GGCGTCCGCC GACCAGAGAC ACACCCGGCC GTGACATGAG CCACACTCAC CAGCAATGCT CCATTGTGGC GTGACGCTGC AGTGAGCC-- -ATCGCCGCA
Py03Oly02|FR831888    TTTTTCGGCC CTGTGCCAAC GGCGTCCGCC GACCAGAGAC ACACCCGGCC GTGACATGAG CCACACTCAC CAGCAATGCT CCATTGTGGC GTGACGCTGC AGTGAGCC-- -ATCGCCGCA
Py03Oly01a|FR831887   TTTTTCGGCC CTGTGCCAAC GGCGTCCGCT GACCAGAGAC ACACCCGGCC GTGACATGAG CCACACTCAC CAGCAATGCT CCATTGTGGC GTGACGCTGC AGTGAGCC-- -ATCGCCGCA
Py08OLY03a|FR831899   TTTTTCGGCC CTGTGCCAAC GGCGTCCGCC GACCAGAGAC ACACCTGGCC GTGACATGAG CCACACTCAC CAGCAATGCT CCATTGTGGC GTGACGCTGC AGTGAGCC-- -ATCGCCGCA
Py07Oly10|FR831893    TTTTTCGGCC CTGTGCCAAC GGCGTCCGCC GACCAGAGAC ACACCCGGCC GTGACATGGG CCACACTCGC CAGCAATGCG CCATTGTGGC GTGACGCTGC AGTGAGCC-- -ATCGCCGCA
Py07Oly15|FR831895    TTTTTCGGCC CTGTGCCAAC GGCGTCCGCC GACCAGAGAC ACACCCGGCC GTGACATGGG CCACACTCGC CAGCAATGCG CCATTGTGGC GTGACGCTGC AGTGAGCC-- -ATCGCCGCA
py06Oly03|FR831890    TTTTTCGGCC CTGTGCCAAC GGCGTCCACC GACCAGAGAC ACACCCGGCC GTGACATGAG CCACACTCAC CAGCAATGCT CCATTGTGGC GTGACGCTGC AGTGAGCC-- -ATCGCCGCA
py08Oly02|FR831898    TTTTTCGGCC CTGTGCCAAC GGCGTCCACC GACCAGAGAC ACACCCGGCC GTGACATGAG CCACACTCAC CAGCAATGCT CCATTGTGGC GTGACGCTGC AGTGAGCC-- -ATCGCCGCA
Py07Oly09|FR831892    TTTTTCGGCC CTGTGCCAAC GCCGTCCACC GACCAGAGAC ACACCCGGCC GTGATATGAG CCACACTCAC CAGCAATGCT CCATTGTGGC GTGACGCTGC AGTGAGCC-- -ATCGCCGCA
Py04Oly03|FR831889    TTTTTCGGCC CTGTGCCAAC GGCGTCCACC GACCAGAGAC ACACCCGGCC GTGACATGAG CCACACTCGC CAGCAATGCT CCATTGTGGC GTGACGCTGC AGTGAGCC-- -ATCGTCGCA
Py02Oly03|FR831884    TTTTTCCGCC CTGTGCCAGC GGCGTCCACC GACGAGAGAC TCGCCTGGCC GTGACATGAG CTACACTCAC CGGCAATGCT CCATTGTGGC GTGACGCTGC AGTAAGCCGC CATCGCCGCA
Py02Oly02|FR831883    TTTTTCCGCC CTGTGCCAGC GGCGTCCACC GACGAGAGAC TCGCCTGGCC GTGACATGAG CTACACTCAC CGGCAATGCT CCATTGTGGC GTGACGCTGC AGTAAGCCGC CATCGCCGCA
Py02Oly04b|FR831885   TTTTTCCGCC CTGTGCCAGC GGCGTCCACC GACGAGAGAC TCGCCTGGCC GTGACATGAG CTACACTCAC CGGCAATGCT CCATTGTGGC GTGACGCTGC AGTAAGCCGC CATCGCCGCA
Clustal Consensus     ****** *** ******** * *  * ** *  *** ******  * ** ***  * **  ** * * ** *** * * *******  ********* ********* *** ****    **** ****


                      ....|....| ....|....| ....|....| ....|....| ....|....| ....|....| ....|....| ....|....| ....|....| ....|....| ....|....| ....|....|
                              250        260        270        280        290        300        310        320        330        340        350
360
Py02Oly04a|FR831885   CGGCCGCCGA CACATTGTTT TCTTTCTTGT TCTATTCTTT --TGTTTTTT CTCTGTTGCG AAGCAGCGTT GGGCTGCGCC CGTGCCAAC- ---------- -GACTGCACA TGCGAGACAC
Py02Oly20|FR831886    CGGCCGCCGA CACATTGTTT TCTTTCTTGT TCTATTCTTT --TGTTTTTT CTCTGTTGCG AAGCAGCGTT GCGCTGCGCC CGTGCCAAC- ---------- -GACTGCACA TGCGAGACAC
Po14Bal12|FR831837    CGGCCGCCGA CACATTGTTT TCTTTCTTGT TCTATTCTTT --TGTTTTTT CTCTGTTGCG AAGCAGCGTT GCGCTGCGCC CGTGCCAAC- ---------- -GACTGCACA TGCGAGACAC
Po22Bal02|FR831839    CGGCCGCCGA CACATTGTTT TCTTTCTTGT TCTATTCTTT --TGTTTTTT CTCTGTTGCG AAGCAGCGTT GCGCTGCGCC CGTGCCAAC- ---------- -GACTGCACA TGCGAGACAC
Po12Bal03|FR831834    CGGCCGCCGA CACATTGTTT TCTTTCTTGT TCTATTCTTT --TGTTTTTT CTCTGTTGCG AAGCAGCGTT GCGCTGCGCC CGTGCCAAC- ---------- -GACTGCACA TGCGAGACAC
Po14Bal04|FR831836    CGGCCGCCGA CACATTGTTT TCTTTCTTGT TCTATTCTTT --TGTTTTTT CTCTGTTGCG AAGCAGCGTT GCGCTGCGCC CGTGCCAAC- ---------- -GACTGCACA TGCGAGACAC
Py07Oly02|FR831891    CGGCCGCCGA CACATTGTTT TCTTTCTTGT TCTATTGTTT TTTGTTTTTT CTTTGCTGCG AAGCAGCGTT GCGCTGCGCC CGTGCCAAC- ---------- -GACTGCACA TGCGAGACAC
Py07Oly17|FR831896    CGGCCGCCGA CACATTGTTT TCTTTCTTGT TCTATTGTTT TTTGTTTTTT CTTTGTTGCG AAGCAGCGTT GCGCTGCGCC CGTGCCAAC- ---------- -GACTGCACA TGCGAGACAC
Py07Oly14|FR831894    CGGCCGCCGA CACATTGTTT TCTTTCTTGT TCTATTGTTT TTTGTTTTTT CTTTGTTGCG AAGCAGCGTT GCGCTGCGCC CGTGCCAAC- ---------- -GACTGCACA TGCGAGACAC
Py03Oly01b|FR831887   CGGCCGCCGA CACATTGTTT TCTTTCTTGT TCTATTGTTT TTTGTTTTTT CTTTGTTGCG AAGCAGCGTT GCGCTGCGCC CGTGCCAAC- ---------- -GACTGCACA TGCGAGACAC
Py08Oly03b|FR831899   CGGCCGCCGA CACATTGTTT TCTTTCTTGT TCTATTGTTT --TGTTTTTT CTTTGTTGCG AAGCAGCGTT GCGCTGCGCC CGTGCCAAC- ---------- -GACTGCACA TGCGAGACAC
Py08Oly01|FR831897    CGGCCGCCGA CACATTGTTT TCTTTCTTGT TCTATTGTTT --TGTTTTTT CTTTGTCGCG AAGCAGCGTT GCGCTGCGCC CGTGCCAAC- ---------- -GACTGCACA TGCGAGACAC
Py03Oly02|FR831888    CGGCCGCCGA CACATTGTTT TCTTTCTTGT TCTATTGTTT --TGTTTTTT CTTTGTTGCG AAGCAGCGTT GCGCTGCGCC CGTGCCAAC- ---------- -GACTGCACA TGCGAGACAC
Py03Oly01a|FR831887   CGGCCGCCGA CACATTGTTT TCTTTCTTGT TCTATTGTTT --TGTTTTTT CTTTGTTGCG AAGCAGCGTT GCGCTGCGCC CGTGCCAAC- ---------- -GACTGCACA TGCGAGACAC
Py08OLY03a|FR831899   CGGCCGCCGA CACATTGTTT TCTTTCTTGT TCTATTGTTT --TGTTTTTT CTTTGTTGCG AAGCAGCGTT GCGCTGCGCC CGTGCCAAC- ---------- -GACTGCACA TGCGAGACAC
Py07Oly10|FR831893    CGGCCGCCGA CACATTGTTT TCTTTCTTGT TCTATTGTTT --GGTTTTT- CTTTGTTGCG AAGCAGCGTT GCGCTGCGCC CGTGCCAACG ACTGCGACAA CGACTGCACA TGCCAGACAC
Py07Oly15|FR831895    CGGCCGCCGA CACATTGTTT TCTTTCTTGT TCTATTGTTT --GGTTTTTT CTTTGTTGCG AAGCAGCGTT GCGCTGCGCC CGTGCCAACG ACTGCGACAA CGACTGCACA TGCCAGACAC
py06Oly03|FR831890    CGGCCGCCGA CACATTGTTT TCTTTCTTGT TCTATTGTTT --TGTTTTTT CTTTGTTGCG AAGCAGCGTT GCGCTGCGCC CGTGCCAAC- ---------- -GACTGCACA TGCCAGACAC
py08Oly02|FR831898    CGGCCGCCGA CACATTGTTT TCTTTCTTGT TCTATTGTTT --TGTTTTTT CTTTGTTGCG AAGCAGCGTT GCGCTGCGCC CGTGCCAAC- ---------- -GACTGCACA TGCGAGACAC
Py07Oly09|FR831892    CGGCCGCCGA CACATTGTTT TCTTTCTTGT TCTATTCTTT --TGTTTTTT CTTTGTTGCG AAGCAGCGTT GCGCTGCGCC CGTGCCAAC- ---------- -GACTGCACA TGCGAGACAC
Py04Oly03|FR831889    CGGCCGCCGA CACATTGTTT TCTCTCTTGT TCTATTCTTT --TGTTTTTT CTTTGTTGCG AAGCAGCGTT GCGCTGCGCC CGTGCCAAC- ---------- -GACTGCACA TGCGAGACAC
Py02Oly03|FR831884    CGGCCGCCGA CACGCTGTTT TCATTCTTTT TCTATCGTTT --TGTTTTTT CTCTGTTGCG AAGCAGCGTT GCGCTGCGCC CGTGCCAAC- ---------- -GACTGCACA TGCGAGACAC
Py02Oly02|FR831883    CGGCCGCCGA CACGCTGTTT TCATTCTTTT TCTATCGTTT --TGTTTTTT CTCTGTTGCG AAGCAGCGTT GCGCTGCGCC CGTGCCAAC- ---------- -GACTGCACA TGCGAGACAC
Py02Oly04b|FR831885   CGGCCGCCGA CACGCTGTTT TCATTCTTTT TCTATCGTTT --TGTTTTTT CTCTGTTGCG AAGCAGCGTT GCGCTGCGCC CGTGCCAAC- ---------- -GACTGCACA TGCGAGACAC
Clustal Consensus     ********** ***   ***** **  **** * *****  ***    ******  ** **  *** ********** * ******** *********             ********* *** ******


                      ....|....| ....|....| ....|....| ....|....| ....|....| ....|....| ....|....| ....|....| ....|....| ....|....| ....|....| ....|....|
                              370        380        390        400        410        420        430        440        450        460        470
480
Py02Oly04a|FR831885   CAGTGCTATT TGCCTTTGCA CGCGCTACGC CTGCCCCTGG GGGCGCGAAC CGAACAGCGC AAAGGCATAT TCAGATCAAA GGCATATTCG CTC------- ---------- ----------
Py02Oly20|FR831886    CAGTGCTATT TGCCTTTGCA CGCGCTACGC CTGCCCCTGG GGGCGCGAAC CGAACAGCGC AAAGGCATAT TCAGATCAAA GGCATATTCG CTC------- ---------- ----------
Po14Bal12|FR831837    CAGTGCTATT TGCCTTTGCA CGCGCTACGC CTGCCCCTGG GGGCGCGAAC CGAACAGCGC AAAGGCATAT TCAGATCAAA GGCATATTCG CTC------- ---------- ----------
Po22Bal02|FR831839    CAGTGCTATT TGCCTTTGCA CGCGCTACGC CTGCCCCTGG GGGCGCGAAC CGAACAGCGC AAAGGCATAT TCAGATCATA GGCATATTCG CTA------- ---------- ----------
Po12Bal03|FR831834    CAGTGCTATT TGCCTTTGCA CGCGCTACGC CTGCCCCTGG GGGCGCGAAC CGAACAGCGC AAAGGCATAT TCAGATCAAA GGCATATTCG CTC------- ---------- ----------
Po14Bal04|FR831836    CAGTGCTATT TGCCTTTGCA CGCGCTACGC CTGCCCCTGG GGGCGCGAAC CGAACAGCGC AAAGGCATAT TCAGATCAAA GGCATATACG CTC------- ---------- ----------
Py07Oly02|FR831891    CAGTGTTATT TGCCTTTGCA CGCGCTACGC CTGCCCCTGG GGGCGCGAAC CGAACAGCGC AAAGGCATAT TCAGATCAAA GGCATATTCG CTC------- ---------- ----------
Py07Oly17|FR831896    CAGTGTTATT TGCCTTTGCA CGCGCTACGC CTGCCCCTGG GGGCGCGAAC CGAACAGCGC AAAGGCATAT TCAGATCAAA GGCATATTCG CTC------- ---------- ----------
Py07Oly14|FR831894    CAGTGTTATT TGCCTTTGCA CGCGCTACGC CTGCCCCTGG GGGCGCGAAC CGAACAGCGC AAAGGCATAT TCAGATCAAA GGCATATTCG CTC------- ---------- ----------
Py03Oly01b|FR831887   CAGTGTTATT TGCCTTTGCA CGCGCTACGC CTGCCCCTGG GGGCGCGAAC CGAACAGCGC AAAGGCATAT TCAGATCAAA GGCATATTCG CTC------- ---------- ----------
Py08Oly03b|FR831899   CAGTGTTATT TGCCTTTGCA CGCGCTACGC CTGCCCCTGG GGGCGCCAAC CGAACAGCGC AAAGGCATAT TCAGATCAAA GGCATATTCG CTC------- ---------- ----------
Py08Oly01|FR831897    CAGTGTTATT TGCCTTTGCA CGCGCTACGC CTGCCCCTGG GGGCGCCAAC CGAACAGCGC AAAGGCATAT TCAGATCAAA GGCATATTCG CTC------- ---------- ----------
```

```
Py03Oly02|FR831888    CAGTGTTATT TGCCTTTGCA CGCGCTACGC CTGCCCCTGG GGGCGCCAAC CGAACAGCGC AAAGGCATAT TCAGATCAAA GGCATATTCG CTC------- ---------- ----------
Py03Oly01a|FR831887   CAGTGTTATT TGCCTTTGCA CGCGCTACGC CTGCCCCTGG GGGCGCCAAC CGAACAGCGC AAAGGCATAT TCAGATCAAA GGCATATTCG CTC------- ---------- ----------
Py08OLY03a|FR831899   CAGTGTTATT TGCCTTCGCA CGCGCTACGC CTGCCCCTGG GGGCGCCAAC CGAACAGCGC AAAGGCATAT TCAGATCAAA GGCATATTCG CTC------- ---------- ----------
Py07Oly10|FR831893    CAGTGTTATT TGCCTTTGCA CGCGCTACGC CTGCCCCTGG GGGCGCGAAC CGAACAGCGC AAAGGCATAT TCAGATCAAA GGCATATTCG CTC------- ---------- ----------
Py07Oly15|FR831895    CAGTGTTATT TGCCTTTGCA CGCGCTACGC CTGCCCCTGG GGGCGCGAAC CGAACAGCGC AAAGGCATAT TCAGATCAAA GGCATATTCG CTC------- ---------- ----------
py06Oly03|FR831890    CAGTGTTATT TGCCTTGGCA CGCGCTACGC CTGCCCCTGG TGGCGCGAAC CGAACAGCGC AAAGGCGTAT TCAGATCAAA GGCATATTCG CTC------- ---------- ----------
py08Oly02|FR831898    CAGTGTTATT TGCCTTTGCA CGCGCTACGC CTGCCCCTGG GGGCGCCAGC CGAACAGCGC AAAGGCATAT TCAGATCAAA GGCGTATTCG CTC------- ---------- ----------
Py07Oly09|FR831892    CAGTGTGATT TGCCTTTGCA CGCGCTACGC CTGCCACTGG GCGCGCGAAC CGAACAGCGC AAAGGCATAT TCAGATCAAA GGCATATTCG CTC------- ---------- ----------
Py04Oly03|FR831889    CAGTGCTATT TGCCTTTGCA CGCGCTACGC CTGCCCCTGG GGGCGCCAAC CGAACAGCGC AAAGGCATAT TCAGATCAAA GGCATATTCG CTC------- ---------- ----------
Py02Oly03|FR831884    TAGTGCTATT TGCGTTTGCA CGCACCACGC ATGCCCCTGG CGGCGCCAAC CGAACAACGC AAGTGCATAT TCATGTTATT TGTCGCCAAA CACCAGGTCG AGAGCCAGGC CATGACCGTC
Py02Oly02|FR831883    TAGTGCTATT TGCGTTTGCA CGCACCACGC ATGCCCCTGG CGGCGCCAAC CGAACAACGC AAGTGCATAT TCATGTTATT TGTCGCCAAA CACCAGGTCG AGAGCCAGGC CATGGCCGTC
Py02Oly04b|FR831885   TAGTGCTATT TGCGTTTGCA CGCACCACGC ATGCCCCTGG CGGCGCCAAC CGAACAACGC AAGTGCATAT TCATGTTATT TGTCGCCAAA CACCAGGTCG AGAGCCAGGC CATGGCCGTC
Clustal Consensus      ****  *** *** ** *** *** * ****  **** ****   **** * * ****** *** **  ** *** ***  * *    *          *

                      ....|....| ....|....| ....|....| ....|....| ....|....| ....|....| ....|....| ....|....| ....|....| ....|....| ....|....| ....|....|
                              490        500        510        520        530        540        550        560        570        580        590
600
Py02Oly04a|FR831885   ---------- ---------- ---------- ---------- ---------- ---------- ---------- ---------- ---------- ---------- ---------- ----------
Py02Oly20|FR831886    ---------- ---------- ---------- ---------- ---------- ---------- ---------- ---------- ---------- ---------- ---------- ----------
Po14Bal12|FR831837    ---------- ---------- ---------- ---------- ---------- ---------- ---------- ---------- ---------- ---------- ---------- ----------
Po22Bal02|FR831839    ---------- ---------- ---------- ---------- ---------- ---------- ---------- ---------- ---------- ---------- ---------- ----------
Po12Bal03|FR831834    ---------- ---------- ---------- ---------- ---------- ---------- ---------- ---------- ---------- ---------- ---------- ----------
Po14Bal04|FR831836    ---------- ---------- ---------- ---------- ---------- ---------- ---------- ---------- ---------- ---------- ---------- ----------
Py07Oly02|FR831891    ---------- ---------- ---------- ---------- ---------- ---------- ---------- ---------- ---------- ---------- ---------- ----------
Py07Oly17|FR831896    ---------- ---------- ---------- ---------- ---------- ---------- ---------- ---------- ---------- ---------- ---------- ----------
Py07Oly14|FR831894    ---------- ---------- ---------- ---------- ---------- ---------- ---------- ---------- ---------- ---------- ---------- ----------
Py03Oly01b|FR831887   ---------- ---------- ---------- ---------- ---------- ---------- ---------- ---------- ---------- ---------- ---------- ----------
Py08Oly03b|FR831899   ---------- ---------- ---------- ---------- ---------- ---------- ---------- ---------- ---------- ---------- ---------- ----------
Py08Oly01|FR831897    ---------- ---------- ---------- ---------- ---------- ---------- ---------- ---------- ---------- ---------- ---------- ----------
Py03Oly02|FR831888    ---------- ---------- ---------- ---------- ---------- ---------- ---------- ---------- ---------- ---------- ---------- ----------
Py03Oly01a|FR831887   ---------- ---------- ---------- ---------- ---------- ---------- ---------- ---------- ---------- ---------- ---------- ----------
Py08OLY03a|FR831899   ---------- ---------- ---------- ---------- ---------- ---------- ---------- ---------- ---------- ---------- ---------- ----------
Py07Oly10|FR831893    ---------- ---------- ---------- ---------- ---------- ---------- ---------- ---------- ---------- ---------- ---------- ----------
Py07Oly15|FR831895    ---------- ---------- ---------- ---------- ---------- ---------- ---------- ---------- ---------- ---------- ---------- ----------
py06Oly03|FR831890    ---------- ---------- ---------- ---------- ---------- ---------- ---------- ---------- ---------- ---------- ---------- ----------
py08Oly02|FR831898    ---------- ---------- ---------- ---------- ---------- ---------- ---------- ---------- ---------- ---------- ---------- ----------
Py07Oly09|FR831892    ---------- ---------- ---------- ---------- ---------- ---------- ---------- ---------- ---------- ---------- ---------- ----------
Py04Oly03|FR831889    ---------- ---------- ---------- ---------- ---------- ---------- ---------- ---------- ---------- ---------- ---------- ----------
Py02Oly03|FR831884    GCTGCAGCTG GACCCCCGAA GCGTGCATGC GCTTGTCGGC CTCGCAAAGA GAGCTCACAG GCGCTTGCCA GCAGCGCACG CCGGTCGTCA TGCGCTCAAT GCCGGCTCAT TCGCGCGGCC
Py02Oly02|FR831883    GCTGCAGCTG GACCCCCGAA GCGTGCATGC GCTTGTCGGC CTCGCAAAGA GAGCTCACAG GCGCTTGCCA GCAGCGCACG CCGGTCGTCA TGCGCTCAAT GCCGGCTCAT TCGCGCGGCC
Py02Oly04b|FR831885   GCTGCAGCTG GACCCCCGAA GCGTGCATGC GCTTGTCGGC CTCGCAAAGA GAGCTCACAG GCGCTTGCCA GCAGCGCACG CCGGTCGTCA TGCGCTCAAT GCCGGCTCAT TCGCGCGGCC
Clustal Consensus

                      ....|....| ....|....
                              610
Py02Oly04a|FR831885   ---------- ---------
Py02Oly20|FR831886    ---------- ---------
Po14Bal12|FR831837    ---------- ---------
Po22Bal02|FR831839    ---------- ---------
Po12Bal03|FR831834    ---------- ---------
Po14Bal04|FR831836    ---------- ---------
Py07Oly02|FR831891    ---------- ---------
Py07Oly17|FR831896    ---------- ---------
Py07Oly14|FR831894    ---------- ---------
Py03Oly01b|FR831887   ---------- ---------
Py08Oly03b|FR831899   ---------- ---------
Py08Oly01|FR831897    ---------- ---------
Py03Oly02|FR831888    ---------- ---------
Py03Oly01a|FR831887   ---------- ---------
```

**Py08OLY03a|FR831899**	---------- ---------
**Py07Oly10|FR831893**	---------- ---------
**Py07Oly15|FR831895**	---------- ---------
**py06Oly03|FR831890**	---------- ---------
**py08Oly02|FR831898**	---------- ---------
**Py07Oly09|FR831892**	---------- ---------
**Py04Oly03|FR831889**	---------- ---------
**Py02Oly03|FR831884**	ACCGGCGACG CGTTCGCTC
**Py02Oly02|FR831883**	ACCGGCGACG CGTTCGCTC
**Py02Oly04b|FR831885**	ACCGGCGACG CGTTCGCTC
**Clustal Consensus**	

Additional File 3, Table S2: Values of similarity among NTS types.

NTS	A	B	C	D	E	F
**A**						
**B**	2.40					
**C**	0.11	6E-09				
**D**	0.001	*	*			
**E**	0.0003	*	0.64	1E-12		
**F**	0.0004	*	0.006	0.11	0.36	
**G**	9E-15	2.4	0.009	0.004	0.001	4E-118

Table values show the E-value parametre. * No significant similarity found. The length of the seed that initiates the alignment was 7.
