# Supplementary material for: Molecular organization and phylogenetic analysis of 5S rDNA in crustaceans of the genus Pollicipes reveal birth-and-death evolution and strong purifying selection
Source: BMC Evol Biol. 2011 Oct 17;11:304. doi: 10.1186/1471-2148-11-304 (PMC3215682; doi:10.1186/1471-2148-11-304)
Supplement: Additional file 4 — Figure S3: Phylogenetic relationships of 5S rDNA for the three main variants in Pollicipes species reconstructed by means of a maximun likelihood trees. Numbers on nodes represent bootstrap values based on 100 replicates. (a) Phylogeny of A type reconstruted by K81uf + I + G model. (b) Phylogeny of E type reconstruted by SYM + I + G model. (c) Phylogeny of F and G types reconstruted by HKY + I + G model. In (b) and (c), asterisks indicate P. pollicipes sequences. Figure S4: Identified regions (not aligned) of 78 nucleotides upstream the transcriptional start site of 5S ribosomal DNA. Three conserved regions were identified. Nucleotides shaded in blue share the motif CGGCCACCGGC, those shaded in red correspond to an AT rich region that it was located about -25 bp, and the TTC sequence (shared with Bombyx mori silkworms) is shaded in green color. Figure S5: 5S ribosomal RNA predicted secondary structures of barnacles. Structures correspond to the b and c types sequences that excluding the primer-annealing regions. (a-h) Sequences used in the predicted consensus secondary structures type I Pollicipes and (i) type II Pollicipes. ((a) El04Tie09b;(b) El03Afu02b; (c) Py02Oly04b; (d) El04Afu04b, El03Afu19b, El05Afu04b, El03Afu02c, El01Tie02b, El01Tie01b; (e) El01Tie03b; (f) Py08Oly03b, Py03Oly01b; (g) El03Afu07b, El04Tie06b; (h) El03Afu16b; (i) Po06Bal01b, Po06Bal02b. Figure S6: 5S alignment. Upper line is the consensus 5S rDNA gene. White boxes represent the internal control regions (ICRs) involved in the transcription of the D. melanogaster 5S rDNA, and grey shading areas correspond to the three sequence elements that regulate transcription activity of X. laevis 5S rDNA (box A, intermediate element, and box C, from left to right). The b and c types sequences represent the second and third unit of the array. [file 1471-2148-11-304-S4.PDF]

Figure S3: Phylogenetic relationships of 5S rDNA for the three main variants in *Pollicipes* species reconstructed by means of a maximun likelihood trees. Numbers on nodes represent bootstrap values based on 100 replicates. (a) Phylogeny of A type reconstruted by K81uf+I+G model. (b) Phylogeny of E type reconstruted by SYM+I+G model. (c) Phylogeny of F and G types reconstruted by HKY+I+G model. In (b) and (c), asterisks indicate *P. pollicipes* sequences.

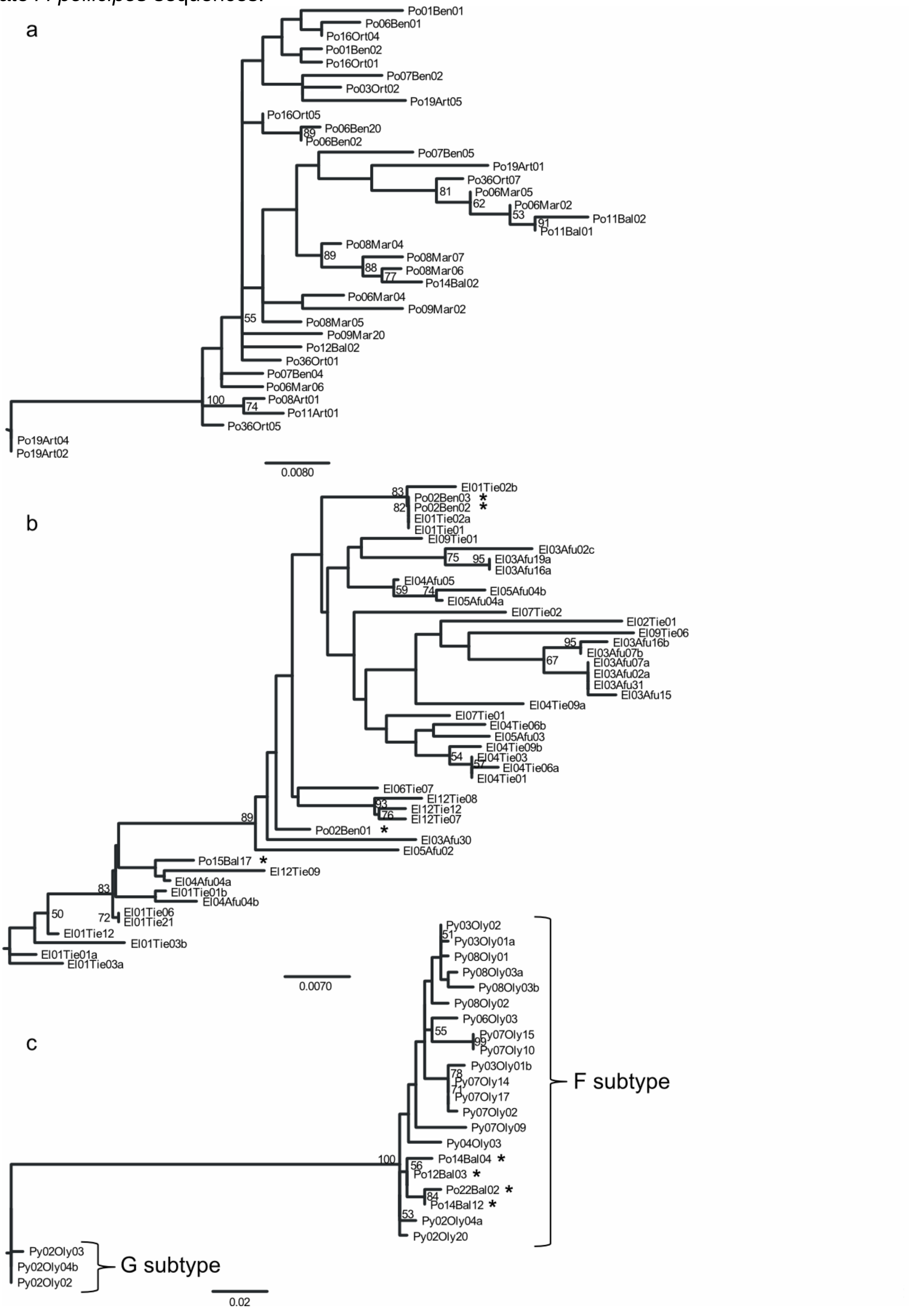

Figure S4: Identified regions (not aligned) of 78 nucleotides upstream the transcriptional start site of 5S ribosomal DNA. Three conserved regions were identified. Nucleotides shaded in blue share the motif CGGCCACCGGC, those shaded in red correspond to an AT rich region that it was located about -25 bp, and the TTC sequence (shared with *Bombyx mori* silkworms) is shaded in green color.

```
            -78·······*·········*·········*·········*·········*·········*·········*········1

Po11Bal01  . AAATATATGCGCAGCGCAGCCGGTTGTTATGCGCTGACGGCGGACGCATTCGCGCGGCCACCGGCAACGGATTCGCCC
Po11Bal02  . AATATATGCGCAGTGCACGCCGGTTGTTATGCGCTGACGGCGGACGCATTCGCGCGGCCACCGGCAACGGATTCGCCC
Po12Bal02  . GATATATGCGCAGCGCAGGCCGGTTGTCATGCGCTGACGGCGGACGCATTCGCGCGGCCACCGGCAACGAATTCGCCC
Po14Bal02  . AATATATGCGCAGCGCAGGCCGGTTGTCATGCGCTGACGGCGGACGCATTCGCGCGGCCACCGGCAACGAACTCGCCC
Po19Art01  . AATATATGCGCAGCGCAGGCCGGTTGTTATGCGCTGACGGCGAACGCATTCGCGCGGCCACCGGCAACGGATTCGCCC
Po19Art02  . AATATATGCGCAGCGCAGGCCGCTCGTCATGCGCTGATGGCGGTCGCATTCGCACGGCCACCGGCAACGGATCCGCCC
Po19Art04  . AATATATGCGCAGCGCAGGCCGCTCGTCATGCGCTGATGGCGGTCGCATTCGCACGGCCACCGGCAACGGATCCGCCC
Po19Art05  . AATATATGCGCAGCGCAGGCCGGTTGTCATGCGCTGACGGCGGACGCATTCGCGCGGCCACCGGCAACGAATTCGCCC
Po08Mar04  . AATATATGCGCAGCGCAAGCCGGTTGTCATGCGCTGACGGCGGACGCATTCGCGCGGCCACCGGCAACGAATTCGCCC
Po08Mar05  . AATATATGCGCAGCGCAGGCCGGTTGTCATGCGCTGACGGCGGACGCATTCGCGCGGCCACCGGCAACGAATTCGCCC
Po08Mar06  . AATATATGCGCAGCGCAGGCCGGTTGTCATGCGCTGACGGCGGACGCATTCGCGCGGCCACCGGCAACGAACTCGCCC
Po08Mar07  . AATATATGCGCAGCGCAGGCCGGTTGTCATGCGCTGACGGCGGACGCATTCGCGCGGCCACCGGCAACGAACTCGCCC
Po09Mar02  . AATATATGCGCAGCGCAGGCCGGTTGTCATGCGCTGACGGCGGACGCATTCGCGCGGCCACCGGCAACGAATTCGCCC
Po06Mar02  . AATATATGCGCAGCGCAGGCCGGTTGTTATGCGCTGACGGCGAACGCATTCGCGCGGCCACCGGCAACGGATTCGCCC
Po06Mar04  . AATATATGCGCAGCGCAGGACGGTTGTCATGCGCTGACGGCGGACGCATTCGCGCGGCCACCGGCAACAAATTCGCCC
Po06Mar05  . AATATATGCGCAGCGCAGGCCGGTTGTTATGCGCTGACGGCGAACGCATTCGCGCGGCCACCGGCAACGGATTCGCCC
Po06Mar06  . AATATATGCGCAGCGCAGGCCGGTTGTCATGCGCTGACGGCGGACGCATTCGCGCGGCCACCGGCAACGAATTCGCCC
Po16Ort04  . AATATATGCGCAGCGCAGGCCGGTTGTCATGCGCTGACGGCGGACGCATTCGCGCGGCCACCGGCAACGAATTCGCCC
Po11Arto1  . AATATATGCGCAGCGCAGGACGGTTGTCATGCGCTGACGGCGGACGCATTCGCGCGGCCACCGGCAACAAATTCGCCC
Po16Ort05  . AATATATGCGCAGCGCAGGCCGGTTGTCATGCGCTGACGGCGGACGCATTCGCGCGGCCACCGGCAACGAATTCGCCC
Po08Art01  . AATATATGCGCAGCGCAGGACGGTTGTCATGCGCTGACGGCGGACGCATTCGCGCGGCCACCGGCAACAAATTCGCCC
Po36Ort07  . AATATATGCGCAGCGCAGGCCGGTTGTTATGCGCTGACGGCGGACGCATTCGCGCGGCCACCGGCAACGGATTCGCCC
Po36Ort05  . AATATGTGCGCAGCGCAGGCCGGTTGTCATGCGCTGACGGCGGACGCATTCGCGCGGCCACCGGCAACGAATTCGCCC
Po36Ort01  . AATATGTGCGCAGCGCAGGCCGGTTGTCATGCGCTGACGGCGGACGCATTCGCGCGGCCACCGGCAACGAATTCGCCC
Po16Ort01  . AATATATGCGCAGCGCAGGCCGGTTGTCATGCGCTGACGGCGGACGCATTCGCGCGGCCACCGGCAACGAATTCGCCC
Po03Ort02  . AATATATGCGCAGCGCAGGCCGGTTGTCATGCGCTGACGGCGGACGCATTCGCGCGGCCACCGGCAACGAATTCGCCC
Po07Ben04  . AATATATGCGCAGCGCTGGCCGGTTGTCATGCGCTGACGGCGGACGCATTCGCGCGGCCACCGGCAACGAATTCGCCC
Po07Ben05  . AATATATGCGCAGCGCAGGCCGGTTGTCATGCGCTGACGGCGGACGCATTCGCGCGGCCACCGGCAACGGATTCGCCC
Po07Ben02  . AATATATGCGCAGCGCAGGCCGGTTGTCATGCGCTGACGGCGGACGCATTCGCGCGGCCACCGGCAACGAATTCGCCC
Po01Ben02  . AATATATGCGCAGCGCAGGCCGGTTGTCATGCGCTGACGGCGGACGCATTCGCGCGGCCACCGGCAACGAATTCGCCC
Po01Ben01  . AATATATGCGCAGCGTAGGCCGGTTGTCATGCGCTGACGGCGGACGCATTCGCGCGGCCACCGGCAACGAATTCGCCC
Po09Mar20  . AATATATGCGCAGCGCAGGCCGGTTGTCATGCGCTGACGGCGGACGCATTCGCGCGGCCACCGGCAACGAATTCGCCC
Po06Ben02  . AATATATGCGCAGCGCAGGCCGGTTGTCATGCGCTGACGGCGGACGCATTCGCGCGGCCACCGGCAACGAATTCGCCC
Po06Ben20  . AATATATGCGCAGCGCAGGCCGGTTGTCATGCGCTGACGGCGGACGCATTCGCGCGGCCACCGGCAACGAATTCGCCC
Po06Ben01  . AATATATGCGCAGCGCAGGCCGGTTGTCATGCGCTGACGGCGGACGCATTCGCGCGGCCACCGGCAACGAATTCGCCC
Po02Art01  . AACGACAAAAACCAAAAAAAAATCAATGAGCGAGTCAATTCAGGTAAGTATAACTACGGGTTCTGTTCACCTTCTCTC
Po17Art01  . AACGACAAAAACCAAAAAAAAATCAATGAGCGAGTCAATTCAGGTAAGTATAACTACGGGTTCTGTTCACCTTCTCTC
Po01Art01  . TAAAAACAAAACAAAAAAAAAAATCAATAAGCGGGTCAATACAGTAGTATAACTACGGGTTTTGTCCGCCTTCTCTC
Po06Bal01b . CTGGGCCCGTCTCCCCAGGGAATAAGACACAAAAAGAATTTGAGTAAGCACAAGTATGGGTTCTGTCCACCTTCTCTC
Po06Bal02b . CTGGGCCCGTCTCCCCAGGGAATAAGACACAAAAAGAATTTGAGTAAGCACAAGTATGGGTTCTGTCCACCTTCTCTC
Po06Bal04a . CTCGGCTCGTTTCCCGAGAGAAAAAGACACAAAAAGAATTTGAGTAGGTATAACTACGGGTTCTGTCCACCTTCTCTC
El03Afu02b . ATTTTGCGTGCCACATTTTGCGTAAATGTGCCACGTATTGTTTGCCAAGTTGGTCGGCCACCGGCAATGCACGCTCTC
El03Afu19b . ATTTTGCGTGCCACATTTTGCGTAAATGTGCCACGTATTGTTTGCCAAGTTGGTCGGCCACCGGCAATGCACGCTCTC
El04Afu04a . ATAGAAGACTAAATGACAAGACGTGACGTCACTTGTTGCTCTCGCCAAGTCGGTCGGCCACCGGCAATGCACGCTCTC
El09Tie06  . ATAGAAGACTAAATGACATGACGTGACGTCACTTGTTGCTCTCGCCAAGTCGGTCGGCCACCGGCAATGCACGCTCTC
El04Afu04b . ATAGAAGACTAAATGACAAGACGTGACGTCACTTGTTGCTCTCGCCAAGTCGGTCGGCCACCGGCAATGCACGCTCTC
El12Tie09  . ATAGAAGACTAAATGACAAGACGTGACGTCACTTGTTGCTCTCGCCAAGTCGGTCGGCCACCGGCAATGCACGCTCTC
El02Tie01  . ATAGAAGACTAAATGACAAGACGTGACGTCACTTGTTGCTCTCGCCAAGTCGCTCGGCCACCGGGAATGCACGCTCTC
El03Afu15  . ATAGAAGACTAAATGACAAGACGTGACGTCACTTGTTGCTCTCGCCAAGTCGGTCGGCCACCAGCAATGCACGCTCTC
El03Afu02a . ATAGAAGACTAAATGACAAGACGTGACGTCACTTGTTGCTCTCGCCAAGTCGGTCGGCCACCGGCAATGCACGCTCTC
El03Afu31  . ATAGAAGACTAAATGACAAGACGTGACGTCACTTGTTGCTCTCGCCAAGTCGGTCGGCCACCGGCAATGCACGCTCTC
El03Afu07a . ATAGAAGACTAAATGACAAGACGTGACGTCACTTGTTGCTCTCGCCAAGTCGGTCGGCCACCGGCAATGCACGCTCTC
El03Afu07b . ATAGAAGACTAAATGACAAGACGTGACGTCACTTGTTGCTCTCGCCAAGTCGGTCGGCCACCGGCAATGCACGCTCTC
El03Afu16b . ATAGAAGACTAAATGACAAGACGTGACGTCACTTGTTGCTCTCGCCAAGTCGGTCGGCCACCGGCAATGCACGCTCTC
Po15Bal17  . ATAGAAGACTAAATGACAAGACGTGACGTCACTTGTTGCTCTCGCCAAGTCGGTCGGCCACCGGCAATGCACGCTCTC
El04Tie09a . AGATAGACGACTAAATGACAAGACGTGACGTCACTTGTTGCTTTCGAAGTCGCTCGGCCACCGGCAATGCACGCTCTC
El04Tie09b . AGATAGACGACTAAATGACAAGACGTGACGTCACTTGTTGCTTTCGAAGTCGCTCGGCCACCGGCAATGCACGCTCTC
El05Afu04a . AGATAGACGACTAAATGACAAGACGTGACGTCACTTGTTGCTTTCGAAGTCGCTCGGCCACCGGCAATGCACGCTCTC
```

```
El05Afu03   . AGATGGACGACTAAATGACAAGACGTGACGTCACTTGTTGCTTTCGAAGTCGCTCGGCCACCGGCAATGCACGCTCTC
El04Afu05   . AGATAGACGACTAAATGACAAGACGTGACGTCACTTGTTGCTTTCGAAGTCGCTCGGCCACCGGCAATGCACGCTCTC
El05Afu04b . AGATAGACGACTAAATGACAAGACGTGACGTCACTTGTTGCTTTCGAAGTCGCTCGGCCACCGGCAATGCACGCTCTC
El05Afu02   . AGATAGAAGACTAAATGACAAGACGTGACGTCACTTGTTGCTTTCGAAGTCGGTCGGCCACCGGCAATGCACGCTCTC
El07Tie01   . AGATAGACGACTAAATGACAAGACGTGACGTCACTTGTTGCTTTCGAAGTCGCTCGGCCACCGGCAATGCACGCTCTC
El12Tie08   . AGATAGAAGACTAAATGACAAGACGTGACGTCACTTGTCGCTTTCGAAGTCGCTCGGCCACCGGCAATGCACGCTCTC
El06Tie07   . AGATAGAACACTAAATGACAAGACGTGACGTCACTTGTTGCTTTCGAAGTCGCTCGGCCACCGGCAATGCACGCTCTC
El12Tie07   . AGATAGAAGACTAAATGACAAGACGTGACGTCACTTGTTGTTTTCGAAGTCGCTCGGCCACCGGCAATGCACGCTCTC
El12Tie12   . AGATAGAAGACTAAATGACAAGACGTGACGTCACTTGTTGCTTTCGAAGTCGCTCGGCCACCGGCAATGCGCGCTCTC
El01Tie21   . AGATAGAAGACTAAATGACAAGACGTGACGTCACTTGTTGCTTTCGAAGTCGCTCGGCCACCGGCAATGCACGCTCTC
El01Tie12   . AGATAGAAGACTAAATGACAACACGTGACGTCACTTGTTGCTTTCGAAGTCGCTCGGCCACCGGCAATGCACGCTCTC
El01Tie10   . AGATAGAGCACTAAATGACAAGACGTGACGTCACTTGTTGCTTTCGAAGTCGCTCGGCCACCGGCAATGCACGCTCTC
El01Tie06   . AGATAGAAGACTAAATGACAAGACGTGACGTCACTTGTTGCTTTCGAAGTCGCTCGGCCACCGGCAATGCACGCTCTC
El01Tie03a . AGATAGAAGACTAAATGACAACACGTGACGTCACCTGTTGCTTTCGAAGTCGCTCGGCCACCGGCAATGCACGCTCTC
El01Tie03b . AGATAGAAGACTAAATGACAAGACGTGACGTCACCTGTTGCTTTCGAAGTCGCTCGGCCACCGGCAATGCACGCTCTC
El01Tie02a . AGATAGAGCACTAAATGACAAGACGTGACGTCACTTGTTGCTTTCGAAGTCGCTCGGCCACCGGCAATGCACGCTCTC
El01Tie02b . AGATAGAGCACTAAATGACAAGACGTGACGTCACTTGTTGCTTTCGAAGTCGCTCGGCCACCGGCAATGCACGCTCTC
El01Tie01a . AGATAGAAGACTAAATGACAACACGTGACGTCACCTGTTGCTTTCGAAGTCGCTCGGCCACCGGCAATGCACGCTCTC
El01Tie01b . AGATAGAAGACTAAATGACAAGACGTGACGTCACTTGTTGCTTTCGAAGTCGCTCGGCCACCGGCAATGCACGCTCTC
El09Tie01   . AGATAGACGACTAAATGACAAGACGTGACGTCACTTGTTGCTTTCGAAGTCGCTCGGCCACCGGCAACGCACGCTCTC
El04Tie01   . AGATAGACGACTAAATGACAAGACGTGACGTCACTTGTTGCTTTCGAAGTCGCTCGGCCACCGGCAATGCACGCTCTC
El04Tie03   . AGATAGACGACTAAATGACAAGACGTGACGTCACTTGTTGCTTTCGAAGTCGCTCGGCCACCGGCAATGCACGCTCTC
El04Tie06a . AGATAGACGACTAAATGACAAGACGTGACGTCACTTGTTGCTTTCGAAGTCGCTCGGCCACCGGCAATGCACGCTCTC
El04Tie06b . AGATAGACGACTAAGTGACAAGACGTGACGTCACTTGTTGCTTTCGAAGTCGCTCGGCCACCGGCAATGCACGCTCTC
El03Afu30   . AGATAGAAGACTAAATGACAAGACGTGATGTCACTTGTTGCTTTCGAAGTCGGTCGGCCACCGGCAATGCACGCTCTC
El03Afu02c . AGATAGACGACTAAATGACAAGACGTGACGTCACTTGTTGCTTTCGAAGTCGCTCGGCCACCGGCAATGCACGCTCTC
El03Afu16a . AGATAGACGACTAAATGACAAGACGTGACGTCACTTGTTGCTTTCGAAGTCGCTCGGCCACCGGCAATGCACGCTCTC
El03Afu19a . AGATAGACGACTAAATGACAAGACGTGACGTCACTTGTTGCTTTCGAAGTCGCTCGGCCACCGGCAATGCACGCTCTC
El07Tie02   . AGATAGAAGACTAAATGACAAGACGTGACGTCACTTGTTGCTTTCGAAGTCGGTCGGCCACCGGCAATGCACACTCTC
Po02Ben02   . AGATAGAGCACTAAATGACAAGACGTGACGTCACTTGTTGCTTTCGAAGTCGCTCGGCCACCGGCAATGCACGCTCTC
Po02Ben03   . AGATAGAGCACTAAATGACAAGACGTGACGTCACTTGTTGCTTTCGAAGTCGCTCGGCCACCGGCAATGCACGCTCTC
Po02Ben01   . AGATAGAAGACTAAATGACAAGACGTGACGTCACTTGTTGCTTTCGAAGTCGCTCGGCCACCGGCAATGCACGCTCTC
Py07Oly02   . TTGCACGCGCTACGCCTGCCCCTGGGGGCGCGAACCGAACAGCGCAAAGGCATATTCAGATCAAAGGCATATTCGCTC
Py07Oly14   . TTGCACGCGCTACGCCTGCCCCTGGGGGCGCGAACCGAACAGCGCAAAGGCATATTCAGATCAAAGGCATATTCGCTC
Py02Oly04a . TTGCACGCGCTACGCCTGCCCCTGGGGGCGCGAACCGAACAGCGCAAAGGCATATTCAGATCAAAGGCATATTCGCTC
Py08OLY03a . TCGCACGCGCTACGCCTGCCCCTGGGGGCGCCAACCGAACAGCGCAAAGGCATATTCAGATCAAAGGCATATTCGCTC
Py08Oly01   . TTGCACGCGCTACGCCTGCCCCTGGGGGCGCCAACCGAACAGCGCAAAGGCATATTCAGATCAAAGGCATATTCGCTC
py08Oly02   . TTGCACGCGCTACGCCTGCCCCTGGGGGCGCCAGCCGAACAGCGCAAAGGCATATTCAGATCAAAGGCGTATTCGCTC
py06Oly03   . TGGCACGCGCTACGCCTGCCCCTGGTGGCGCGAACCGAACAGCGCAAAGGCGTATTCAGATCAAAGGCATATTCGCTC
Py08Oly03b . TTGCACGCGCTACGCCTGCCCCTGGGGGCGCCAACCGAACAGCGCAAAGGCATATTCAGATCAAAGGCATATTCGCTC
Py03Oly02   . TTGCACGCGCTACGCCTGCCCCTGGGGGCGCCAACCGAACAGCGCAAAGGCATATTCAGATCAAAGGCATATTCGCTC
Py02Oly20   . TTGCACGCGCTACGCCTGCCCCTGGGGGCGCGAACCGAACAGCGCAAAGGCATATTCAGATCAAAGGCATATTCGCTC
Py04Oly03   . TTGCACGCGCTACGCCTGCCCCTGGGGGCGCCAACCGAACAGCGCAAAGGCATATTCAGATCAAAGGCATATTCGCTC
Py03Oly01a . TTGCACGCGCTACGCCTGCCCCTGGGGGCGCCAACCGAACAGCGCAAAGGCATATTCAGATCAAAGGCATATTCGCTC
Py03Oly01b . TTGCACGCGCTACGCCTGCCCCTGGGGGCGCGAACCGAACAGCGCAAAGGCATATTCAGATCAAAGGCATATTCGCTC
Py07Oly10   . TTGCACGCGCTACGCCTGCCCCTGGGGGCGCGAACCGAACAGCGCAAAGGCATATTCAGATCAAAGGCATATTCGCTC
Py07Oly15   . TTGCACGCGCTACGCCTGCCCCTGGGGGCGCGAACCGAACAGCGCAAAGGCATATTCAGATCAAAGGCATATTCGCTC
Py07Oly09   . TTGCACGCGCTACGCCTGCCACTGGGCGCGCGAACCGAACAGCGCAAAGGCATATTCAGATCAAAGGCATATTCGCTC
Py07Oly17   . TTGCACGCGCTACGCCTGCCCCTGGGGGCGCGAACCGAACAGCGCAAAGGCATATTCAGATCAAAGGCATATTCGCTC
Po14Bal12   . TTGCACGCGCTACGCCTGCCCCTGGGGGCGCGAACCGAACAGCGCAAAGGCATATTCAGATCAAAGGCATATTCGCTC
Po12Bal03   . TTGCACGCGCTACGCCTGCCCCTGGGGGCGCGAACCGAACAGCGCAAAGGCATATTCAGATCAAAGGCATATTCGCTC
Po14Bal04   . TTGCACGCGCTACGCCTGCCCCTGGGGGCGCGAACCGAACAGCGCAAAGGCATATTCAGATCAAAGGCATATACGCTC
Po22Bal02   . TTGCACGCGCTACGCCTGCCCCTGGGGGCGCGAACCGAACAGCGCAAAGGCATATTCAGATCATAGGCATATTCGCTA
Py02Oly04b . CGCTTGCCAGCAGCGCACGCCGGTCGTCATGCGCTCAATGCCGGCTCATTCGCGCGGCCACCGGCGACGCGTTCGCTC
Py02Oly03   . CGCTTGCCAGCAGCGCACGCCGGTCGTCATGCGCTCAATGCCGGCTCATTCGCGCGGCCACCGGCGACGCGTTCGCTC
Py02Oly02   . CGCTTGCCAGCAGCGCACGCCGGTCGTCATGCGCTCAATGCCGGCTCATTCGCGCGGCCACCGGCGACGCGTTCGCTC
```

Figure S5: 5S ribosomal RNA predicted secondary structures of barnacles. Structures correspond to the b and c types sequences that excluding the primer-annealing regions. (a-h) Sequences used in the predicted consensus secondary structures type I *Pollicipes* and (i) type II *Pollicipes.* ((a) *El04Tie09b*;(b) *El03Afu02b*; (c) *Py02Oly04b*; (d) *El04Afu04b*, *El03Afu19b*, *El05Afu04b*, *El03Afu02c*, *El01Tie02b*, *El01Tie01b*; (e) *El01Tie03b*; (f) *Py08Oly03b*, *Py03Oly01b*; (g) *El03Afu07b*, *El04Tie06b*; (h) *El03Afu16b*; (i) *Po06Bal01b*, *Po06Bal02b.*

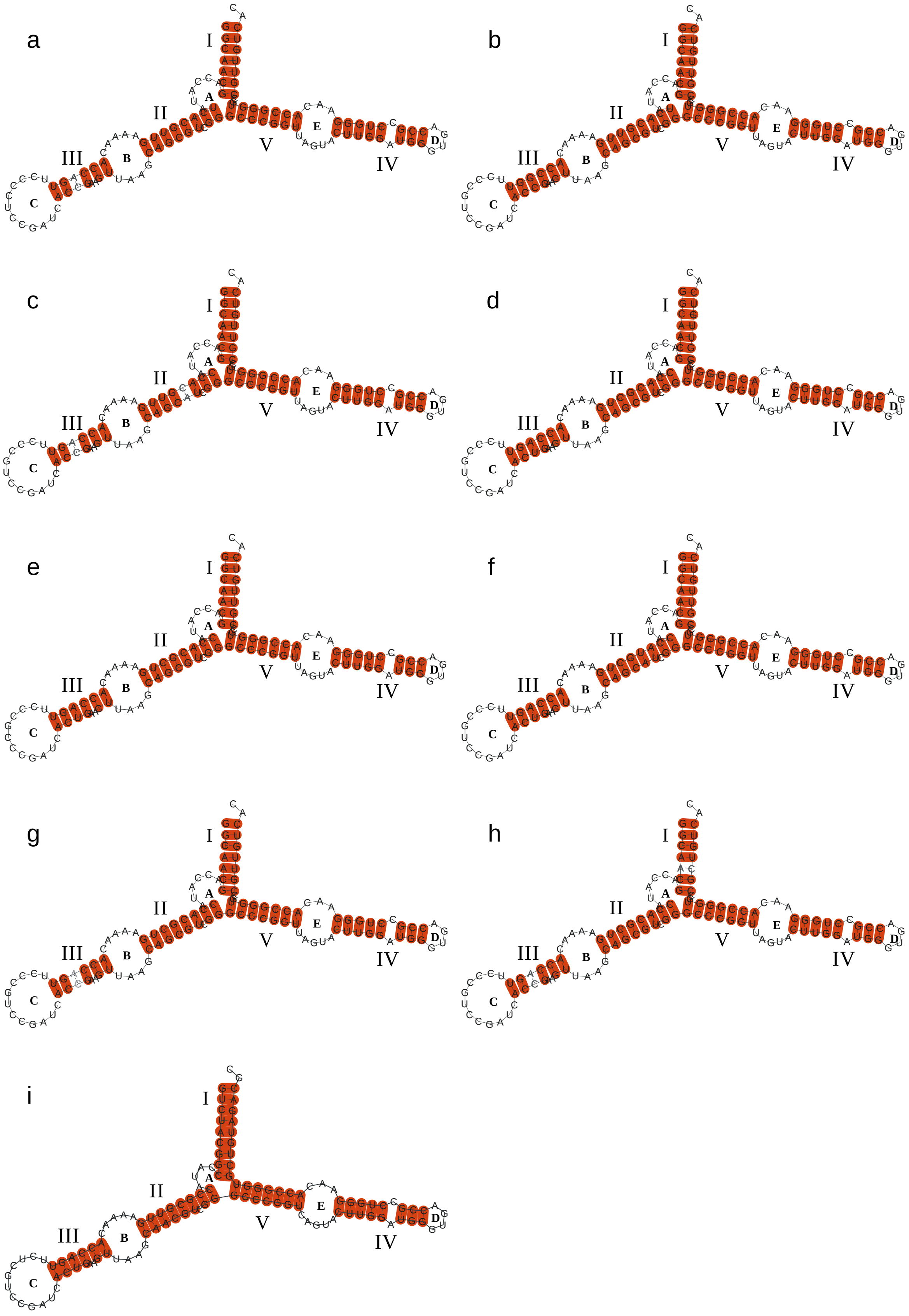

Figure S6: 5S alignment. Upper line is the consensus 5S rDNA gene. White boxes represent the internal control regions (ICRs) involved in the transcription of the *D. melanogaster* 5S rDNA, and grey shading areas correspond to the three sequence elements that regulate transcription activity of *X. laevis* 5S rDNA (box A, intermediate element, and box C, from left to right). The b and c types sequences represent the second and third unit of the array.

```
                          *        20         *        40         *        60         *        80         *       100         *       120
                   CaAcG CCATAcCacG TGAAA  ACC   TC CgtC GATC   GAaGT a  CA CgTcGgGcccgGT AGTACTTGGATGGGtGACCGCcTGGGAAcACcgggTGc gttG ca
                  [    ICR I     ]                  [ICR II]   [  ICR III   ]                [      ICR IV       ]
El01Tie03b     : GGCAACGACCATACCACGCTGAAAACACCAGTTCCCGCCCGATCACTGAAGTTAAGCAGCGTCGGGCCCGGTTAGTACTTGGATGGGTGACCGCCTGGGAACACCGGGTGCCGTTGTCAC : 120
El01Tie01b     : ........................................................................................................................ : 120
El01Tie02b     : ........................................................................................................................ : 120
El03Afu02c     : ........................................................................................................................ : 120
El04Afu04b     : ........................................................................................................................ : 120
El05Afu04b     : ........................................................................................................................ : 120
El03Afu19b     : ........................................................................................................................ : 120
El03Afu07b     : ..............................................C......................................................................... : 120
El03Afu16b     : ..............................................C..................................................................C...... : 120
El04Tie06b     : ..............................................C......................................................................... : 120
El04Tie09b     : .............T....T.................C.........C......................................................................... : 120
El03Afu02b     : .............T....T..........G................C......................................................................... : 120
Py02Oly04b     : ..............................................C.............A........................................................... : 120
Py08Oly03b     : ................T...........................................A........................................................... : 120
Py03Oly01b     : ................T...........................................A........................................................... : 120
Po06Bal01b     : .T.T...G.......G..T...............T.......................A.............C......................................T..A.A.G. : 120
Po06Bal02b     : .T.T...G.......G..T...............T.......................A.............C......................................T..A.A.G. : 120
P.coxalis      : .T.....G..........T.....G.....C...T..........GC...........A...T...T.T..................A.................A.A...T....G.TT : 120
A.aquaticus    : .T.....G..........T.....G.....C...T..........GC...........A...T...T.T..................A.................A.A...T....A.TT : 120
P.hawaiensis   : .C.....-..........T.....G....GC...T..........GC...........A...T...T.T....................................A.A...T....G.TT : 119
Artemia sp1    : AC.....G..........T.....GT...CAG..T....A....CTG.....C.CA..A.............C......................................T....G..T : 120
Artemia sp5    : AC.....G..........T.....GT...CAG..T....A....CTG.....C.CA..A.............C......................................T....GT.T : 120
Artemia sp2    : AC.....G..........T.....GT...CAG..T....A....CTG.....C.CA..A.............C......................................T....G..T : 120
Artemia sp4    : AC.....G..........T.....GT...CAG..T....A....CTG.....C.CA..A.............C......................................T....G..T : 120
Artemia sp3    : AC.....G..........T.....GT...CAG..T....A....CTG.....C.CA..A.............C......................................T....G..T : 120
Artemia sp6    : AC.....G........T.T.....GT...CAG..T....A....CTG..-..CCCA..A.............C.....................................TT....G..T : 119
C.finmarchicus : .T.T.G.G.....T..T.T...............T.......................A.A.T.A.TTGT........................T......T..ACAA..T.C.A.A..- : 119
```
