# Supplementary material for: Molecular organization and phylogenetic analysis of 5S rDNA in crustaceans of the genus Pollicipes reveal birth-and-death evolution and strong purifying selection
Source: BMC Evol Biol. 2011 Oct 17;11:304. doi: 10.1186/1471-2148-11-304 (PMC3215682; doi:10.1186/1471-2148-11-304)
Supplement: Additional file 5 — Constraints applied in RNA alifold. [file 1471-2148-11-304-S5.PDF]

Additional file 5:

Constraints applied in RNA alifold:

((((((.......((((((((xxxxx..((((xxxxxxxxxxxx))))....xxxx)))))).))(((((((.xxxx((..(..((....))..)..))...)))))))...))))))..
